# UBC/UBA52 silencing restores PINK1-Parkin-mediated mitochondrial autophagy in allergic rhinitis

**DOI:** 10.1371/journal.pone.0350815

**Published:** 2026-06-10

**Authors:** Qinhui Lu, Yanli Yang, Biao Ruan

**Affiliations:** 1 The First Affiliated Hospital of Kunming Medical University, Kunming, Yunnan, China; 2 The Affiliated Hospital of Yunnan University, Kunming, Yunnan, China; University of Tartu, ESTONIA

## Abstract

Allergic rhinitis (AR) is a common chronic inflammatory disease of the upper respiratory tract, and recent studies suggest that mitochondrial dysfunction may play a role in its pathogenesis. This study aimed to identify key genes related to AR and mitochondrial autophagy through bioinformatics analysis and to verify their functional roles in vitro. Transcriptomic data from the GEO database were analyzed, and ubiquitin C (UBC) and ubiquitin A-52 residue ribosomal protein fusion product 1 (UBA52) were identified as potential genes associated with AR and mitophagy. *In vitro*, IL-13–stimulated human nasal epithelial cells (HNEpCs) were used to establish an AR model. RT-qPCR and Western blotting showed that UBC and UBA52 were significantly upregulated, while mitophagy-related genes PINK1 and Parkin were downregulated. Flow cytometry and TMRE staining demonstrated increased ROS levels and reduced mitochondrial membrane potential (MMP), indicating mitochondrial dysfunction. Co-immunoprecipitation confirmed an interaction between UBC and UBA52. Silencing UBC downregulated UBA52 expression, restored PINK1 and Parkin levels, decreased ROS accumulation, and improved MMP, suggesting potential reactivation of the PINK1–Parkin–mediated mitophagy pathway. These findings suggest that UBC and UBA52 may be involved in the regulation of mitophagy and contribute to mitochondrial dysfunction in AR. Targeting the UBC–UBA52 axis may provide a novel therapeutic strategy for restoring mitochondrial homeostasis in allergic inflammation.

## Introduction

Allergic rhinitis (AR), a prevalent disease of the upper respiratory tract associated with nasal mucosal inflammation, is a heterogeneous condition commonly manifested by symptoms such as sneezing, nasal obstruction, rhinorrhea, and nasal itching [1]. With an estimated 400 million patients worldwide, AR exhibits a high incidence and an upward trend. This disease significantly impacts patients’ quality of life and imposes substantial socioeconomic burdens [2]. Treatment strategies for AR, including allergen avoidance, pharmacological therapy, and immunotherapy, further increase both individual and healthcare system costs. [3]. Consequently, the socioeconomic costs of treating AR are immense.

Mitochondrial autophagy is a specific mode of selective autophagy that selectively removes and phagocytoses damaged mitochondria under stress conditions. It has been shown that mitochondrial damage promotes the accumulation of PINK1 on the outer mitochondrial membrane, which recruits the cytoplasmic ubiquitin ligase Parkin to depolarized mitochondria and promotes mitophagy through ubiquitination of mitochondrial proteins and subsequent removal of damaged mitochondria, a process that is a key protective mechanism for the maintenance of mitochondrial homeostasis and normal cellular physiological functions [4,5]. Mitochondrial dysfunction has been shown to be associated with allergic diseases, including allergic dermatitis and asthma, etc [6]. Mitochondrial autophagy can inhibit mitochondria-induced inflammation; therefore, mitochondrial autophagy may play an important role in mitochondrial dysfunction and disorders of mitochondrial bioenergetics, and searching for a target to activate PINK1-Parkin-mediated mitochondrial autophagy to attenuate AR could help treat AR [7]. However, the specific molecular mechanisms and key regulatory genes underlying mitophagy in AR remain largely unclear.

The aim of this study was to identify the key genes related to AR and mitochondrial autophagy by transcriptome analysis of the GEO database, and to validate the critical role of PINK1-Parkin-mediated mitochondrial autophagy in AR by activation of PINK1-Parkin by UBC/UBA52 in combination with clinical and cellular experiments.

## Materials and methods

### Data acquisition

The GSE50223 dataset was obtained from the Gene Expression Omnibus (GEO) database. It includes peripheral blood samples from 21 individuals with AR, each of whom provided paired samples following diluent control or allergen stimulation. The dataset therefore contains a total of 42 samples: 21 allergen-challenged samples (AR group) and 21 diluent-treated samples (control group). The data were generated using the GPL6884 [Illumina HumanWG-6 v3.0 expression beadchip] platform. Raw expression values were normalized using log2(exp + 1) transformation. Gene annotation was performed based on the corresponding platform annotation file. These processed data were used for subsequent differential expression and enrichment analyses.

### Differential expression analysis between AR and control samples

Differential expression genes (DEGs) between AR and control samples were identified using the limma package in R. The screening criteria were set as an adjusted P-value < 0.05 and a log_2_ fold change (logFC) threshold of ≥ 0.5.

### GO and KEGG enrichment

DEGs identified in AR samples were subjected to Gene Ontology (GO) enrichment analysis, including biological process (BP), cellular component (CC), and molecular function (MF) categories. In addition, Kyoto Encyclopedia of Genes and Genomes (KEGG) pathway enrichment analysis was performed. Results were visualized as bubble plots generated using the clusterProfiler package. To preserve biological interpretability, GO terms were ordered according to their adjusted p-values rather than by numerical gene ratio. This visualization strategy follows common practice in enrichment analyses, as ranking by significance provides clearer biological prioritization. Gene ratio values were calculated as the proportion of input genes mapped to each GO term and plotted directly without reordering by magnitude.

### Protein-Protein Interaction (PPI)

The DEGs associated with AR were intersected with mitophagy-related genes obtained from the PathCards database. The overlapping genes were then analyzed using the STRING database to construct a PPI network, which was visualized using a confidence score threshold of 0.400.

### Cell culture

Human nasal mucosa epithelial cells (HNEpCs) (MZ-0964) were purchased from MingzhouBio (Ningbo, China). Cells were cultured in Roswell park memorial institute (RPMI) 1640 medium (11875093, Gibco, China) supplemented with 10% fetal bovine serum (FBS) (FSD500, Excell Bio, China), in an incubator at 37 °C with 5% CO₂ atmosphere. When cell confluence reached 80% or higher, HNEpCs were induced with 10 μg/L Interleukin (IL)-13 (90112ES10, Yeasen Biotechnology, Shanghai, China) to establish the AR model.

### Construction of sh-UBC lentiviral expression vector

The construction of plasmid sh-UBC (Suzhou Gemma Genetics) was to insert sh-UBC into pLKO.1 with siRNA construct. After construction, it was packaged into lentivirus using lentiviral packaging system. The constructed lentiviral expression vector was subsequently transfected into the target cells. The sh-UBC and its negative control (NC) sequence is provided below: UBC shRNA-1 forward, 5’-TCACTCACAGAAGTGCTTCTATTCAAGAGATAGAAGCACTTCTGTGAGTGA-3’, and reverse, 3’-GAAGCACTTCTGTGAGTGACCAAGTTCTCTGGTCACTCACAGAAGTGCTTC-5’; UBC shRNA-2 forward, 5’-TACCAGAACGGTCACTCACAGTTCAAGAGACTGTGAGTGACCGTTCTGGTA-3’, and reverse, 3’-GTGAGTGACCGTTCTGGTAGTAAGTTCTCTACTACCAGAACGGTCACTCAC-5’; UBC shRNA-3 forward, 5’-ACTACCAGAACGGTCACTCACTTCAAGAGAGTGAGTGACCGTTCTGGTAGT-3’, and reverse, 3’-GAGTGACCGTTCTGGTAGTGGAAGTTCTCTCCACTACCAGAACGGTCACTC-5’; sh-NC, 5’-CACCGTTCTCCGAACGTGTCACGTTTCAAGAGAACGTGACACGTTCGGAGAATTTTTTG-3’.

### Cell transfection

For lentiviral packaging, 250 μL of diluted Lipofectamine 3000 reagent (L3000015, Thermo Fisher, USA) was mixed with 250 μL of diluted plasmid DNA for each group and incubated for 15 min at room temperature. The resulting liposome–plasmid complexes were added to target cells for transfection. After 8 h, the transfection medium was removed and replaced with complete medium. The culture was continued for 48 h, after which the viral supernatant was collected, filtered through 0.45 μm microporous membrane, and stored at −80 °C.

For infection, UBC shRNA lentiviral particles were added to cells seeded in six-well plates along with polybrene to enhance transfection efficiency. After an 8 h incubation, the viral solution was replaced with fresh complete medium, and cells were further cultured for 24 h. Selection was performed by switching to complete medium containing puromycin. Stable UBC knockdown cell lines were established following puromycin selection.

### Real-time reverse transcription-quantitative polymerase chain reaction (RT-qPCR)

Cells were employed to extract total RNA with Trizol reagent (10296028; Invitrogen). RNA purity was assessed by the absorbance ratio at 260 and 280 nm. Detection through RT-qPCR was done with the Platinum SYBR Green qPCR SuperMix-UDG kit (Invitrogen, USA) using a QuantStudio 5 Real-Time PCR System (Applied Biosystems, USA). Gene expression levels were quantified using the 2^−ΔΔCt^ method alongside internal controls like Glyceraldehyde-3-phosphate dehydrogenase (GAPDH). All primer pairs were designed based on human mRNA sequences and their specificity was verified using NCBI Primer–BLAST. The primer sequence was provided below: UBC forward, 5’-CAGCCGGGATTTGGGTCG-3’, and reverse, 5’-CACGAAGATCTGCATTGTCAAGT-3’.

UBA52 forward, 5’-CGGACGCAAACATGCAGAT-3’; and reverse, 5’-CGGCAAATATCAGACGCTGC-3’; PINK1 forward, 5’-CCTGGAGTGTGAAACGCTCT-3’ and reverse, 5’-CTCCCACCCTCACCATTCAC-3’; Parkin forward, 5’-CTCAGGAGGTCACAGACACC-3’ and reverse, 5’-ACGCACAGGAAGACCTTGAA-3’; GAPDH forward, 5’-ATGGGCAGCCGTTAGGAAAG-3’ and reverse, 5’-ATCACCCGGAGGAGAAATCG-3’.

### Western blotting

Proteins were isolated by utilizing a cell lysis solution (Biosharp, China). The concentration of proteins was determined using the Bradford assay kit. After mixing thoroughly by vortex, equal amounts of proteins from each sample were analyzed by sodium dodecyl sulfate-polyacrylamide gel electrophoresis (SDS-PAGE) and transferred to polyvinylidene fluoride (PVDF) membranes (NCM Biotech, China). The PVDF membranes were then blocked with 5% skim milk and incubated overnight at 4 °C with antibodies targeting UBC (RRID:AB_2241301, 1:1000, proteintech, China, 10457–1-AP), UBA52 (AB_10861393, 1:1000, ab109227), PINK1 (AB_2927726, 1:1000, ab216144), Parkin (AB_1566559, 1:2000, ab77924), GAPDH (AB_2107448, 1:10000, ab8245) (Abcam, UK). Subsequently, a 2-hour incubation was carried out with a secondary antibody at a 1:20000 dilution (Bioss, China). The signal was visualized using an enhanced chemiluminescence kit (NCM Biotech, China).

### Flow cytometry

HNEpCs were seeded at a density of 1 × 10^6^ cells per 6 cm dish. Intracellular reactive oxygen species (ROS) levels were measured using the fluorescent probe DCFH-DA (10 μM; diluted 1:1000 in serum-free medium). For positive control wells, 3 μL of Rosup (50 mg/mL) was added to achieve a final concentration of 50 μg/mL.

After removing the cell culture medium, cells were incubated with 3 mL of DCFH-DA solution at 37 °C for 20 min. Following incubation, cells were washed three times with serum-free medium. Cells were then detached using trypsin, collected into centrifuge tubes, and resuspended in complete medium.After centrifugation at 1,000 rpm for 5 min, the supernatant was discarded. Cells were washed once with PBS and centrifuged again under the same conditions. ROS fluorescence was measured using an Attune NxT flow cytometer (Thermo Fisher Scientific, USA) with an excitation wavelength of 488 nm and emission detection at 525 nm.

### Tetramethylrhodamine ethyl ester perchlorate (TMRE) staining

Mitochondrial membrane potential (MMP) was assessed using tetramethylrhodamine ethyl ester perchlorate (C2001S, Beyotime, China). HNEpCs were seeded in the 6-well plates at a density of 1 × 10^6^ cells/well. After washing with PBS, cells were incubated with 1 mL of TMRE working solution (final concentration 200 nM in pre-warmed, serum-free RPMI 1640 medium) at 37 °C for 30 min. The staining solution was then removed, and the cells were washed twice with pre-warmed culture medium. Finally, 2 mL of fresh complete medium was added, and fluorescence was observed under a fluorescence microscope. Images were acquired on an inverted fluorescence microscope equipped with a 40 × objective lens. For each field, images of 1,024 × 1,024 pixels were collected using identical exposure time and gain settings across all experimental groups. For TMRE staining, three independent biological replicates were performed, and at least three randomly selected, non-overlapping fields per well were imaged as technical replicates.

### Co-Immunoprecipitation (Co-IP)

HNEpCs were seeded into 6-well plates at a density of 2 × 10^6^ cells/well. After two washes with PBS, cells were lysed in 500 μL of RIPA buffer supplemented with PMSF (protease inhibitor) on ice. Lysates were centrifuged at 12,000 rpm at 4 °C for 15 min, and the supernatant was collected. Protein concentration was determined using the bicinchoninic acid (BCA) assay.

For pre-clearing, 5 μL each of Protein A and Protein G agarose beads were added to the cell lysates and incubated at 4 °C for 30–60 min with gentle rotation. After centrifugation at 12,000 rpm for 1 min, the supernatant was transferred to a new tube and incubated overnight at 4 °C with 2 μg of target-specific primary antibody or 1 μg of isotype control antibody (negative control). The following day, 5 μL each of Protein A and Protein G agarose beads were added, and the mixture was incubated again at 4°C overnight. The immunoprecipitates were collected by centrifugation at 12,000 rpm for 1 min and washed five times with cold wash buffer. The final pellet was re-suspended in SDS-PAGE loading buffer, vortexed, briefly centrifuged, and boiled at 100 °C for 5 min. After centrifugation at 14,000 rpm for 1 min, the supernatant was collected and subjected to Western blot analysis.

### Statistical analysis

Data were analyzed and plotted using GraphPad Prism 9 (Version 9.4.0), and figures were organized using Adobe Illustrator 2023. Data are expressed as mean ± standard deviation (SD). All experiments were performed with at least three independent biological replicates unless otherwise stated. Normality of data distribution was assessed using the Shapiro–Wilk test. For two-group comparisons, unpaired Student’s t-tests were used. For comparisons among more than two groups, one-way ANOVA followed by Tukey’s post hoc test was applied to account for multiple comparisons. Statistical significance was determined at a threshold of P < 0.05.

## Results

### Identification of genes related to AR and mitochondrial autophagy

**Fig 1:**
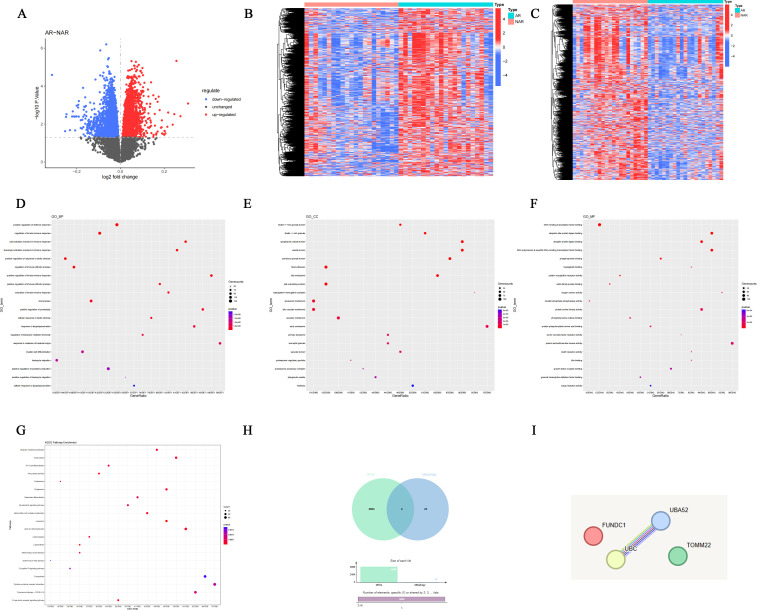
Identification of genes related to allergic rhinitis (AR) and mitochondrial autophagy. **(A-C)** Volcano plot and heatmap showing differentially expressed genes (DEGs) between AR and control samples. **(D-G)** GO and KEGG enrichment results visualized as bubble plots. **(H)** Venn diagram showing the overlap between AR-related DEGs and mitophagy-related genes. **(I)** Protein–protein interaction (PPI) network of intersecting genes constructed using the STRING database.

The dataset of GSE50223 was obtained from the GEO database and contained 42 samples, including 21 AR samples and 21 controls. A total of 4,988 DEGs were identified, including 2,548 up-regulated and 2,440 down-regulated genes. Volcano maps and heatmaps were generated to visualize the distribution of DEGs (Figs 1A-C). To explore the biological relevance of these genes, GO and KEGG pathway enrichment analysis were performed, and the results were presented as bubble plots (Figs 1D-G). To identify genes potentially involved in mitophagy, we intersected the 4988 DEGs with 29 mitophagy-related genes obtained from PathCards, resulting in four overlapping genes: TOMM22, FUNDC1, UBA52, and UBC. These four candidates were further analyzed using STRING for PPI analysis, with the confidence score set at 0.400. A Venn diagram and PPI network were constructed (Figs 1H and 1I). The results suggested that UBC and UBA52 may be key genes linking AR and mitochondrial autophagy.

### UBC and UBA52 were upregulated, while PINK1 and Parkin are downregulated in AR cell models

**Fig 2:**
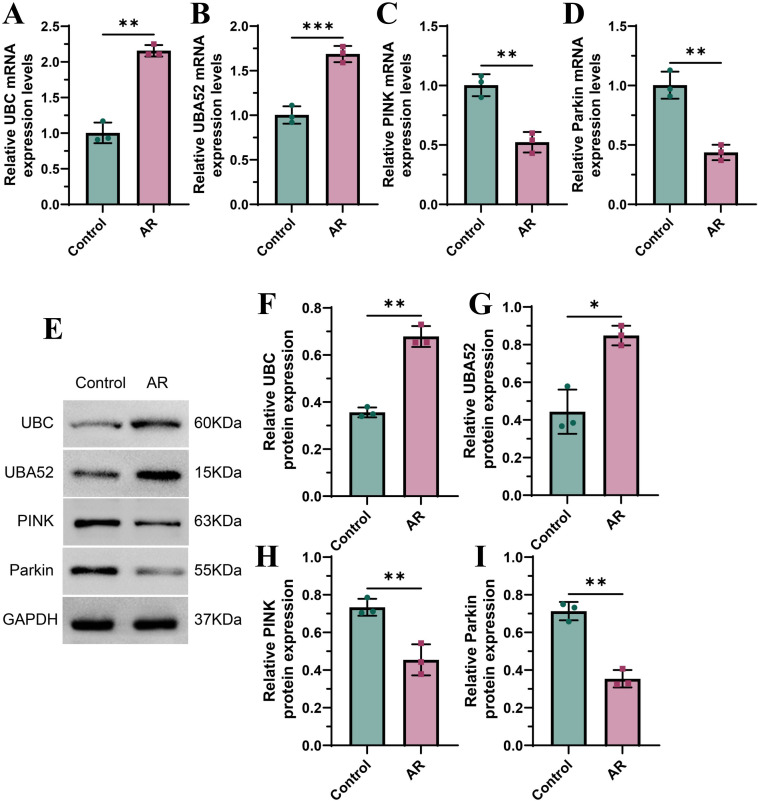
UBC and UBA52 were up-regulated in the AR model. **(A-D)** Relative mRNA expression levels of UBC **(A)**, UBA52 **(B)**, PINK1 **(C)**, and Parkin (D) in AR and control groups measured by RT-qPCR. (E-I) Western blot analysis showing protein levels of UBC, UBA52, PINK1, and Parkin. Data are shown as mean ± SD (n = 3). RT-qPCR results include technical triplicates. *P < 0.05, **P < 0.01, ***P < 0.001.

To investigate the role of UBC and UBA52 in AR, we established an in vitro AR model by treating HNEpCs with IL-13. RT-qPCR analysis showed that UBC and UBA52 mRNA levels were significantly upregulated in the AR group compared to controls. In contrast, the expression of mitophagy-related genes PINK1 and Parkin was markedly downregulated (Figs 2A-D). Western blot analysis confirmed these transcriptional changes at the protein level: UBC and UBA52 protein levels were elevated, while PINK1 and Parkin protein levels were reduced in AR cells (Figs 2E-I). These results indicate that AR is associated with upregulation of UBC/UBA52 and concurrent inhibition of PINK1–Parkin–mediated mitophagy.

### Mitochondrial dysfunction and potential mitophagy impairment in AR cell models

**Fig 3:**
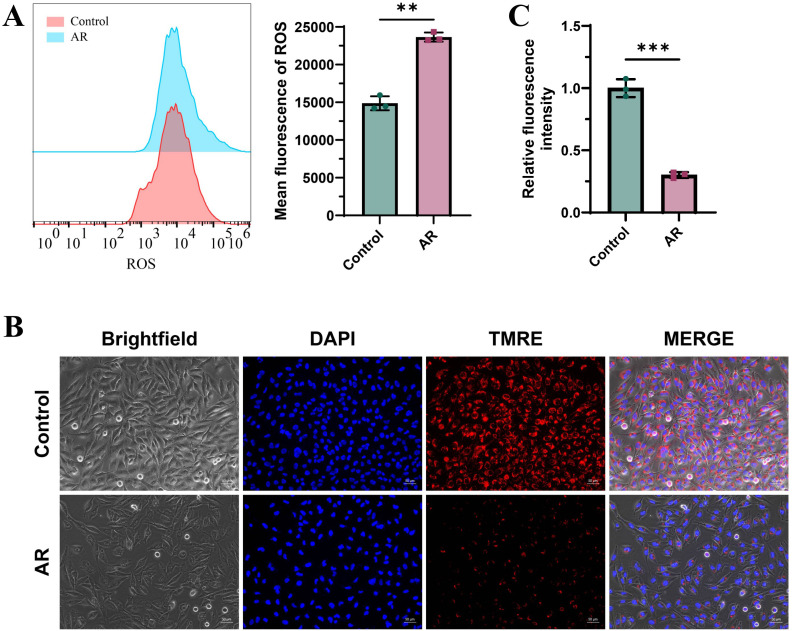
ROS increased and membrane potential decreased in AR cell models. **(A)** Intracellular ROS levels measured by flow cytometry in AR and control groups. **(B-C)** Representative fluorescence microscopy images showing MMP changes assessed by TMRE staining. Blue fluorescent represents DAPI. Red fluorescent represents MMP. Scale bar = 50 μm. Data are shown as mean ± SD (n = 3). **P < 0.01, ***P < 0.001.

To assess mitochondrial dysfunction in AR, we measured the ROS levels and MMP in IL-13–induced HNEpC cells. Flow cytometry analysis showed significantly increased ROS levels in the AR group compared with the control group (Fig 3A). TMRE staining revealed a marked reduction in MMP in the AR group (Figs 3B-C). These findings indicate mitochondrial dysfunction under AR conditions, including increased ROS levels and reduced MMP, which may be associated with impaired mitophagy.

### Silencing UBC restores PINK1–Parkin expression and mitochondrial autophagy in AR models

**Fig 4:**
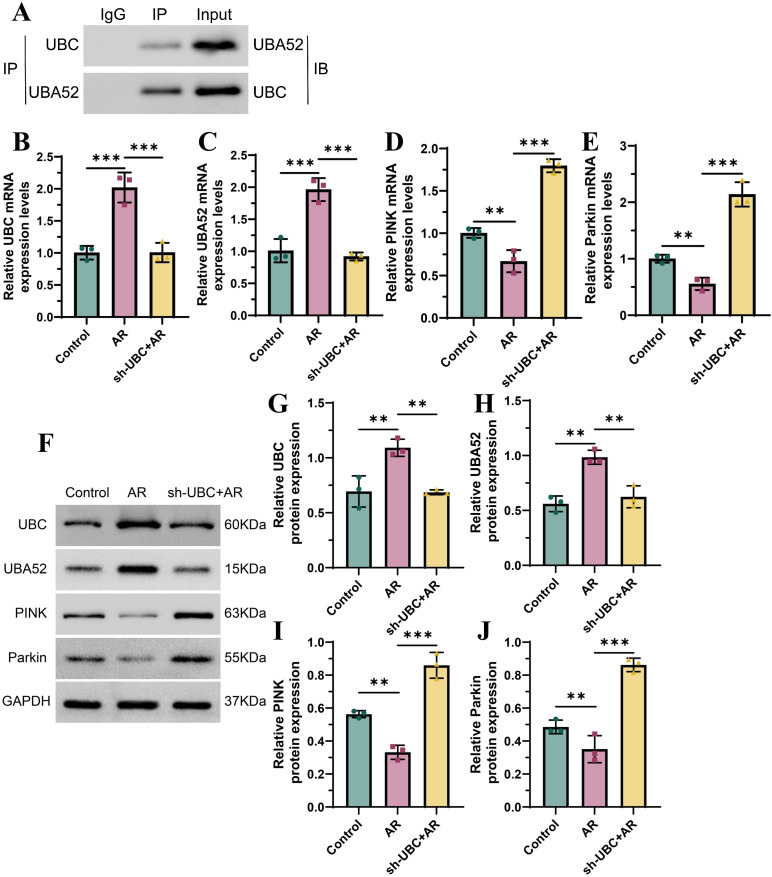
UBC down-regulated UBA52 to activate mitochondrial autophagy in AR models. **(A)** Co-immunoprecipitation showing interaction between UBC and UBA52. **(B-E)** Relative mRNA expression levels of UBC, UBA52, PINK1, and Parkin after UBC knockdown; **(B)** One-way ANOVA: F(2, 6) = 34.7, *P* < 0.001; **(C)** One-way ANOVA: F(2, 6) = 43.3, *P* < 0.001. **(D)** One-way ANOVA: F(2, 6) = 114, *P* < 0.001. **(E)** One-way ANOVA: F(2, 6) = 93.9, *P* < 0.001. **(F-J)** Western blot analysis of protein levels for UBC, UBA52, PINK1, and Parkin; **(G)** One-way ANOVA: F(2, 6) = 18.1, *P* = 0.003; **(H)** One-way ANOVA: F(2, 6) = 24.5, *P* = 0.001. **(I)** One-way ANOVA: F(2, 6) = 75.1, *P* < 0.001. **(J)** One-way ANOVA: F(2, 6) = 62.6, *P* < 0.001. Data are shown as mean ± SD (n = 3). **P < 0.01, ***P < 0.001.

To further explore the regulatory relationship between UBC, UBA52, and mitophagy, we performed CO-IP experiments, which confirmed a protein–protein interaction between UBC and UBA52 (Fig 4A). Lentiviral knockdown of UBC in the AR model was successfully achieved (Fig 4B). Upon UBC silencing, RT-qPCR analysis revealed a significant decrease in UBA52 expression, accompanied by restoration of PINK1 and Parkin expression (Figs 4C-E). The results of Western blot were consistent with those of RT-qPCR (Figs 4F-J). These results suggest that silencing UBC downregulated UBA52 expression and restored PINK1 and Parkin levels, indicating a potential involvement of the PINK1–Parkin signaling pathway in mitophagy.

### Silencing UBC protected mitochondrial damage in the AR model

**Fig 5:**
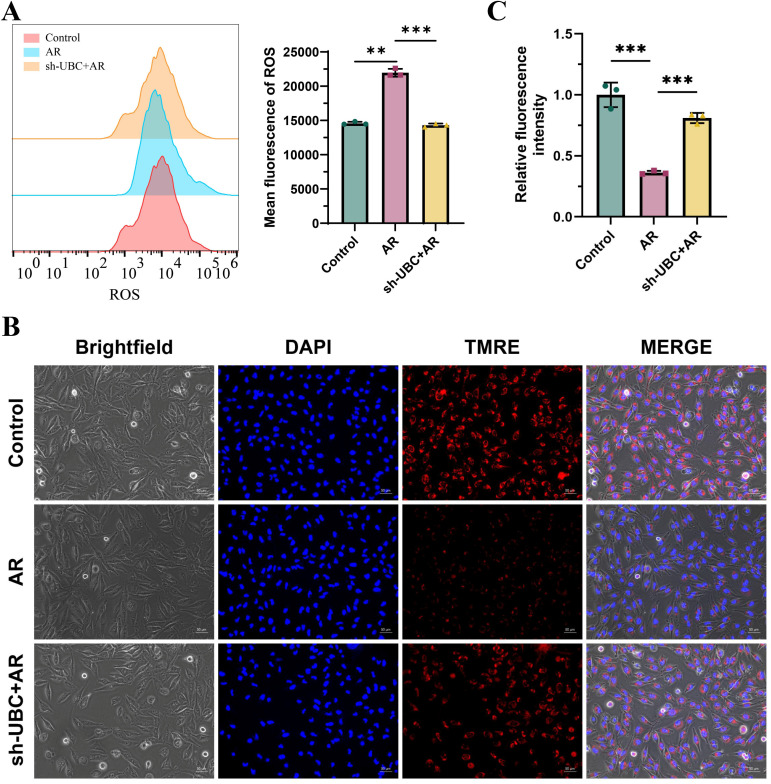
Silencing UBC protected mitochondrial damage in the AR model. **(A)** ROS levels detected by flow cytometry in control, AR, and UBC-silenced AR groups. One-way ANOVA: F(2, 6) = 388, *P* < 0.001. **(B)** Mitochondrial membrane potential (MMP) assessed by TMRE staining. Blue: DAPI. Red: MMP. Scale bar = 50 μm. **(C)** Quantification of TMRE fluorescence intensity. One-way ANOVA: F(2, 6) = 53.6, *P* < 0.001. Data are shown as mean ± SD (n = 3). **P < 0.01, ***P < 0.001.

To evaluate the‌‌ impact of silencing UBC on mitochondrial function and mitophagy-related processes, intracellular ROS levels were measured by flow cytometry. Compared with the AR group, UBC knockdown significantly reduced ROS accumulation in HNEpCs (Fig 5A). The fluorescence intensity of TMRE, which reflects MMP levels, was markedly increased in the UBC-silenced group compared to the AR group, indicating improved mitochondrial function (Figs 5B-C). These results suggest that UBC silencing may be associated with enhanced mitophagy-related activity and improved mitochondrial quality control in AR.

## Discussions

In this study, bioinforma‌‌tics analysis identified UBC and UBA52 as genes potentially involved in AR. Both were upregulated in AR cell models, while mitophagy-related genes PINK1 and Parkin were down-regulated, suggesting impaired mitochondrial quality control. Functional assays showed that silencing UBC reduced UBA52 expression and was associated with activation of the PINK1-Parkin pathway. These findings suggest that UBC may be involved in the regulation of mitophagy in AR, and highlight the potential of targeting UBC to restore mitochondrial homeostasis in allergic inflammation.

Autophagy prevents excessive inflammation by eliminating microorganisms and endogenous stimuli. When autophagy clearance fails, inflammation ensues as the body responds to persistent danger. Autophagy has attracted increasing attention in recent years as a key mechanism involved in cellular stress response and homeostasis. [8,9]. Studies have shown that rs12212740 gene locus of autophagy related gene ATG5 is involved in the main pathogenesis of asthma [10], and the high expression of autophagy genes ATG5, ATG7 and ATG8 play an important role in the pathogenesis of atopic dermatitis [11]. Given the growing evidence supporting the role of autophagy in allergic inflammation, this study focused on the role of mitochondrial autophagy in the pathogenesis of AR, providing a new therapeutic target for AR.

Mitochondrial kinases PINK1 and Parkin are the two most important proteins involved in mitochondrial autophagy. In healthy organisms, PINK1 is rapidly degraded, so the amount is very low [12]. Under stress, the input of PINK1 in the mitochondrial outer membrane complex is blocked, resulting in activation of its ubiquitin kinase activity through autophosphorylation and initiating Parkin-dependent mitochondrial clearance. Ub phosphorylated by PINK1 binds to the RING1 domain of Parkin and induces phosphorylation of Parkin. Upon activation, Parkin translocates from the cytoplasm to mitochondria [4]. Cao et al. showed that curcumin improves intestinal barrier mitochondrial damage by activating Parkin-dependent mitochondrial autophagy through AMPK-TFEB signaling pathway [13]. Li et al. believed that activation of PINK1/Parkin-mediated mitochondrial autophagy could reduce mitochondrial apoptosis and thus accelerate pulmonary vascular remodeling [14]. Lin and colleagues demonstrated that mitochondrial autophagy induced by the PINK1-Parkin pathway inhibits mitochondrial ROS and NLRP3 inflammasome to alleviate contrast media-induced acute kidney injury [15]. In addition, Liu et al. found that Polydatin can inhibit mitochondrial damage in AR by promoting mitochondrial autophagy mediated by PINK1-parkin [6]. In our study, expression of PINK1 and Parkin was significantly downregulated in AR cells, accompanied by increased ROS levels and reduced MMP, suggesting suppression of mitophagy and mitochondrial damage under allergic inflammation. However, GO and KEGG enrichment analyses did not reveal significant mitochondrial-related pathways; instead, lysosome-associated terms were enriched, which may reflect activation of the general autophagy–lysosomal degradation system rather than mitophagy specifically. Given that lysosomes are central components shared by multiple forms of autophagy, lysosome enrichment alone does not necessarily indicate selective mitochondrial clearance [9,16]. Therefore, this finding should be interpreted with caution and in conjunction with molecular evidence of PINK1–Parkin signaling. This discrepancy may indicate early or incomplete mitophagy, involvement of non-canonical pathways, or post-transcriptional regulation of mitochondrial quality control. Further studies will be needed to clarify these mechanisms in the context of AR.

UBC and UBA52 are involved in the process of ubiquitination, a key protein modification mechanism that marks proteins for degradation or other fates [17]. Studies have shown that both ubiquitination and deubiquitination enzymes are involved in mitochondrial autophagy mediated by PINK1/Parkin. There are some negative regulatory mechanisms related to the ubiquitin proteasome system in the PINK1/Parkin pathway, which maintain the balance of mitochondrial autophagy and prevent mitochondrial damage caused by excessive mitochondrial autophagy [18]. Through the analysis of multiple transcriptome data, Jiang et al. found that UBC and UBA52 may be the main factors driving the changes of mitochondrial autophagy function in renal carcinoma clear cells [19]. In this study, through GEO transcriptome analysis of publicly available GEO datasets, we identified UBC and UBA52 as potential key genes associated with AR and mitochondrial regulation. Their upregulation in AR cell models was confirmed by RT-qPCR and Western blot. CO-IP assays further indicated a protein–protein interaction between UBC and UBA52. Functionally, silencing UBC led to decreased UBA52 expression and was associated with reversal of PINK1 and Parkin downregulation, improved MMP, and reduced ROS levels. These results suggest that UBC may be associated with the regulation of mitophagy via the UBA52/PINK1–Parkin axis.

However, these findings are based on *in vitro* experiments, and we acknowledge that the molecular mechanism remains incompletely understood. In addition, the lack of validation using clinical samples limits the direct translational applicability of our findings. Future *in vivo* studies are needed to validate the role of UBC and UBA52 in modulating mitophagy and mitochondrial function in the context of AR. Nevertheless, the identification of the UBC–UBA52–PINK1/Parkin axis may provide a potential therapeutic target for restoring mitochondrial homeostasis and alleviating inflammatory responses in AR.

## Conclusions

In conclusion, this study identifies UBC and UBA52 as potential regulators of mitophagy in AR. UBC silencing may contribute to the restoration of mitochondrial quality control potentially through the PINK1–Parkin pathway, providing a potential therapeutic target for AR.

## Supporting information

S1 File The original bands of protein Western blotting in the article. (ZIP)

S2 File WB. (PDF)

## References

[1] Bousquet J, Anto JM, Bachert C, Baiardini I, Bosnic-Anticevich S, Walter Canonica G, et al. Allergic rhinitis. Nat Rev Dis Primers. 2020;6(1):95. doi: 10.1038/s41572-020-00227-0 33273461

[2] Siti Sarah CO, Mohd Ashari NS. Exploration of allergic rhinitis: epidemiology, predisposing factors, clinical manifestations, laboratory characteristics, and emerging pathogenic mechanisms. Cureus. 2024;16(10):e71409. 10.7759/cureus.71409 PMC11558229 39539885

[3] Zhang Y, Zhang L. Increasing prevalence of allergic rhinitis in China. Allergy Asthma Immunol Res. 2019;11(2):156–69. 30661309 10.4168/aair.2019.11.2.156 PMC6340797

[4] Durcan TM, Fon EA. The three ’P’s of mitophagy: PARKIN, PINK1, and post-translational modifications. Genes Dev. 2015;29(10):989–99. doi: 10.1101/gad.262758.115 25995186 PMC4441056

[5] Xu Y, Tang Y, Lu J, Zhang W, Zhu Y, Zhang S, et al. PINK1-mediated mitophagy protects against hepatic ischemia/reperfusion injury by restraining NLRP3 inflammasome activation. Free Radic Biol Med. 2020;160:871–86. doi: 10.1016/j.freeradbiomed.2020.09.015 32947010

[6] Liu S, Wang C, Zhang Y, Zhang Y, Song Y, Jiang J, et al. Polydatin inhibits mitochondrial damage and mitochondrial ROS by promoting PINK1-Parkin-mediated mitophagy in allergic rhinitis. FASEB J. 2023;37(4):e22852. doi: 10.1096/fj.202201231RR 36906289

[7] Wang C, Zhuo J-J, Li W-Q, Zhou M-L, Cheng K-J. Role of autophagy and mitophagy of group 2 innate lymphoid cells in allergic and local allergic rhinitis. World Allergy Organ J. 2024;17(2):100852. doi: 10.1016/j.waojou.2023.100852 38298830 PMC10827603

[8] Wallace DV, Dykewicz MS, Bernstein DI, Blessing-Moore J, Cox L, Khan DA, et al. The diagnosis and management of rhinitis: an updated practice parameter. J Allergy Clin Immunol. 2008;122(2 Suppl):S1-84. doi: 10.1016/j.jaci.2008.06.003 18662584

[9] Li W, He P, Huang Y, Li Y-F, Lu J, Li M, et al. Selective autophagy of intracellular organelles: recent research advances. Theranostics. 2021;11(1):222–56. doi: 10.7150/thno.49860 33391472 PMC7681076

[10] Zhao H, Dong F, Li Y, Ren X, Xia Z, Wang Y, et al. Inhibiting ATG5 mediated autophagy to regulate endoplasmic reticulum stress and CD4+ T lymphocyte differentiation: Mechanisms of acupuncture’s effects on asthma. Biomed Pharmacother. 2021;142:112045. doi: 10.1016/j.biopha.2021.112045 34426257

[11] Hailfinger S, Schulze-Osthoff K. Impaired Autophagy in Psoriasis and Atopic Dermatitis: A New Therapeutic Target*?* J Invest Dermatol. 2021;141(12): p. 2775–7. 34565564 10.1016/j.jid.2021.06.006

[12] Lazarou M, Sliter DA, Kane LA, Sarraf SA, Wang C, Burman JL, et al. The ubiquitin kinase PINK1 recruits autophagy receptors to induce mitophagy. Nature. 2015;524(7565):309–14. doi: 10.1038/nature14893 26266977 PMC5018156

[13] Cao S, Wang C, Yan J, Li X, Wen J, Hu C. Curcumin ameliorates oxidative stress-induced intestinal barrier injury and mitochondrial damage by promoting Parkin dependent mitophagy through AMPK-TFEB signal pathway. Free Radic Biol Med. 2020;147:8–22. doi: 10.1016/j.freeradbiomed.2019.12.004 31816386

[14] Linqing L, Yuhan Q, Erfei L, Yong Q, Dong W, Chengchun T, et al. Hypoxia-induced PINK1/Parkin-mediated mitophagy promotes pulmonary vascular remodeling. Biochem Biophys Res Commun. 2021;534:568–75. doi: 10.1016/j.bbrc.2020.11.040 33239167

[15] Lin Q, Li S, Jiang N, Shao X, Zhang M, Jin H, et al. PINK1-parkin pathway of mitophagy protects against contrast-induced acute kidney injury via decreasing mitochondrial ROS and NLRP3 inflammasome activation. Redox Biol. 2019;26:101254. doi: 10.1016/j.redox.2019.101254 31229841 PMC6597739

[16] Levine B, Kroemer G. Biological functions of autophagy genes: a disease perspective. Cell. 2019;176(1–2):11–42. 30633901 10.1016/j.cell.2018.09.048 PMC6347410

[17] Deng L, Meng T, Chen L, Wei W, Wang P. The role of ubiquitination in tumorigenesis and targeted drug discovery. Signal Transduct Target Ther. 2020;5(1):11. doi: 10.1038/s41392-020-0107-0 32296023 PMC7048745

[18] Oshima Y, Verhoeven N, Cartier E, Karbowski M. The OMM-severed and IMM-ubiquitinated mitochondria are intermediates of mitochondrial proteotoxicity-induced autophagy in PRKN/parkin-deficient cells. Autophagy. 2021;17(11):3884–6. doi: 10.1080/15548627.2021.1964887 34486484 PMC8632337

[19] Jiang L, Ren X, Yang J, Chen H, Zhang S, Zhou X, et al. Mitophagy and clear cell renal cell carcinoma: insights from single-cell and spatial transcriptomics analysis. Front Immunol. 2024;15:1400431. doi: 10.3389/fimmu.2024.1400431 38994370 PMC11236570

