# Supplementary figures and images for: UBC/UBA52 silencing restores PINK1-Parkin-mediated mitochondrial autophagy in allergic rhinitis
Source: PLoS One. 2026 Jun 10;21(6):e0350815. doi: 10.1371/journal.pone.0350815 (PMC13252774; doi:10.1371/journal.pone.0350815)

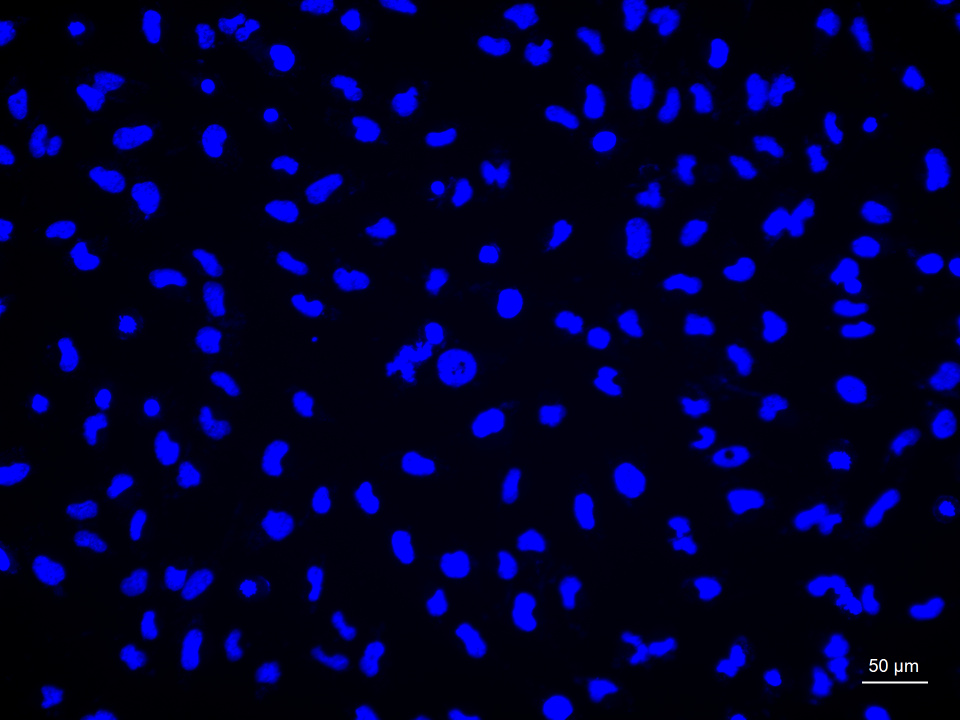

Supplement: S1 File — (ZIP) [file pone.0350815.s001.zip › Figure 5B/sh-UBC+AR/DAPI-1.tiff]

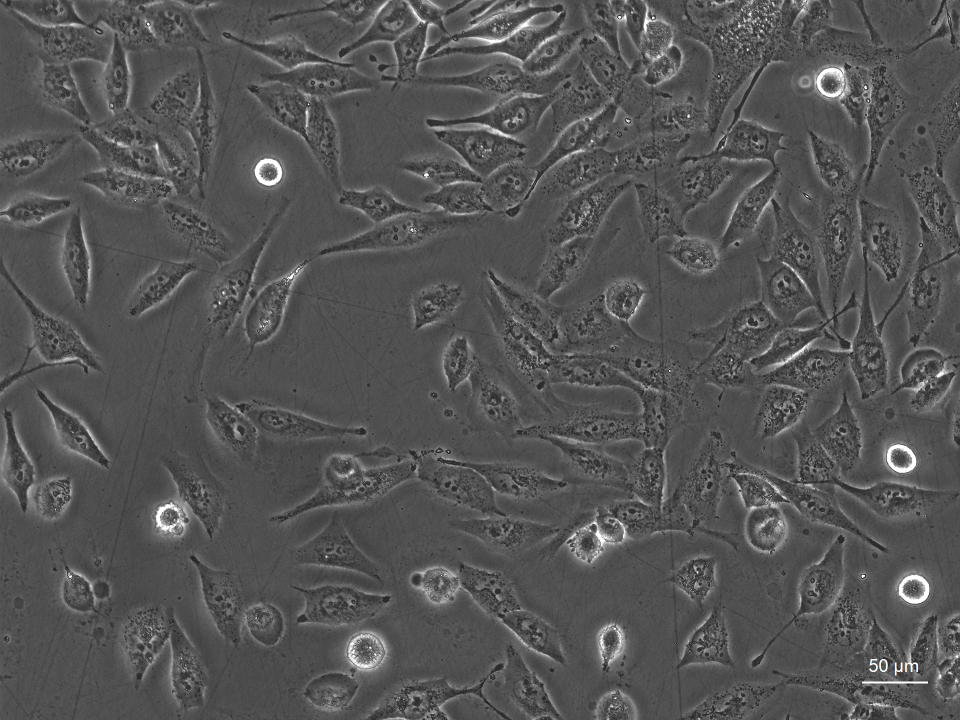

Supplement: S1 File — (ZIP) [file pone.0350815.s001.zip › Figure 5B/sh-UBC+AR/Brightfield-3.tiff]

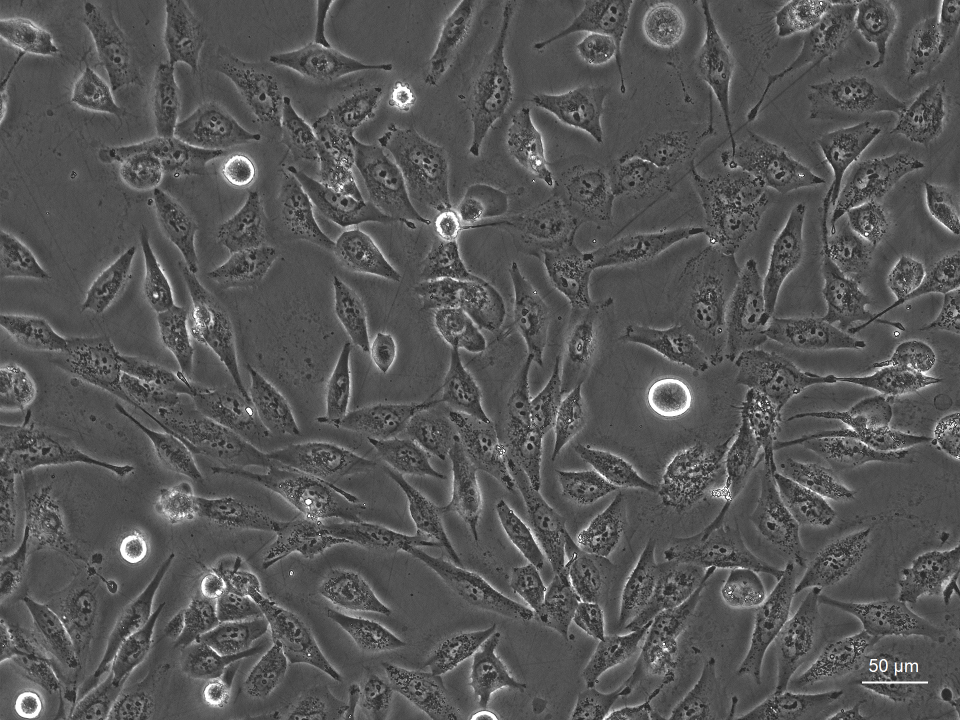

Supplement: S1 File — (ZIP) [file pone.0350815.s001.zip › Figure 5B/sh-UBC+AR/Brightfield-2.tiff]

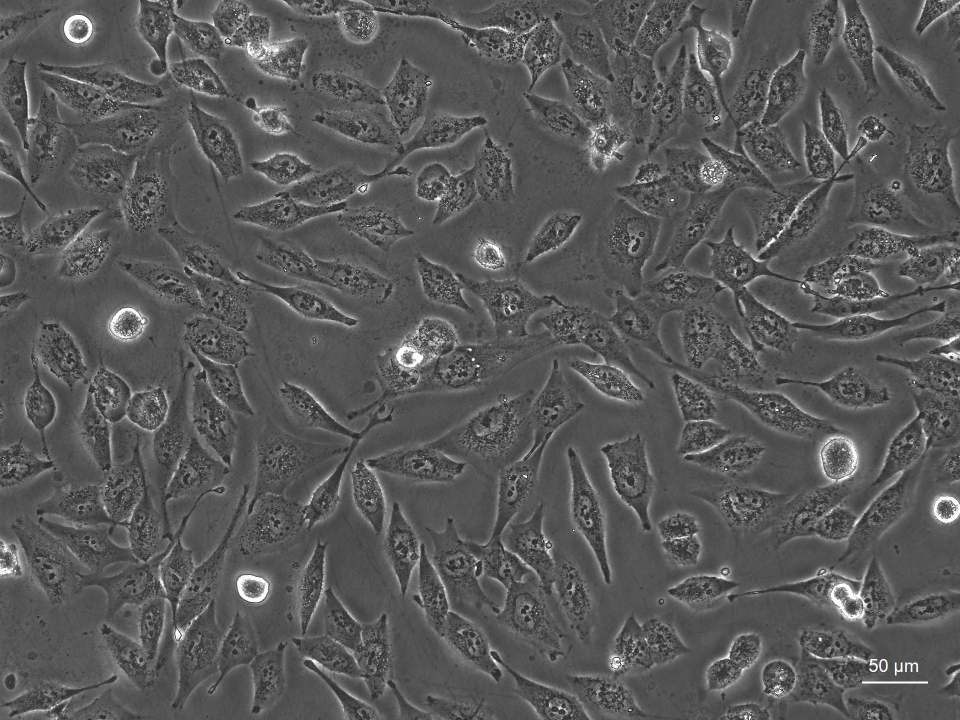

Supplement: S1 File — (ZIP) [file pone.0350815.s001.zip › Figure 5B/sh-UBC+AR/Brightfield-1.tiff]

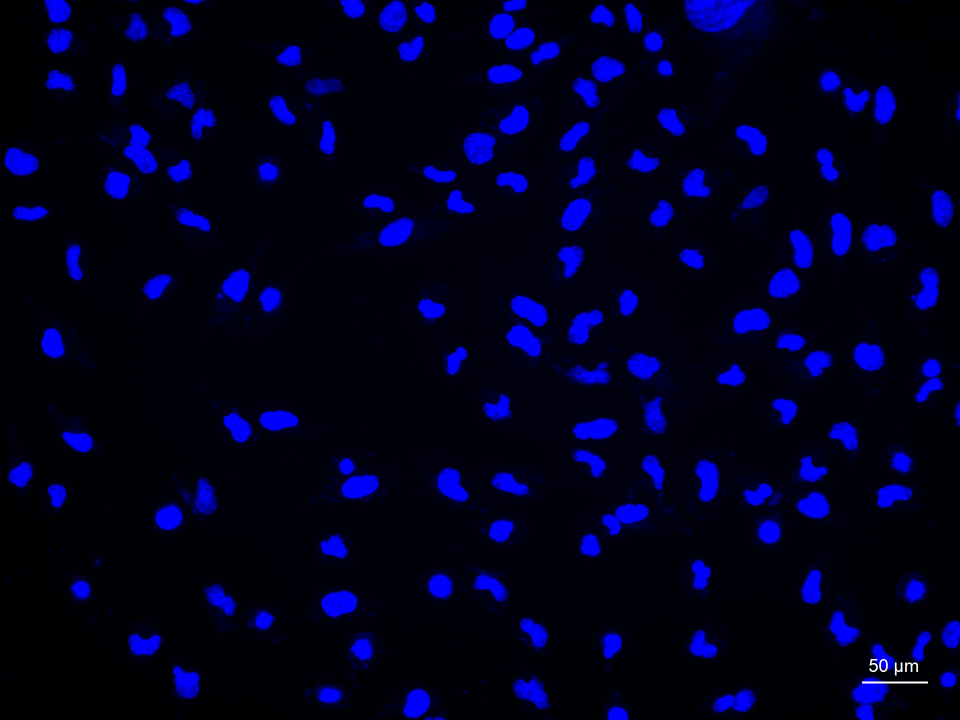

Supplement: S1 File — (ZIP) [file pone.0350815.s001.zip › Figure 5B/sh-UBC+AR/DAPI-3.tiff]

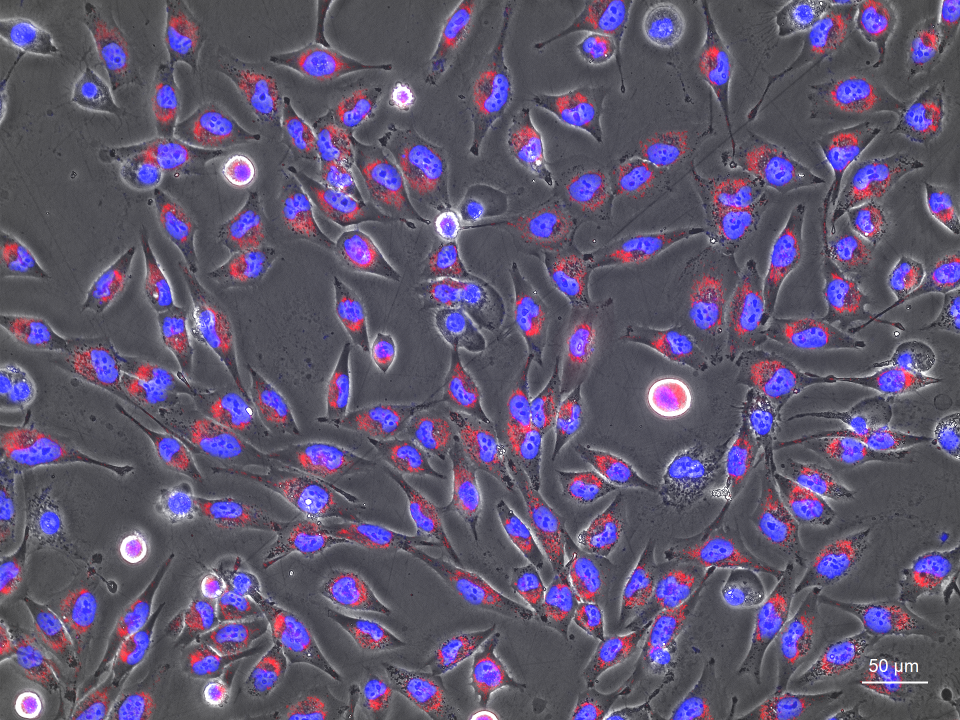

Supplement: S1 File — (ZIP) [file pone.0350815.s001.zip › Figure 5B/sh-UBC+AR/MERGE-2.tiff]

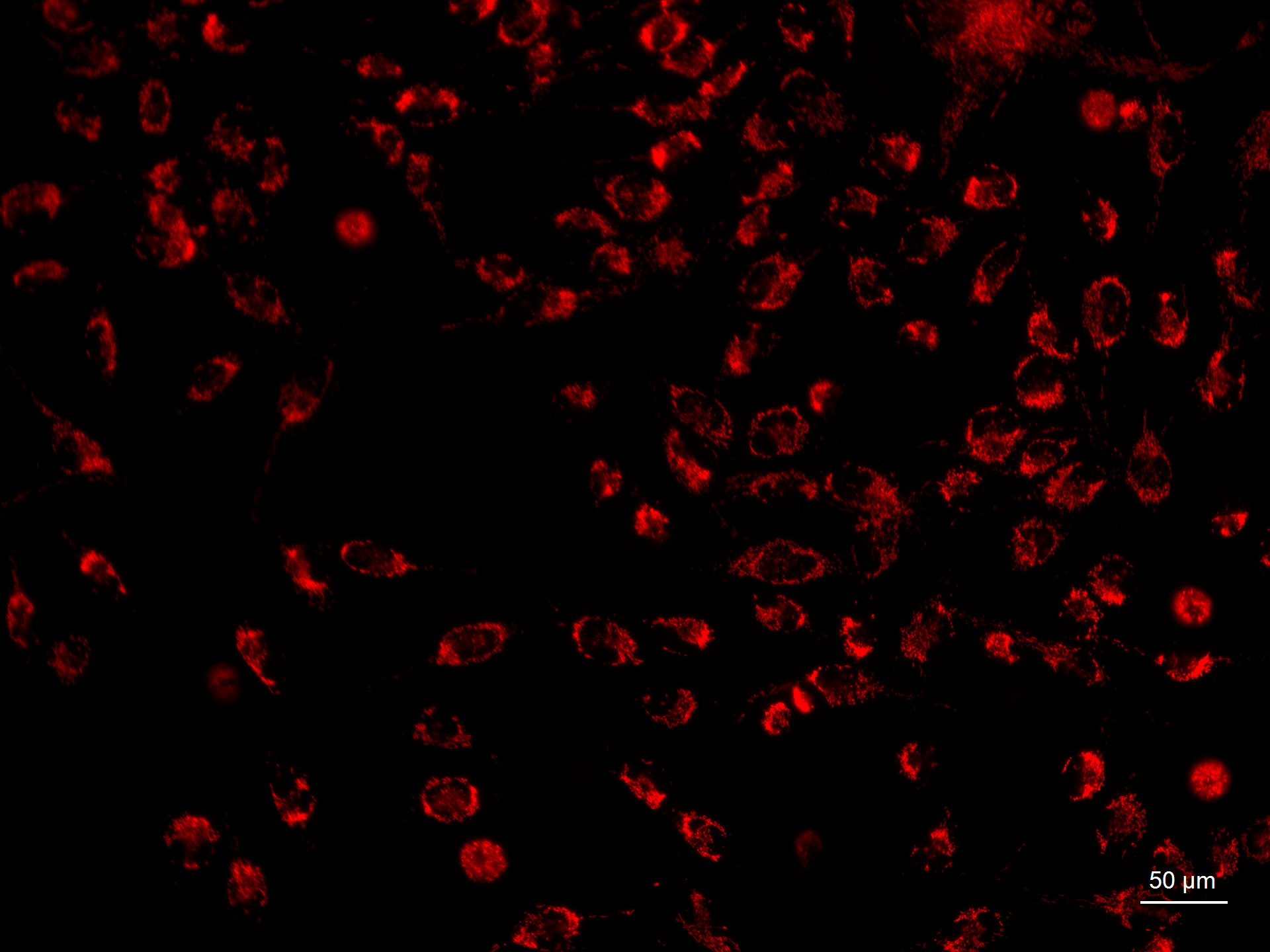

Supplement: S1 File — (ZIP) [file pone.0350815.s001.zip › Figure 5B/sh-UBC+AR/TMRE-3.tif]

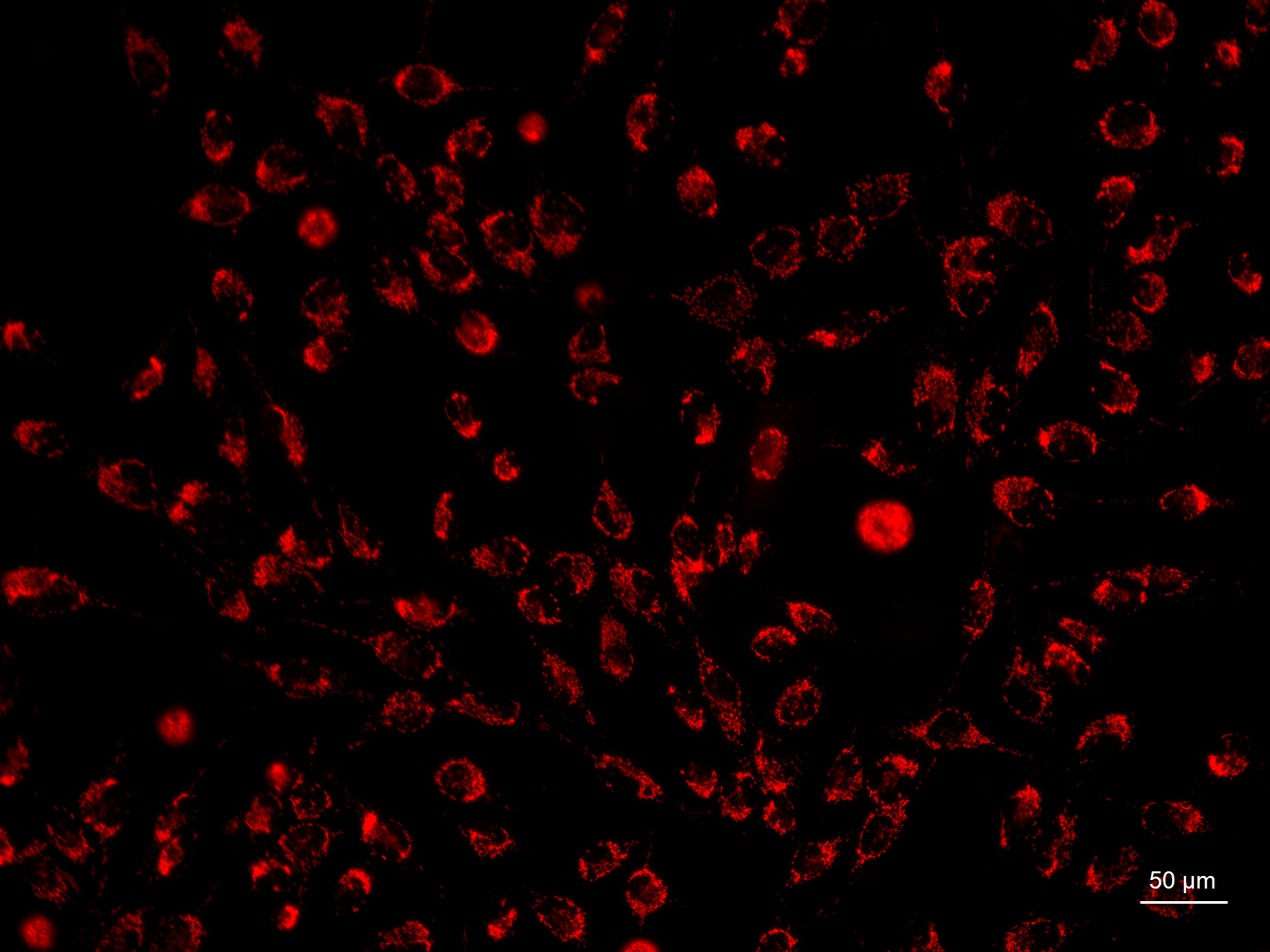

Supplement: S1 File — (ZIP) [file pone.0350815.s001.zip › Figure 5B/sh-UBC+AR/TMRE-2.tif]

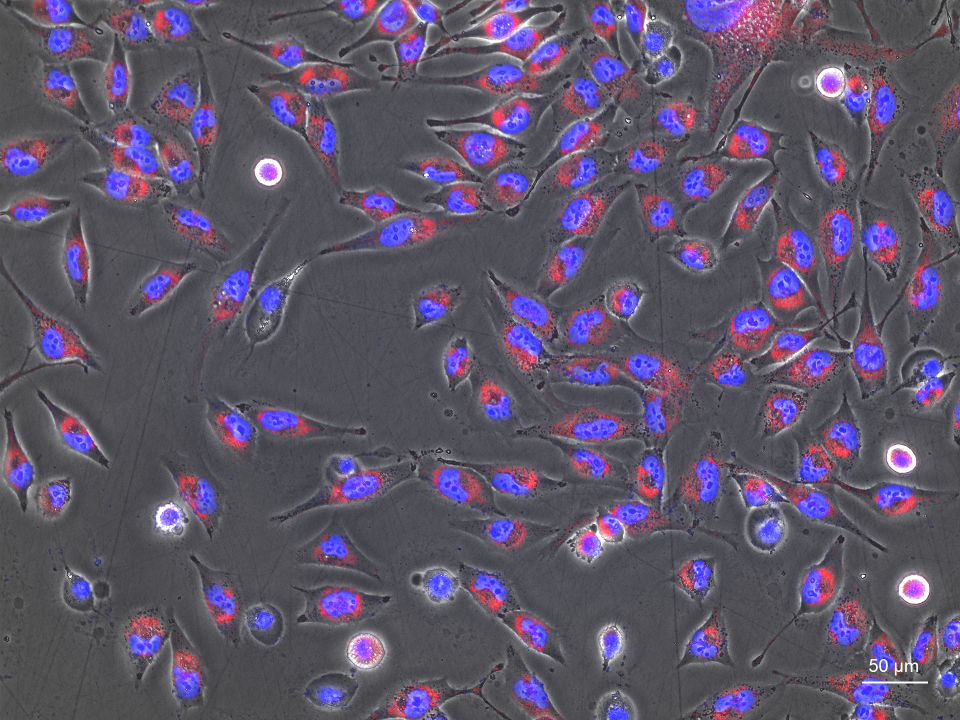

Supplement: S1 File — (ZIP) [file pone.0350815.s001.zip › Figure 5B/sh-UBC+AR/MERGE-3.tiff]

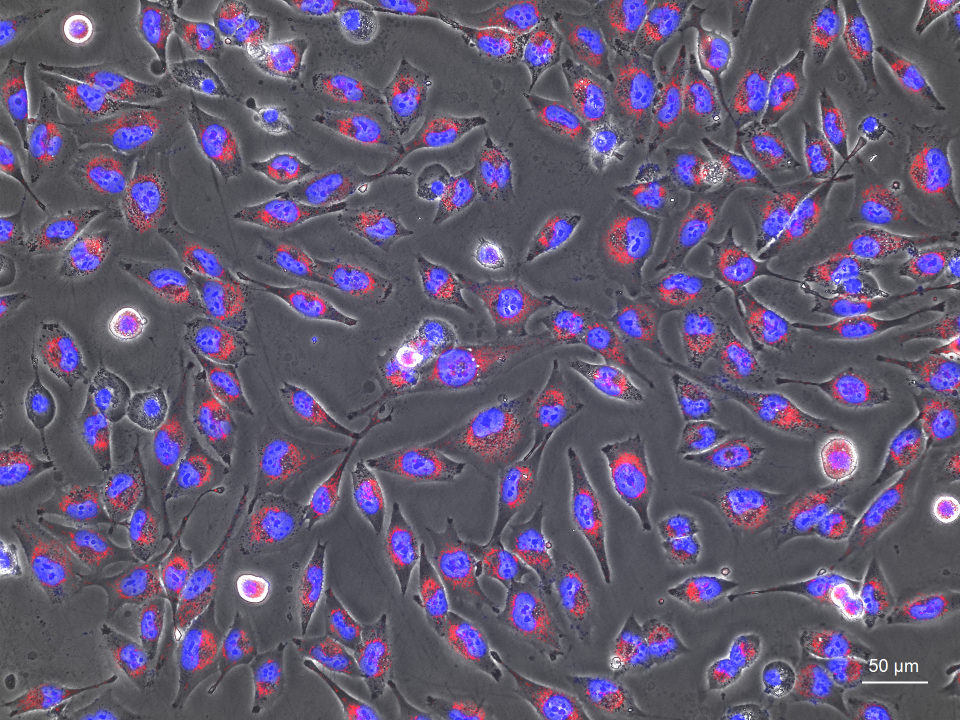

Supplement: S1 File — (ZIP) [file pone.0350815.s001.zip › Figure 5B/sh-UBC+AR/MREGE-1.tiff]

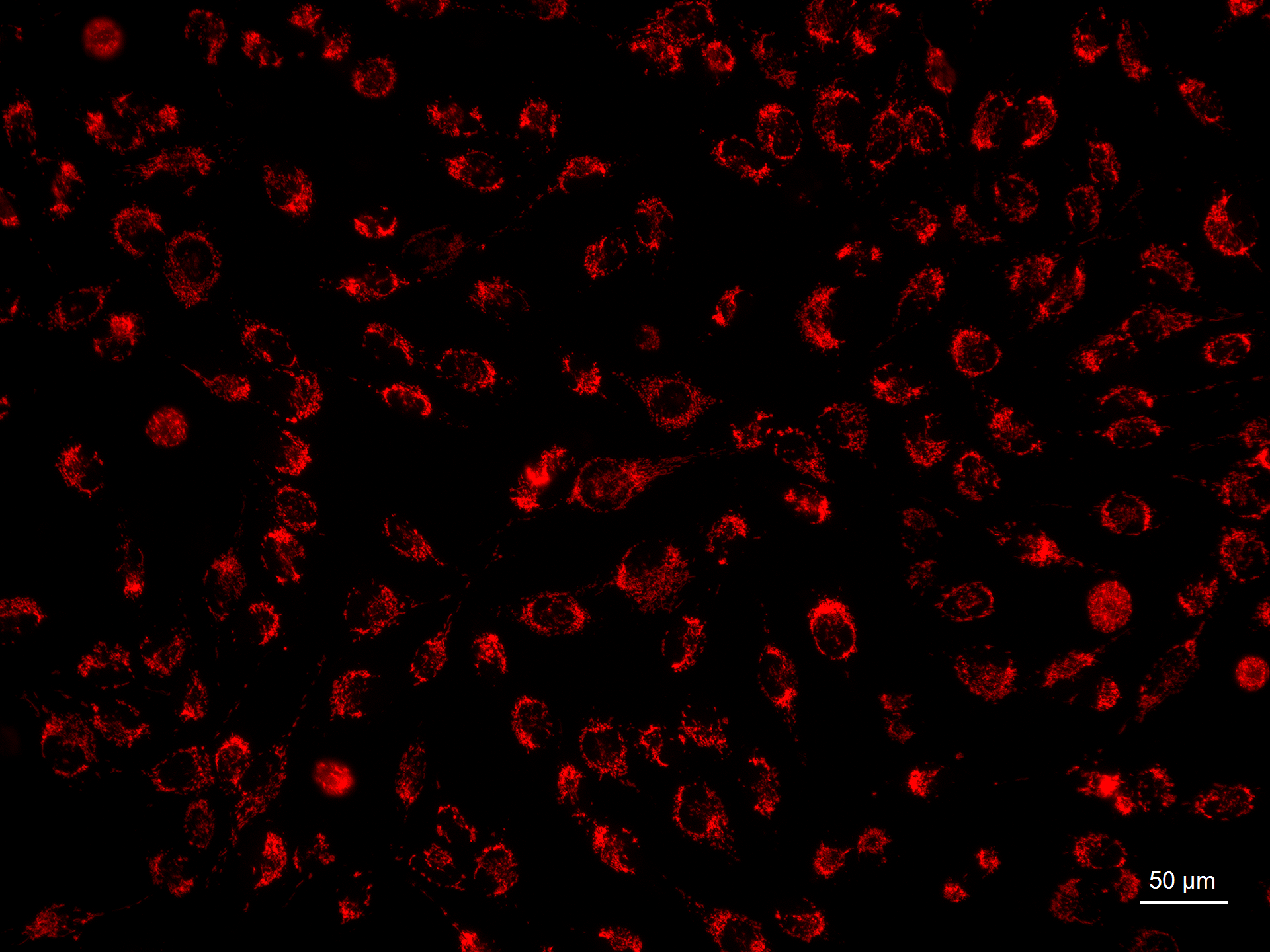

Supplement: S1 File — (ZIP) [file pone.0350815.s001.zip › Figure 5B/sh-UBC+AR/TMRE-1.tif]

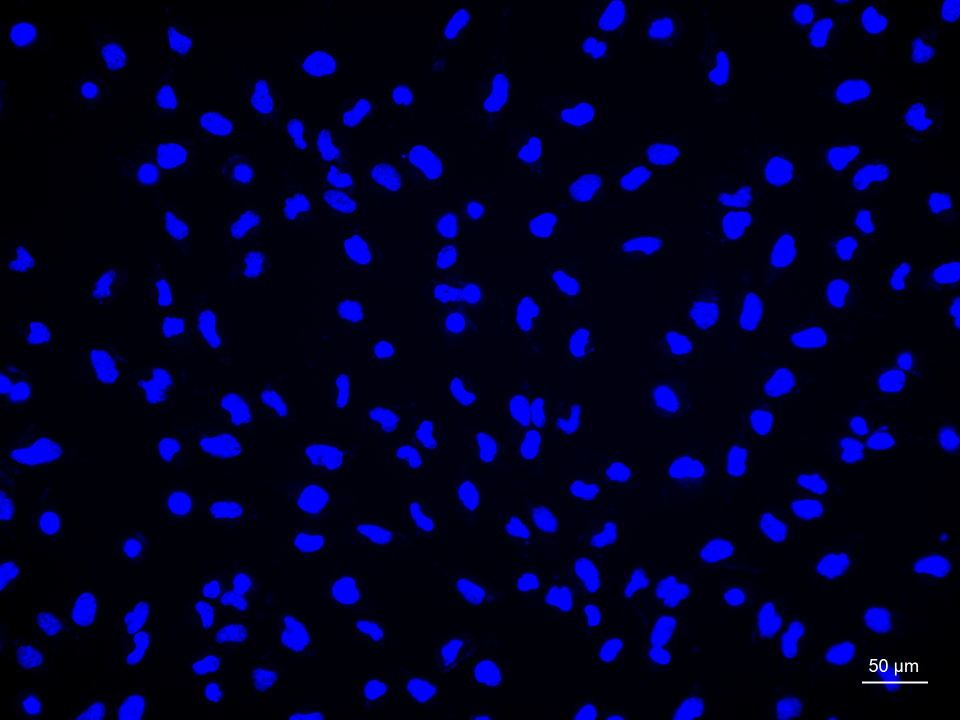

Supplement: S1 File — (ZIP) [file pone.0350815.s001.zip › Figure 5B/sh-UBC+AR/DAPI-2.tiff]

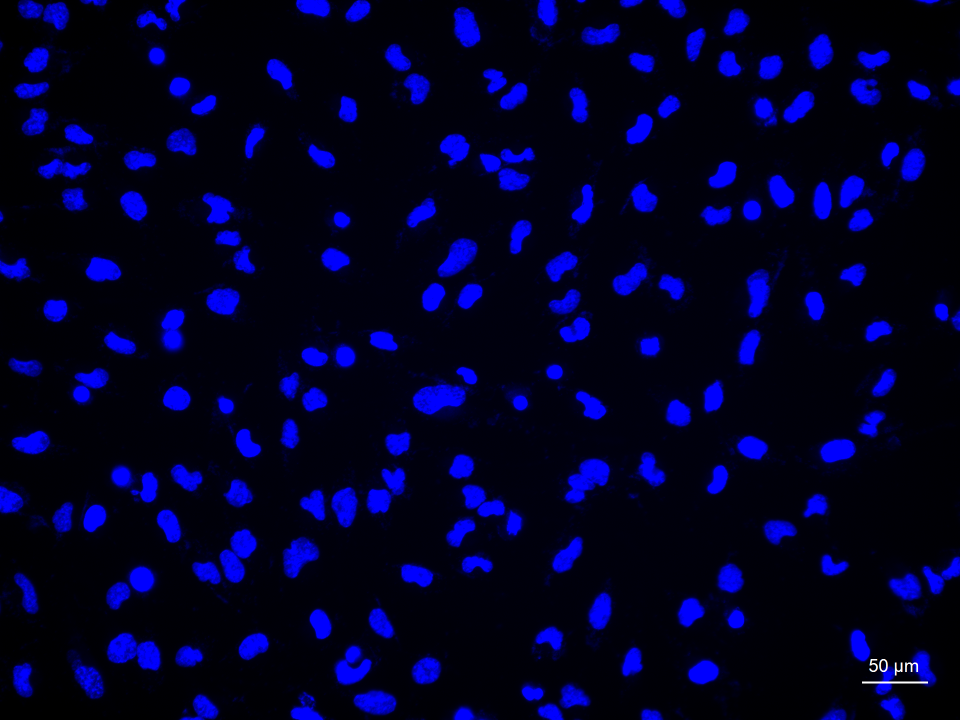

Supplement: S1 File — (ZIP) [file pone.0350815.s001.zip › Figure 5B/Control/DAPI-3.tiff]

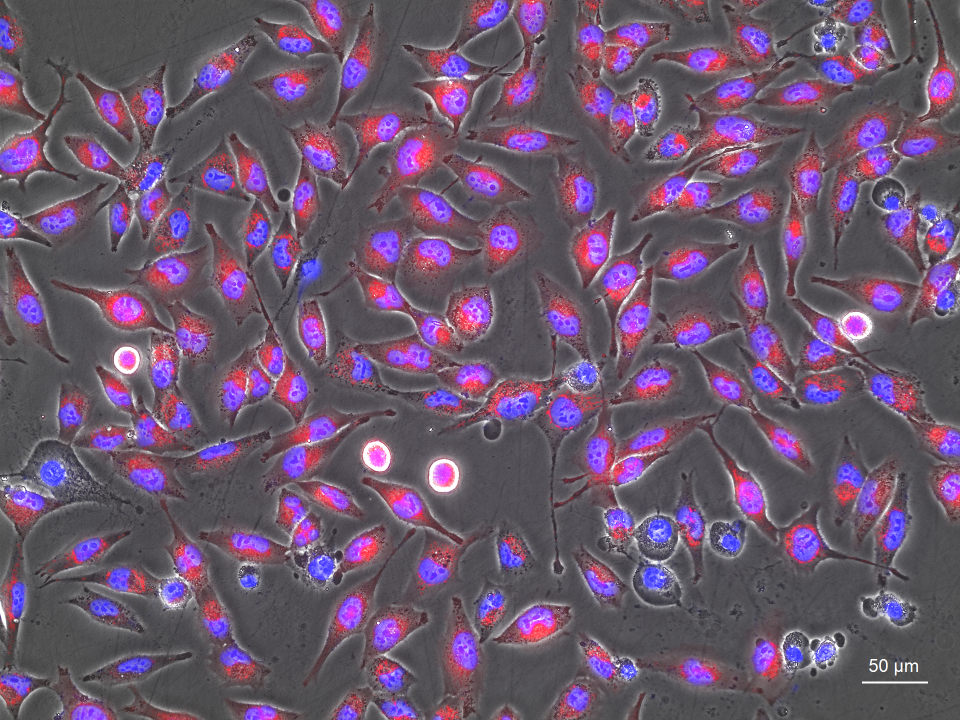

Supplement: S1 File — (ZIP) [file pone.0350815.s001.zip › Figure 5B/Control/MERGE-1.tiff]

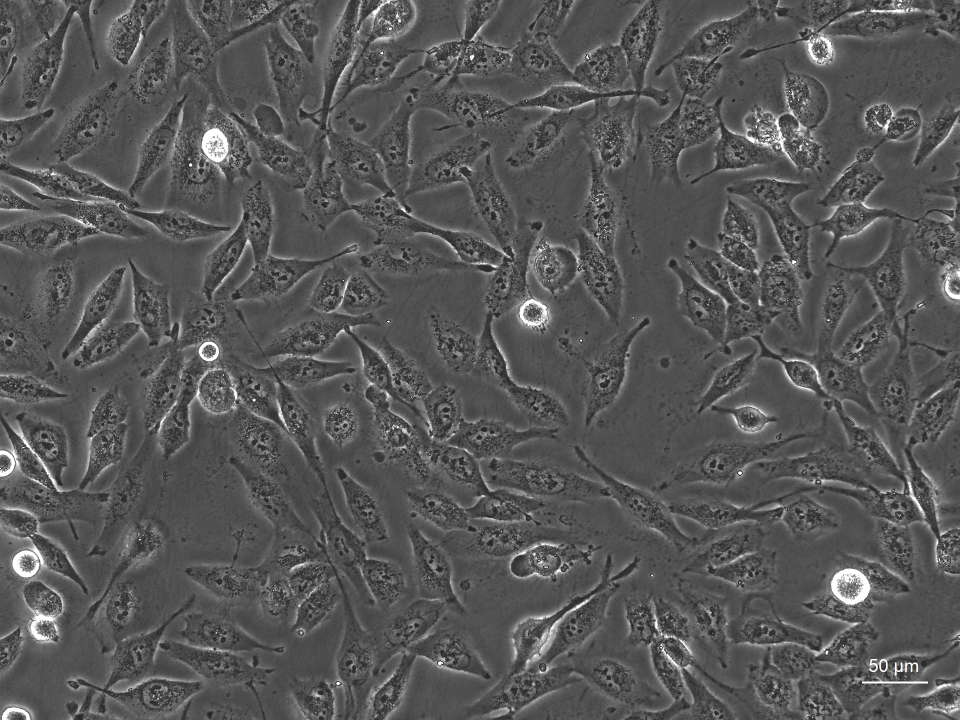

Supplement: S1 File — (ZIP) [file pone.0350815.s001.zip › Figure 5B/Control/Brightfield-2.tiff]

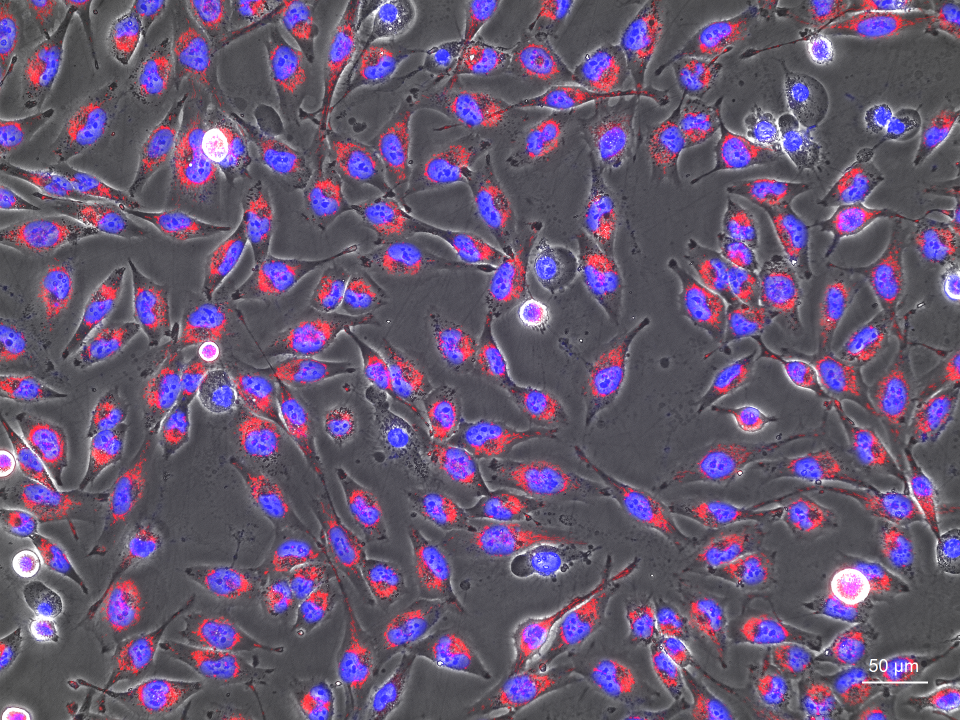

Supplement: S1 File — (ZIP) [file pone.0350815.s001.zip › Figure 5B/Control/MERGE-2.tiff]

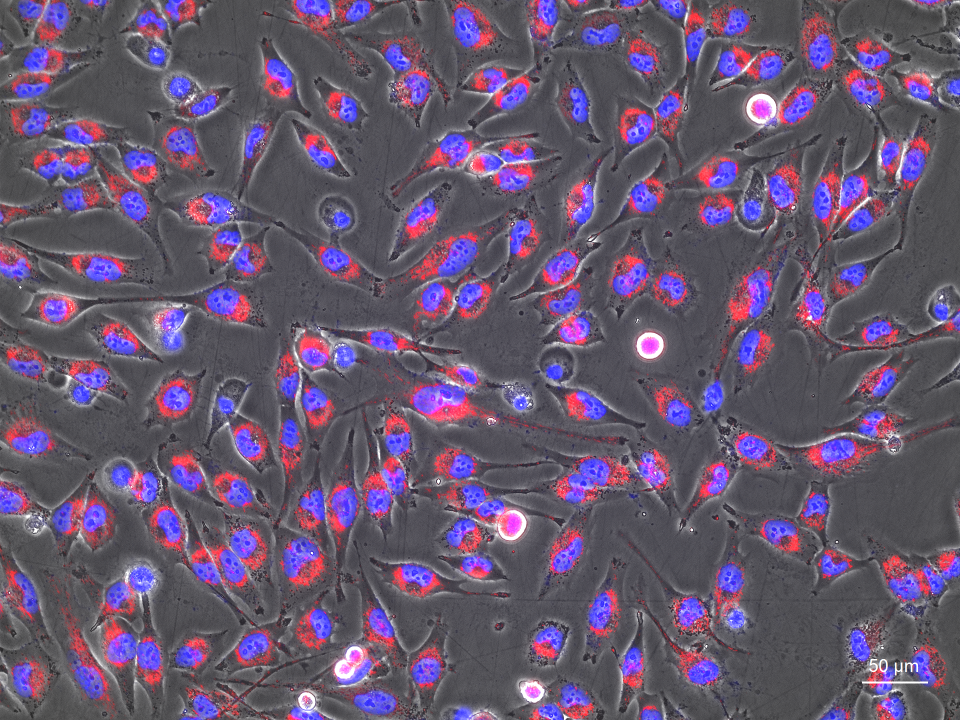

Supplement: S1 File — (ZIP) [file pone.0350815.s001.zip › Figure 5B/Control/MERGE-3.tiff]

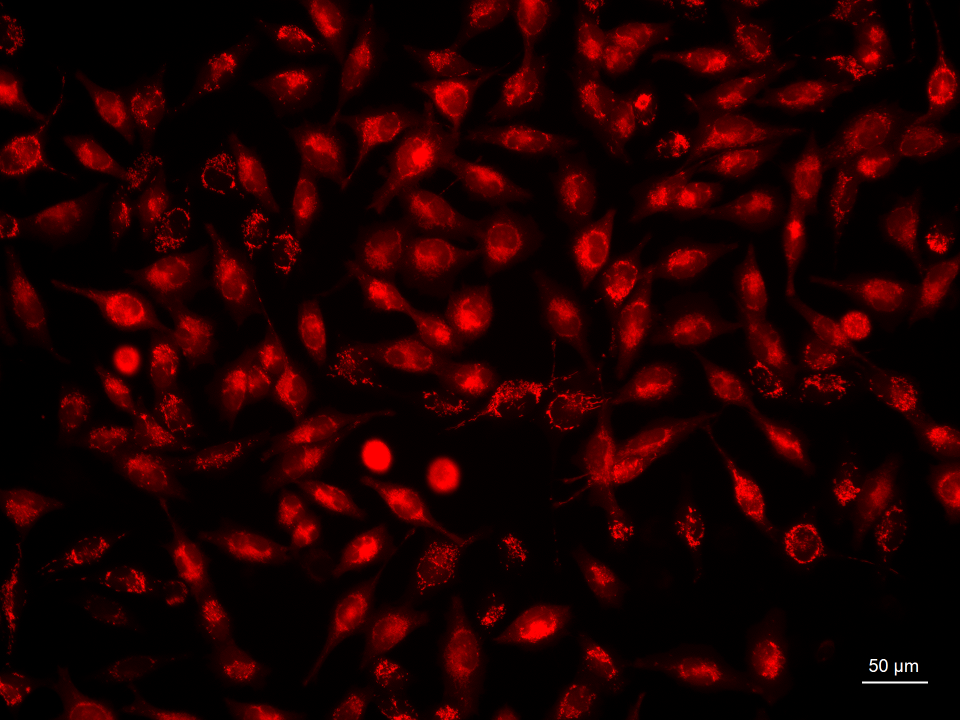

Supplement: S1 File — (ZIP) [file pone.0350815.s001.zip › Figure 5B/Control/TMRE-1.tiff]

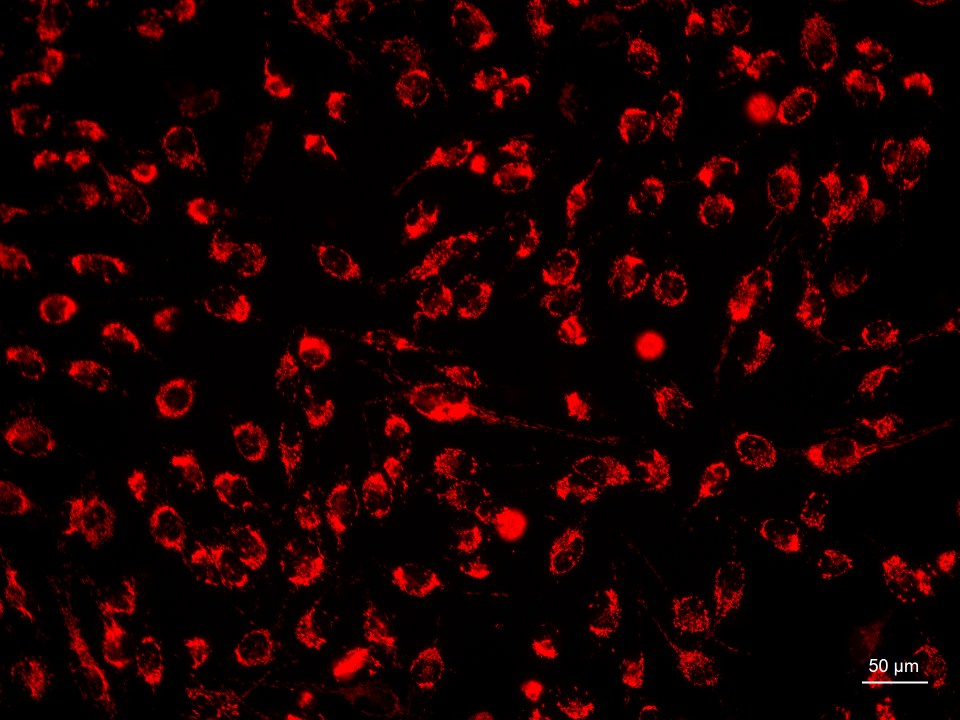

Supplement: S1 File — (ZIP) [file pone.0350815.s001.zip › Figure 5B/Control/TMRE-3.tiff]

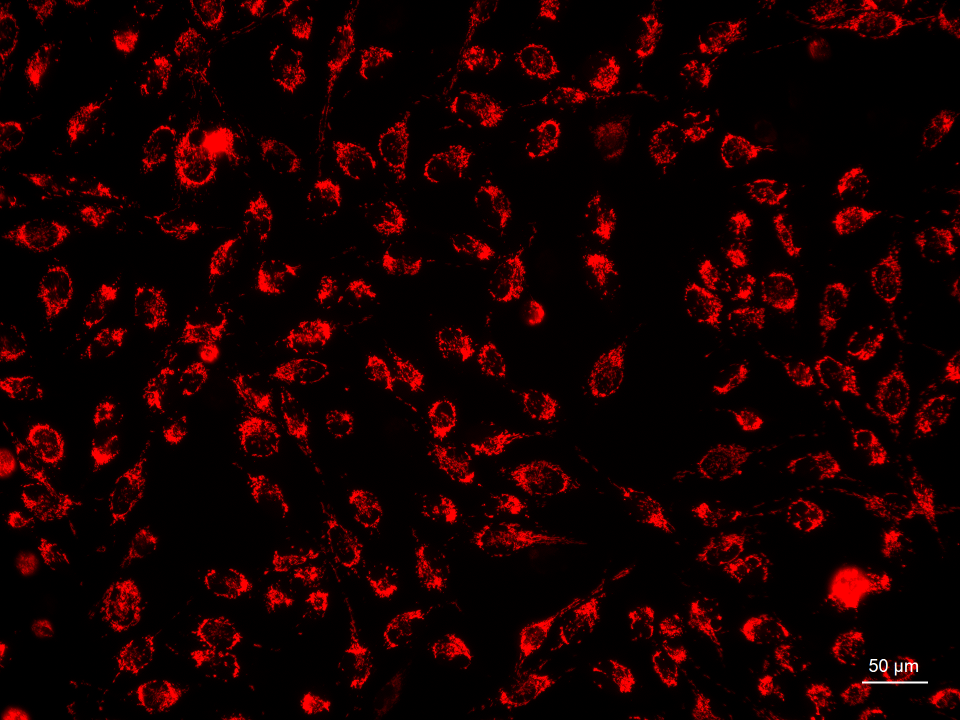

Supplement: S1 File — (ZIP) [file pone.0350815.s001.zip › Figure 5B/Control/TMRE-2.tiff]

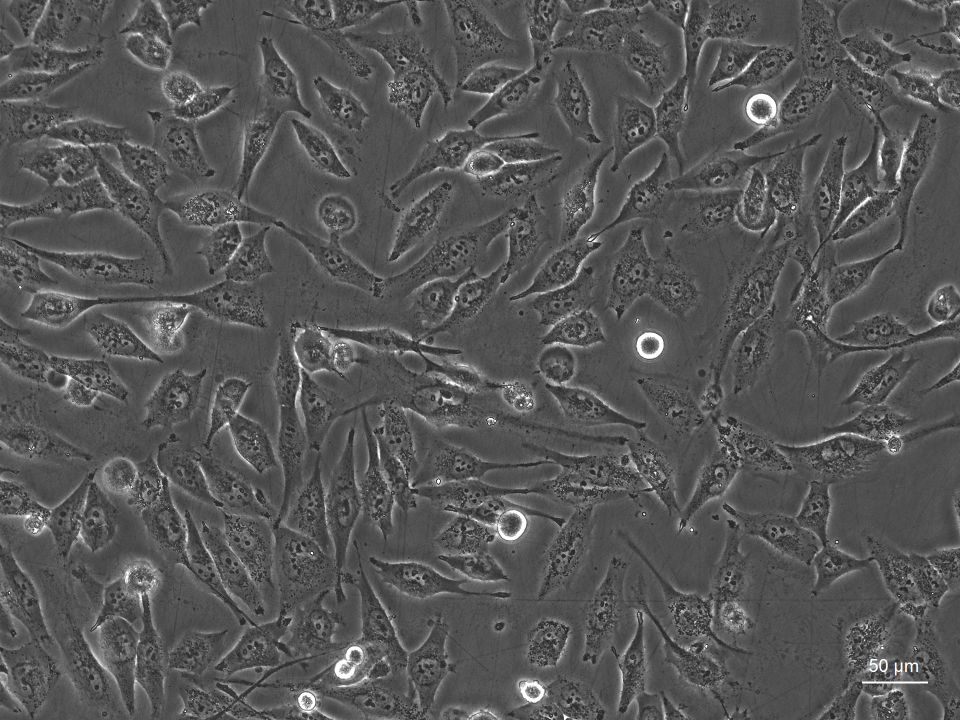

Supplement: S1 File — (ZIP) [file pone.0350815.s001.zip › Figure 5B/Control/Brightfield-3.tiff]

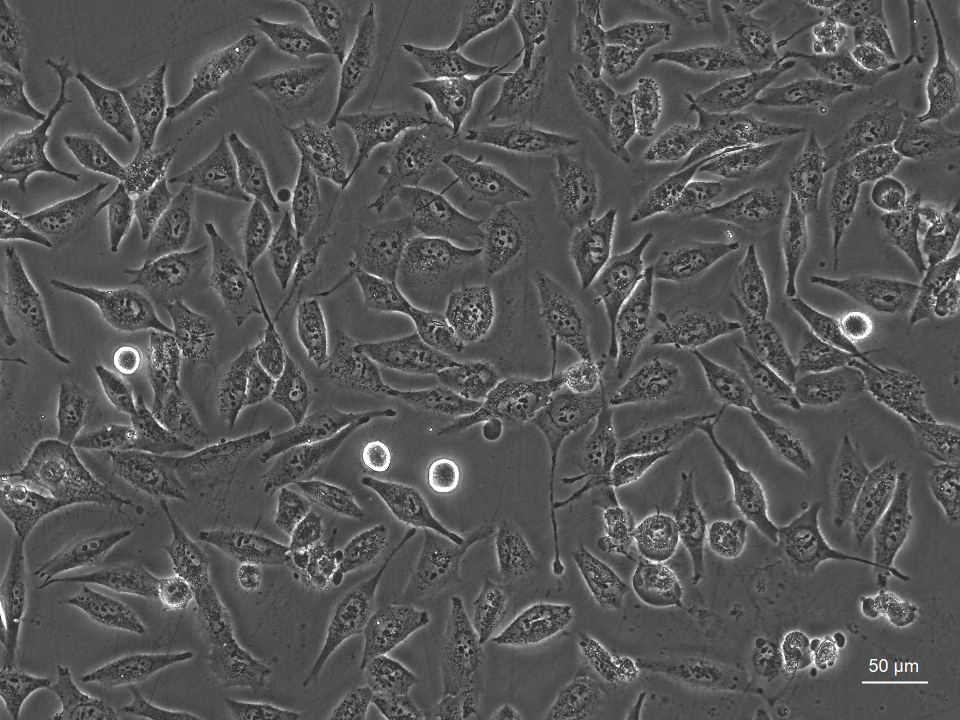

Supplement: S1 File — (ZIP) [file pone.0350815.s001.zip › Figure 5B/Control/Brightfield-1.tiff]

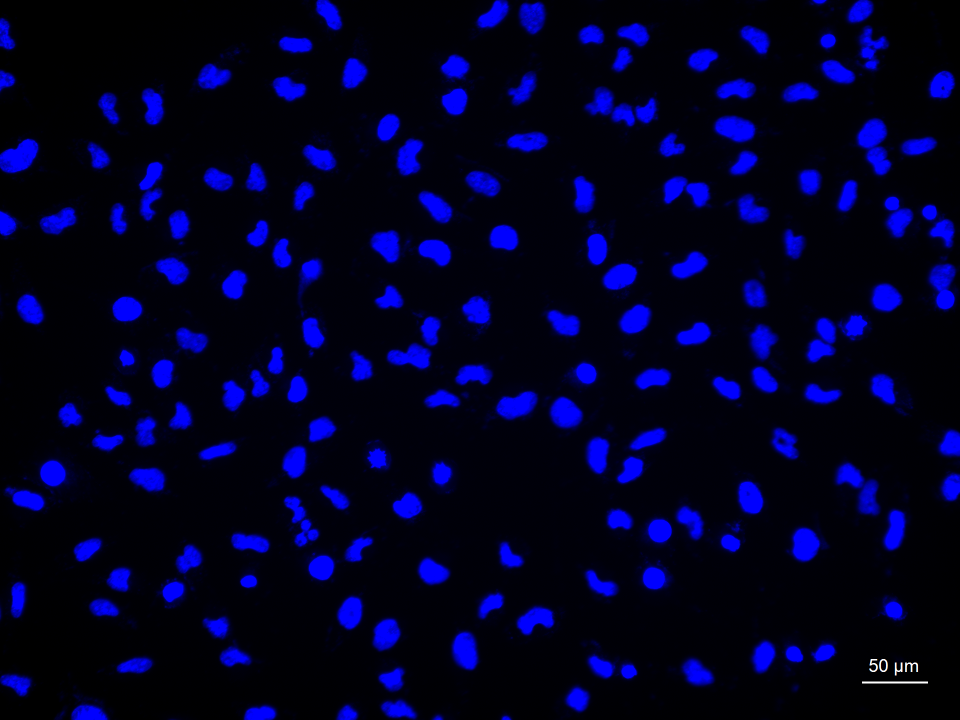

Supplement: S1 File — (ZIP) [file pone.0350815.s001.zip › Figure 5B/Control/DAPI-1.tiff]

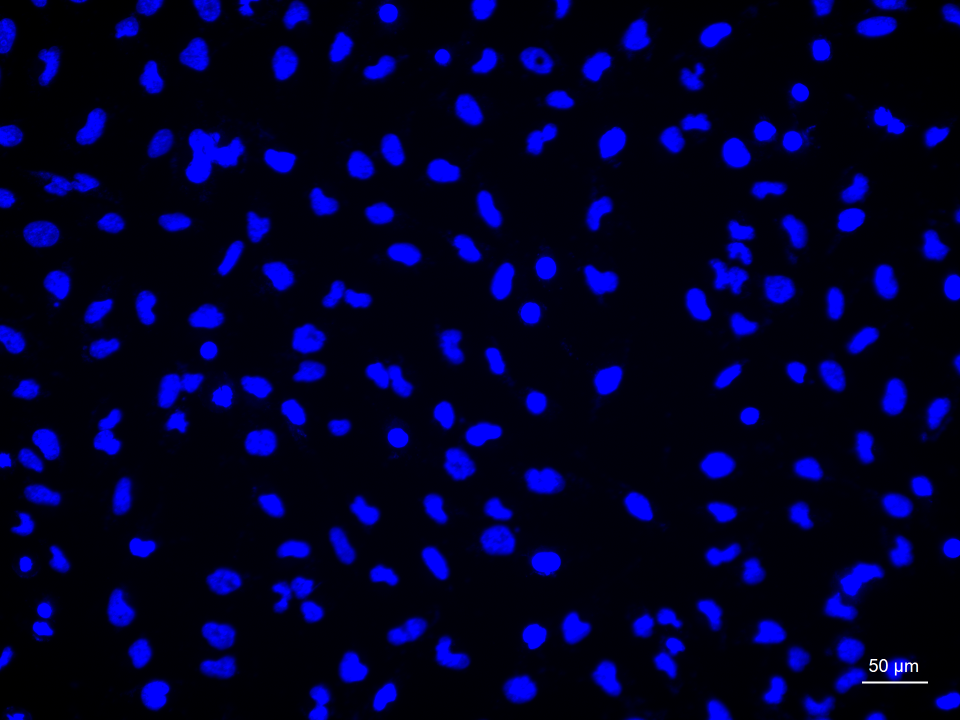

Supplement: S1 File — (ZIP) [file pone.0350815.s001.zip › Figure 5B/Control/DAPI-2.tiff]

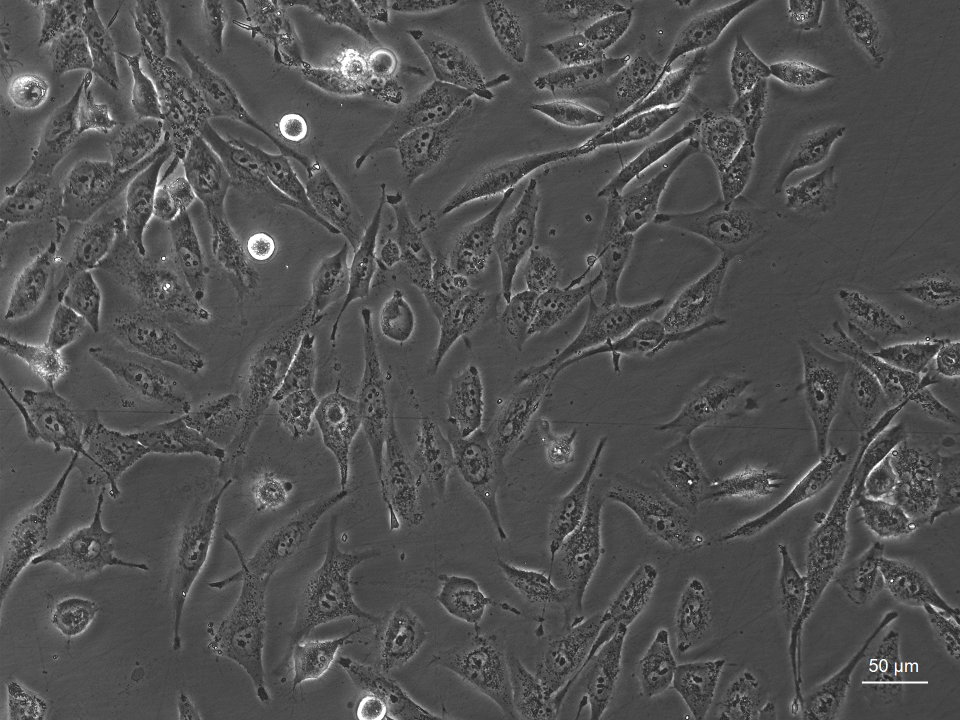

Supplement: S1 File — (ZIP) [file pone.0350815.s001.zip › Figure 5B/AR/Brightfield-3.tiff]

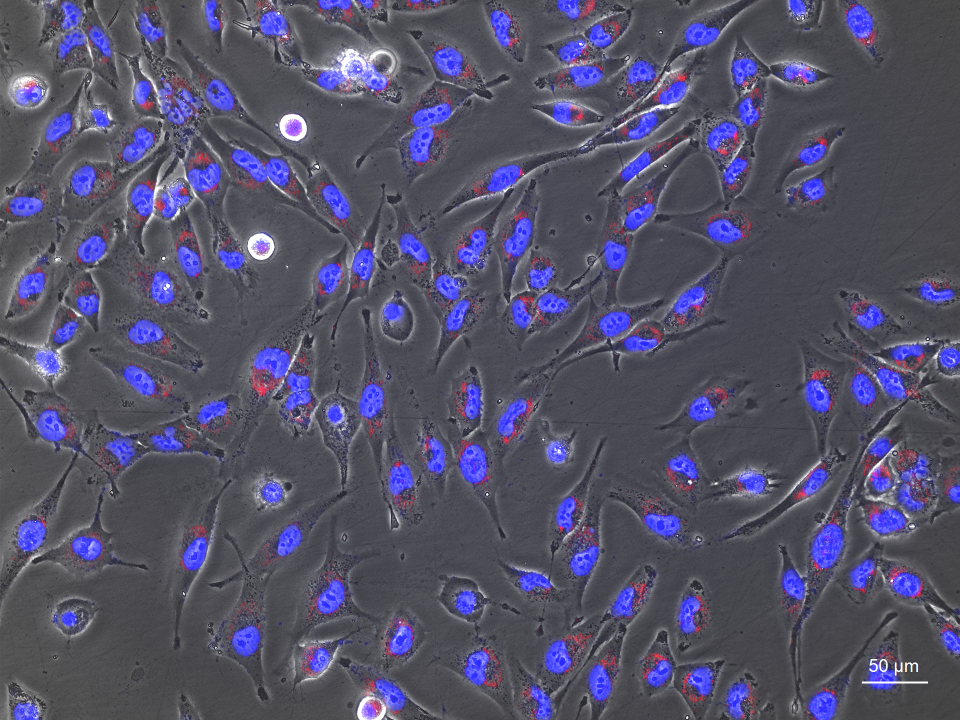

Supplement: S1 File — (ZIP) [file pone.0350815.s001.zip › Figure 5B/AR/MERGE-3.tiff]

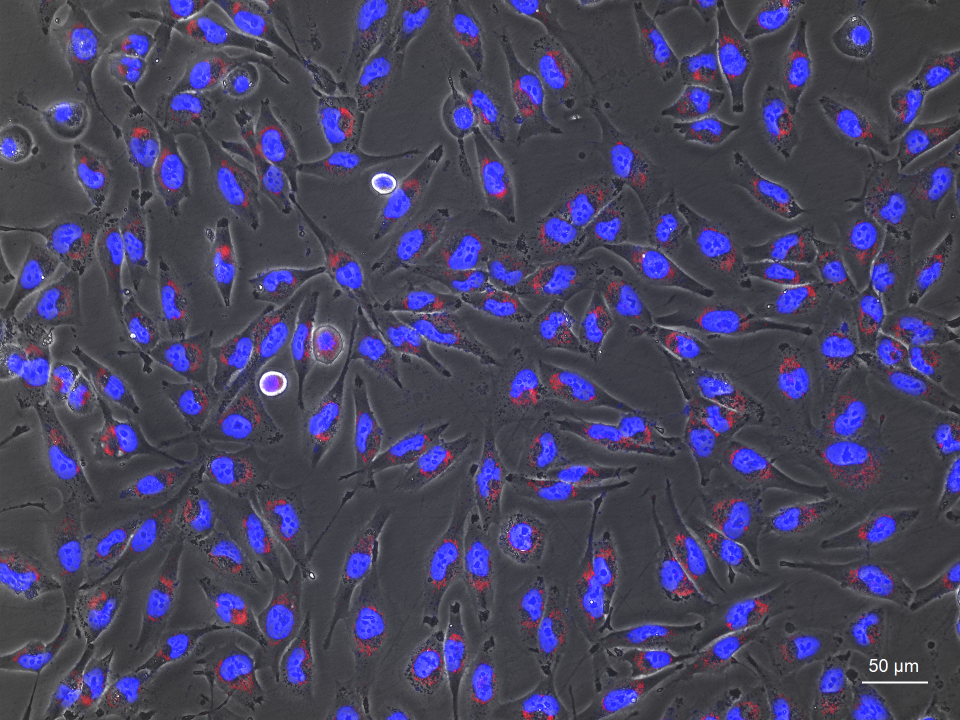

Supplement: S1 File — (ZIP) [file pone.0350815.s001.zip › Figure 5B/AR/MERGE-2.tiff]

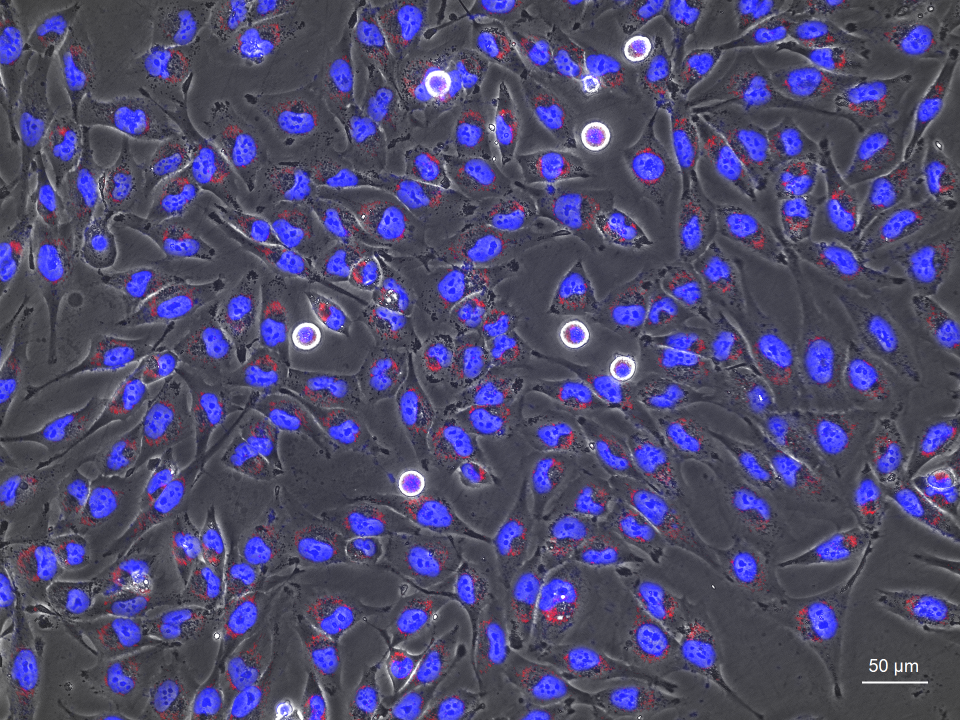

Supplement: S1 File — (ZIP) [file pone.0350815.s001.zip › Figure 5B/AR/MERGE-1.tiff]

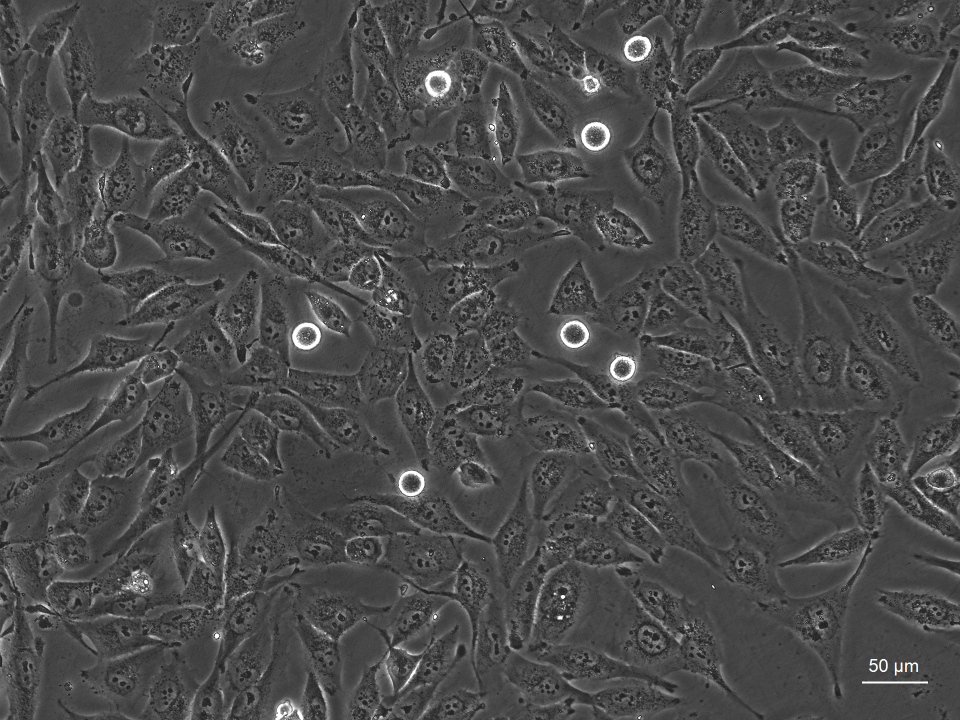

Supplement: S1 File — (ZIP) [file pone.0350815.s001.zip › Figure 5B/AR/Brightfield-1.tiff]

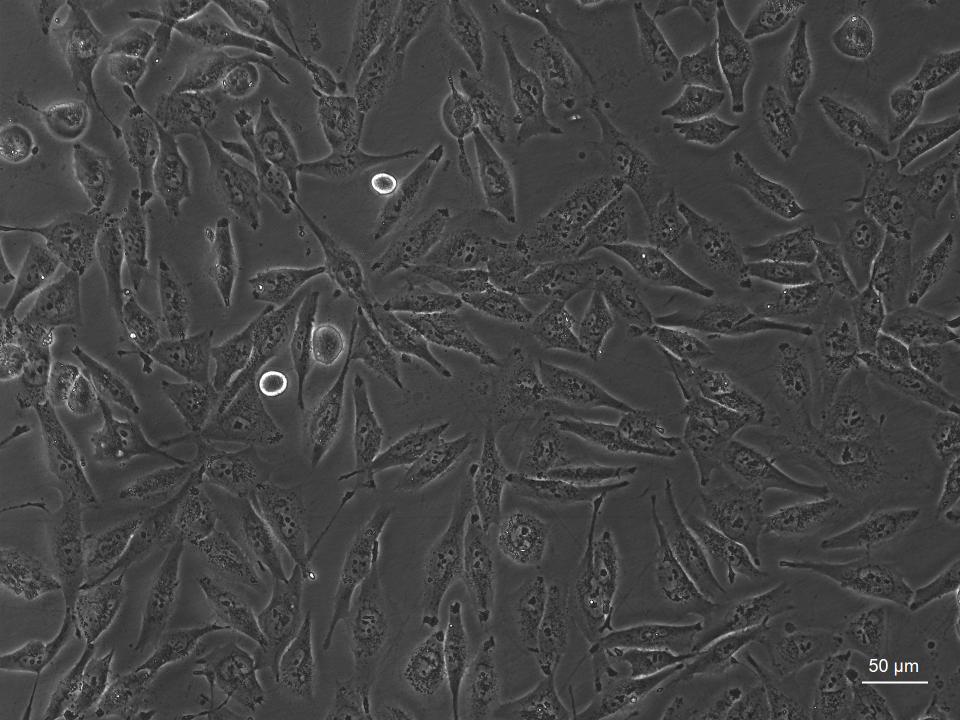

Supplement: S1 File — (ZIP) [file pone.0350815.s001.zip › Figure 5B/AR/Brightfield-2.tiff]

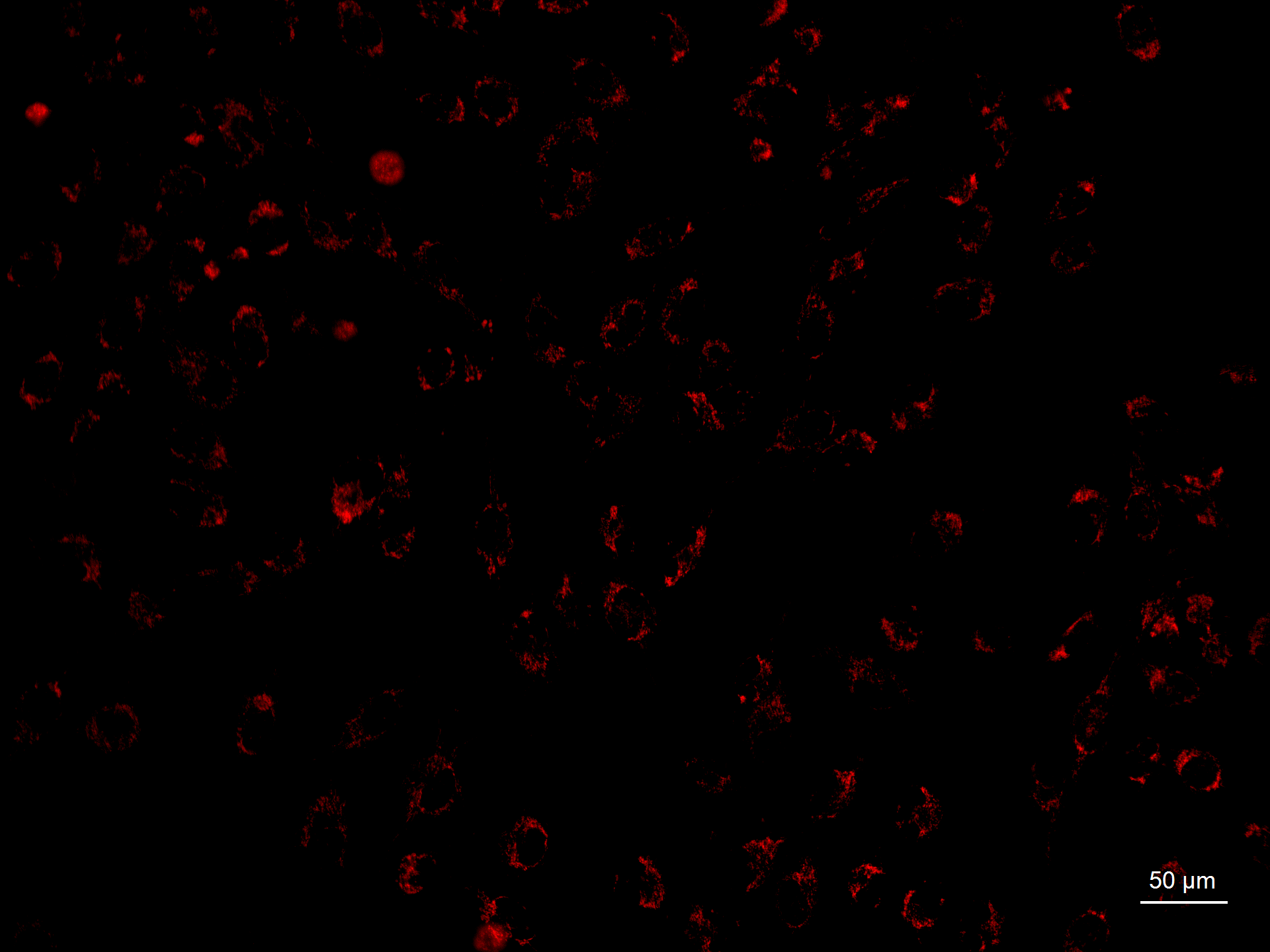

Supplement: S1 File — (ZIP) [file pone.0350815.s001.zip › Figure 5B/AR/TMRE-3.tif]

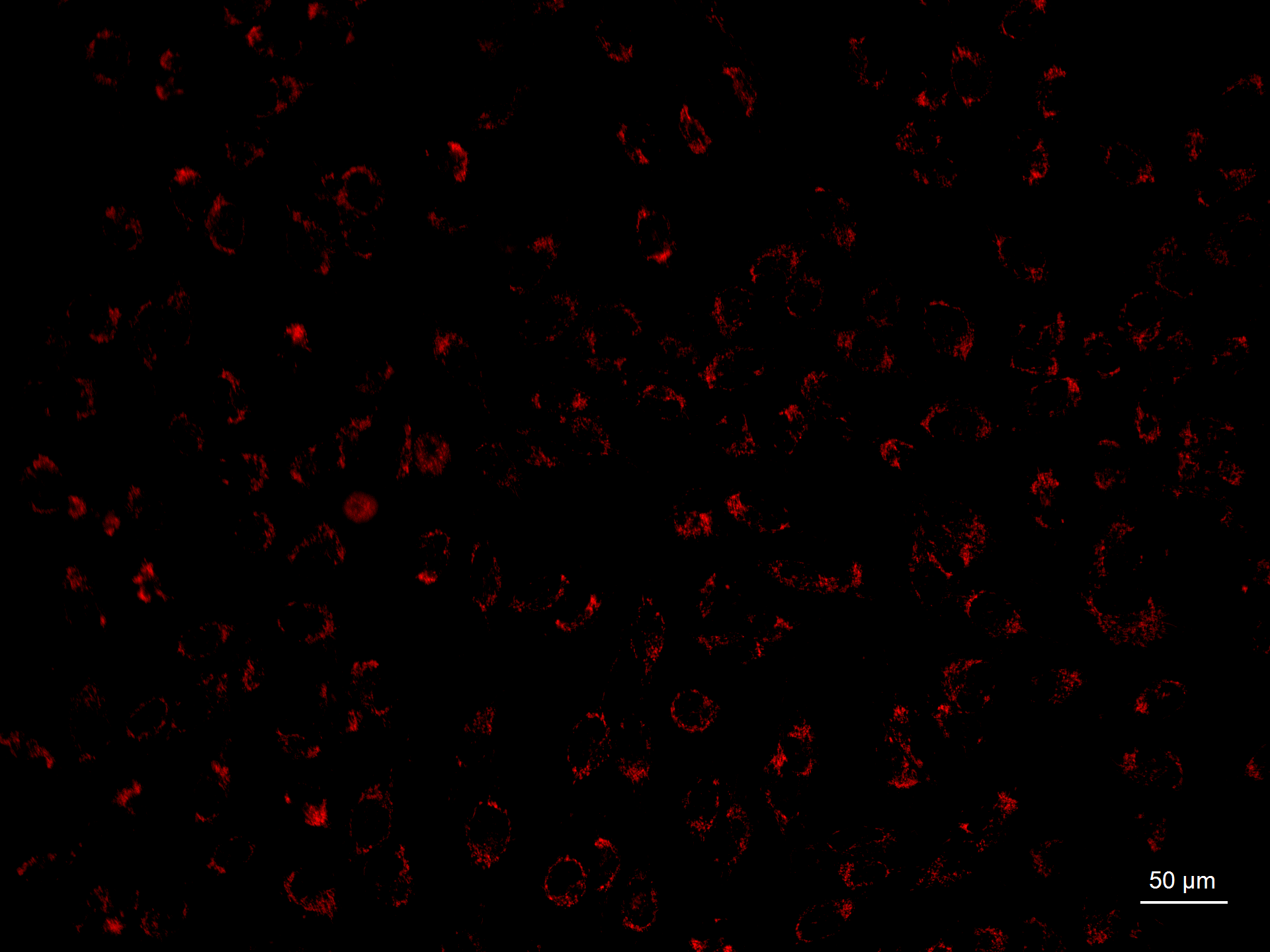

Supplement: S1 File — (ZIP) [file pone.0350815.s001.zip › Figure 5B/AR/TMRE-2.tif]

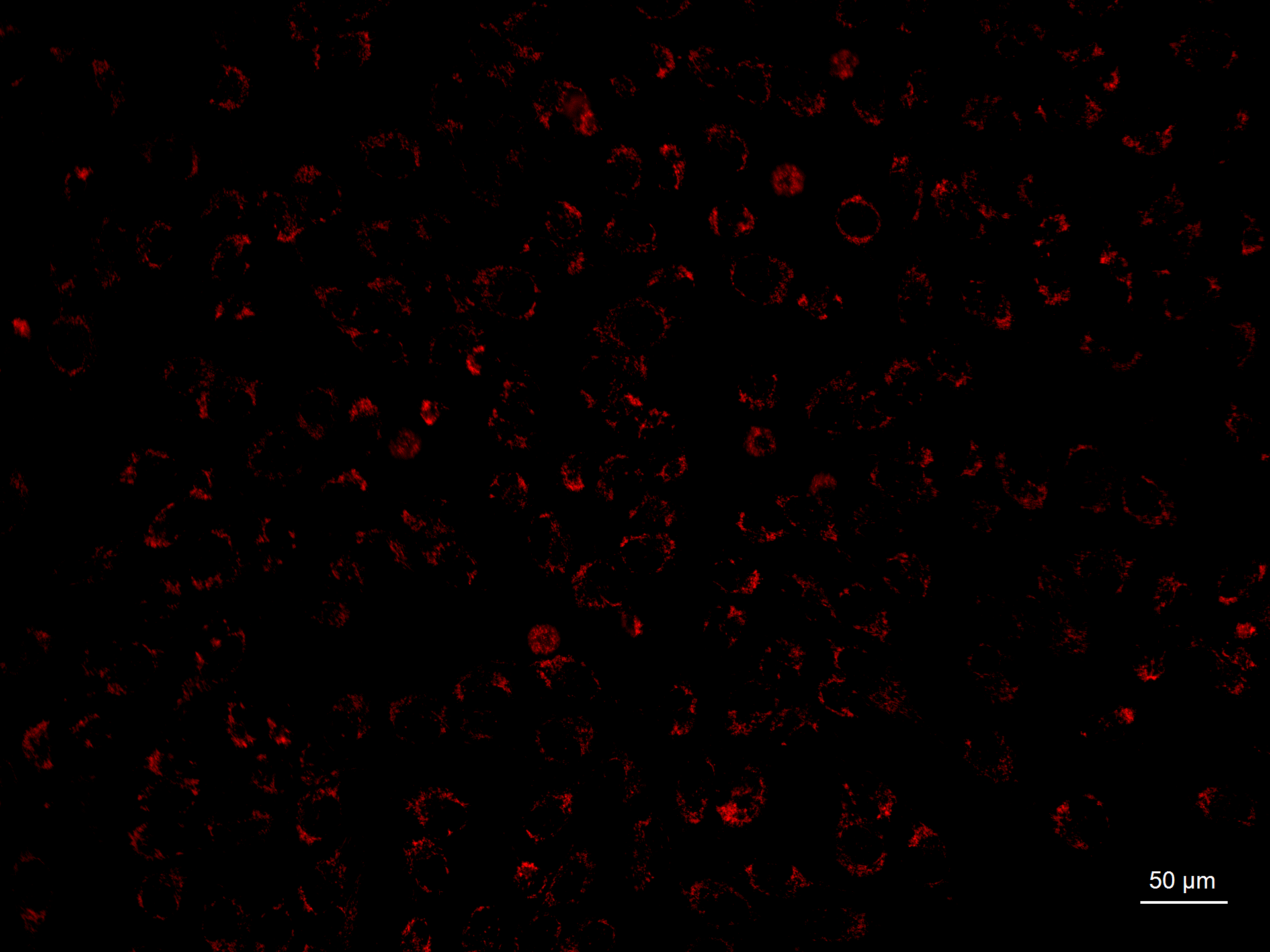

Supplement: S1 File — (ZIP) [file pone.0350815.s001.zip › Figure 5B/AR/TMRE-1.tif]

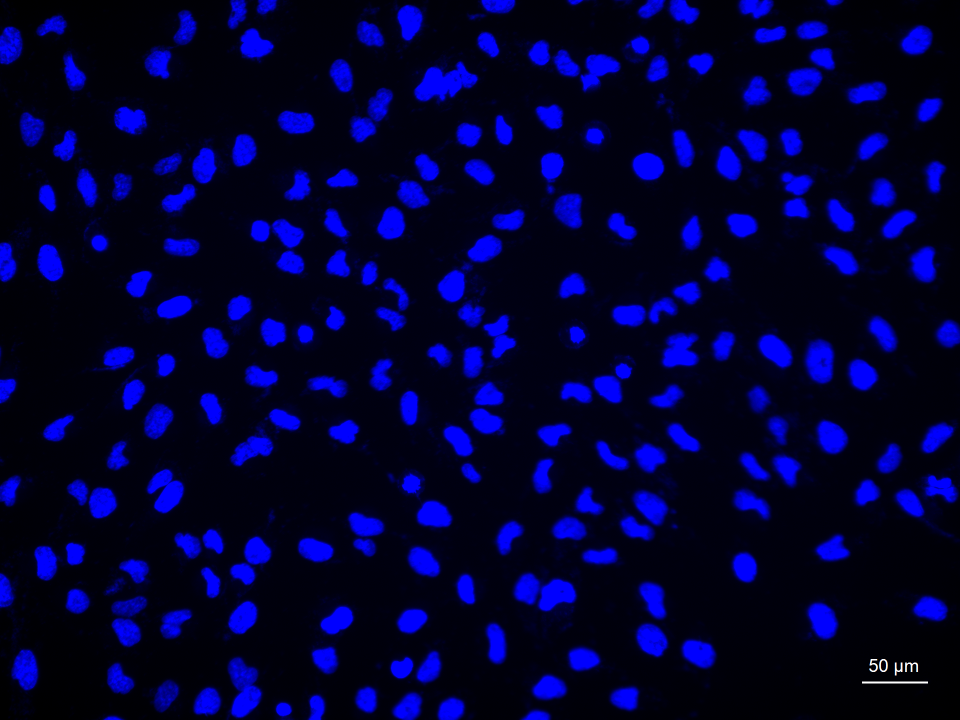

Supplement: S1 File — (ZIP) [file pone.0350815.s001.zip › Figure 5B/AR/DAPI-1.tiff]

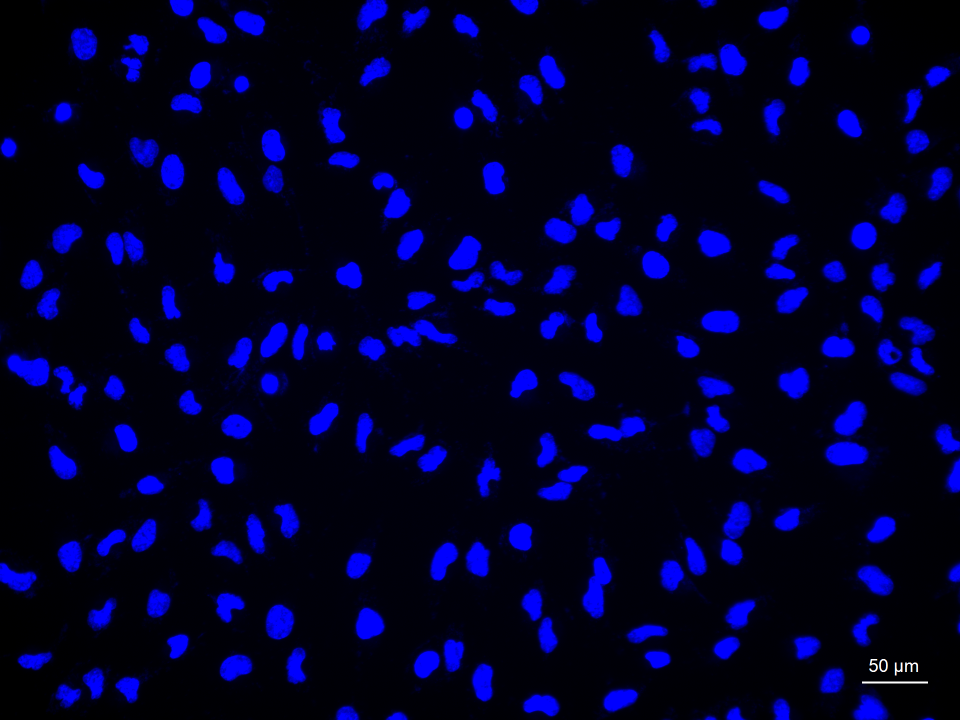

Supplement: S1 File — (ZIP) [file pone.0350815.s001.zip › Figure 5B/AR/DAPI-2.tiff]

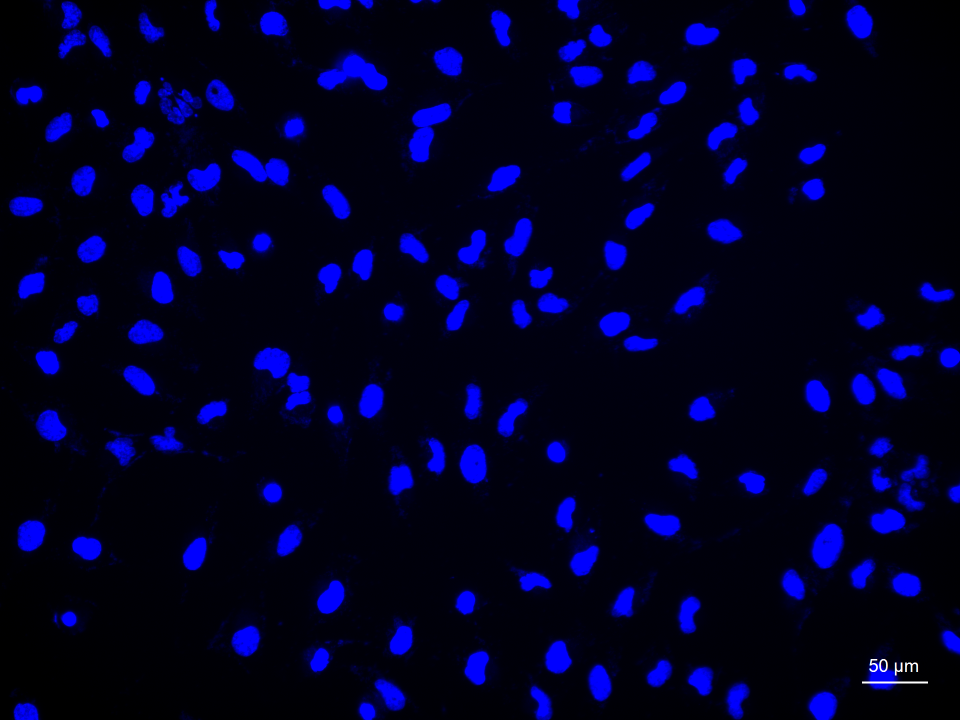

Supplement: S1 File — (ZIP) [file pone.0350815.s001.zip › Figure 5B/AR/DAPI-3.tiff]

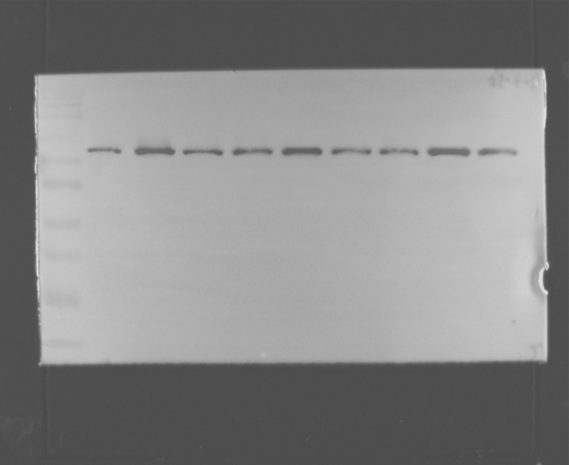

Supplement: S1 File — (ZIP) [file pone.0350815.s001.zip › Figure 4F/UBC 60KDa 01-03.tiff]

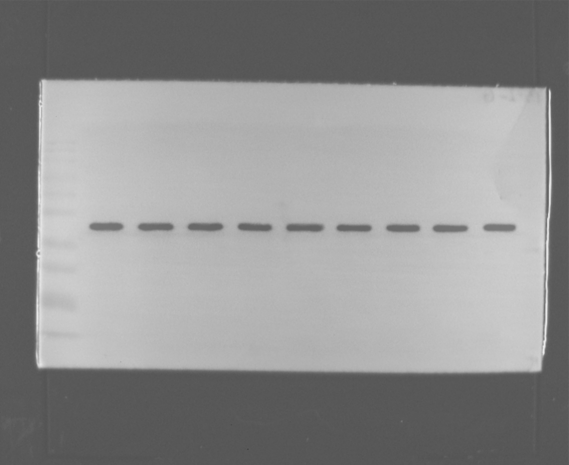

Supplement: S1 File — (ZIP) [file pone.0350815.s001.zip › Figure 4F/GAPDH 37KDa 01-03.tiff]

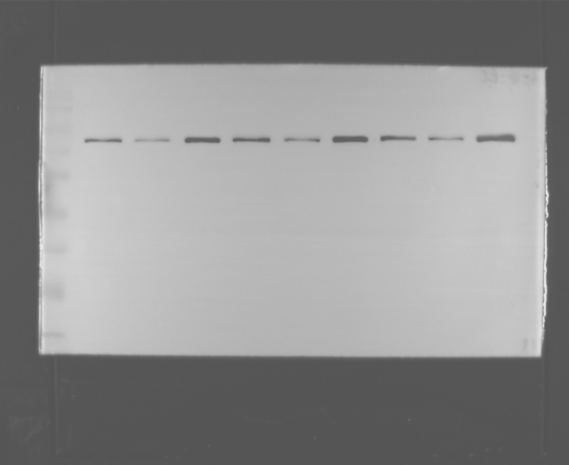

Supplement: S1 File — (ZIP) [file pone.0350815.s001.zip › Figure 4F/PINK 63KDa 01-03.tiff]

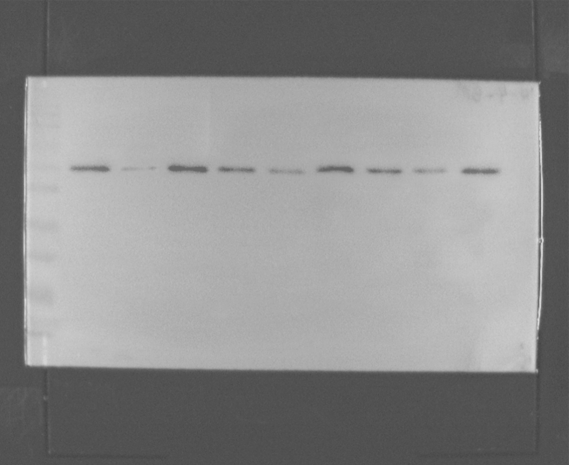

Supplement: S1 File — (ZIP) [file pone.0350815.s001.zip › Figure 4F/Parkin 55KDa 01-03.tiff]

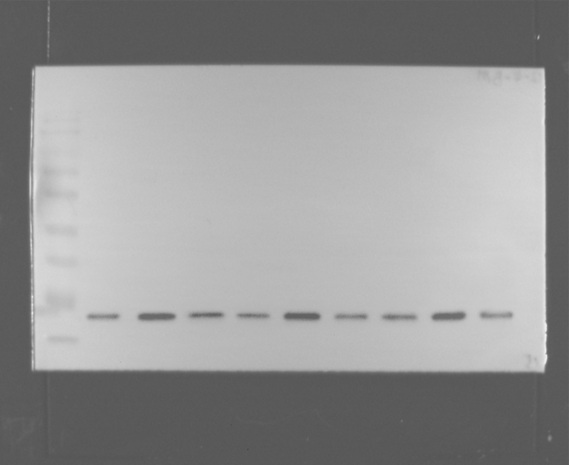

Supplement: S1 File — (ZIP) [file pone.0350815.s001.zip › Figure 4F/UBA52 15KDa 01-03.tiff]

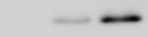

Supplement: S1 File — (ZIP) [file pone.0350815.s001.zip › Figure 4A/UBC IP 01.tiff]

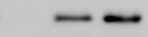

Supplement: S1 File — (ZIP) [file pone.0350815.s001.zip › Figure 4A/UBA52 IP 03.tiff]

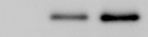

Supplement: S1 File — (ZIP) [file pone.0350815.s001.zip › Figure 4A/UBA52 IP 01.tiff]

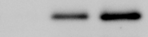

Supplement: S1 File — (ZIP) [file pone.0350815.s001.zip › Figure 4A/UBA52 IP 02.tiff]

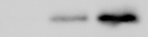

Supplement: S1 File — (ZIP) [file pone.0350815.s001.zip › Figure 4A/UBC IP 03.tiff]

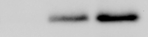

Supplement: S1 File — (ZIP) [file pone.0350815.s001.zip › Figure 4A/UBC IP 02.tiff]

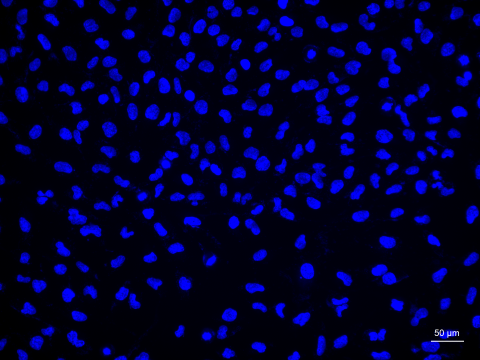

Supplement: S1 File — (ZIP) [file pone.0350815.s001.zip › Figure 3A/Control/DAPI-3.tiff]

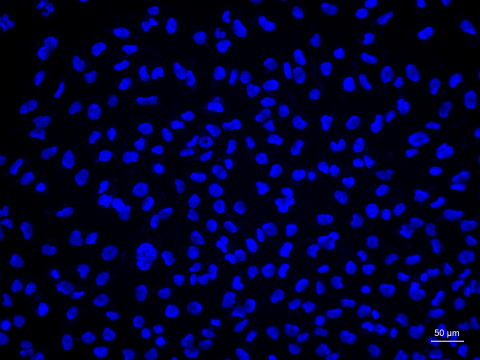

Supplement: S1 File — (ZIP) [file pone.0350815.s001.zip › Figure 3A/Control/DAPI-2.tiff]

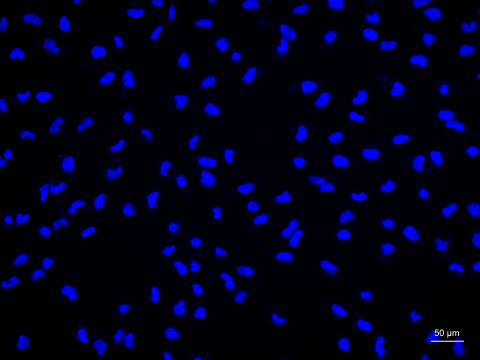

Supplement: S1 File — (ZIP) [file pone.0350815.s001.zip › Figure 3A/Control/DAPI-1.tiff]

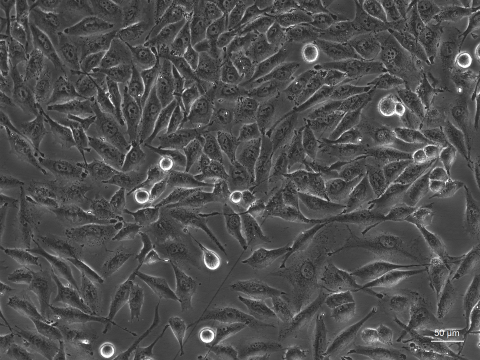

Supplement: S1 File — (ZIP) [file pone.0350815.s001.zip › Figure 3A/Control/Brightfield-3.tiff]

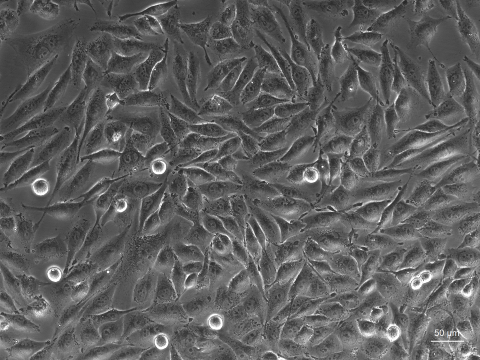

Supplement: S1 File — (ZIP) [file pone.0350815.s001.zip › Figure 3A/Control/Brightfield-2.tiff]

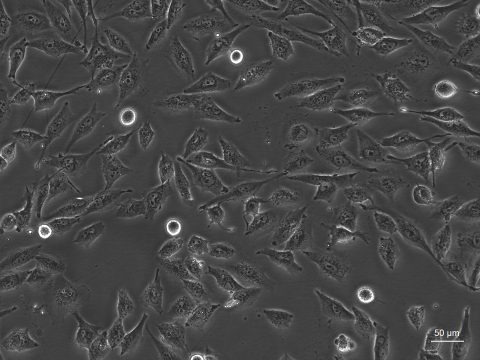

Supplement: S1 File — (ZIP) [file pone.0350815.s001.zip › Figure 3A/Control/Brightfield-1.tiff]

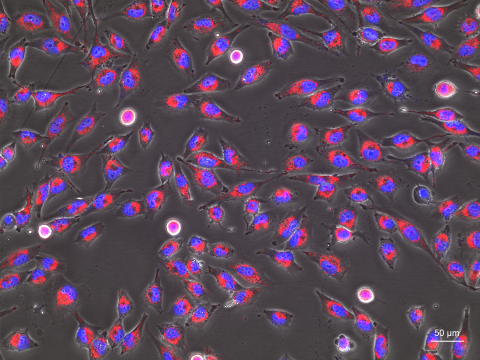

Supplement: S1 File — (ZIP) [file pone.0350815.s001.zip › Figure 3A/Control/MERGE-1.tiff]

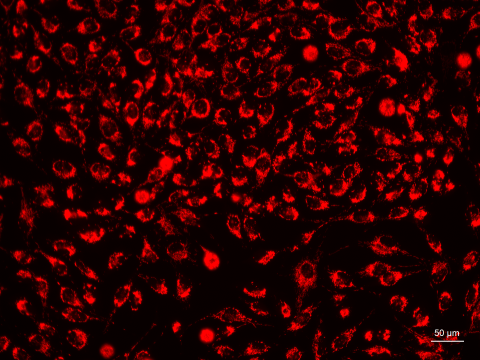

Supplement: S1 File — (ZIP) [file pone.0350815.s001.zip › Figure 3A/Control/TMRE-3.tiff]

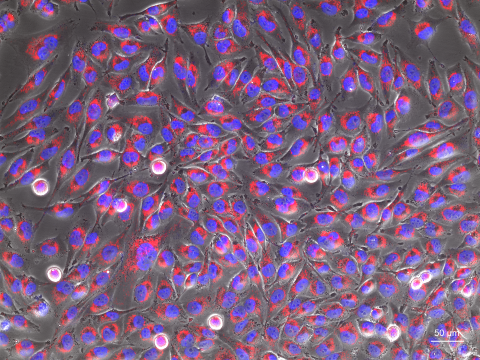

Supplement: S1 File — (ZIP) [file pone.0350815.s001.zip › Figure 3A/Control/MERGE-2.tiff]

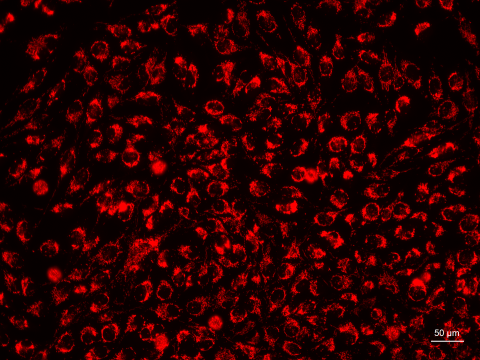

Supplement: S1 File — (ZIP) [file pone.0350815.s001.zip › Figure 3A/Control/TMRE-2.tiff]

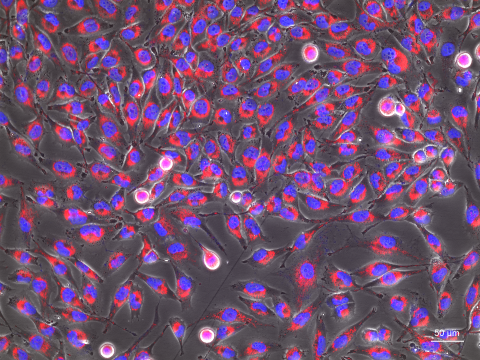

Supplement: S1 File — (ZIP) [file pone.0350815.s001.zip › Figure 3A/Control/MERGE-3.tiff]

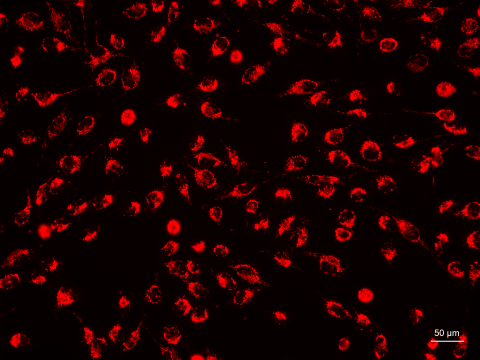

Supplement: S1 File — (ZIP) [file pone.0350815.s001.zip › Figure 3A/Control/TMRE-1.tiff]

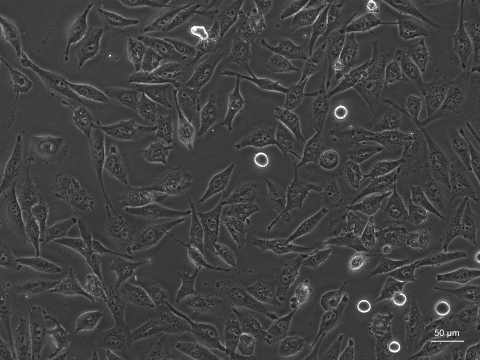

Supplement: S1 File — (ZIP) [file pone.0350815.s001.zip › Figure 3A/AR/Brightfield-2.tiff]

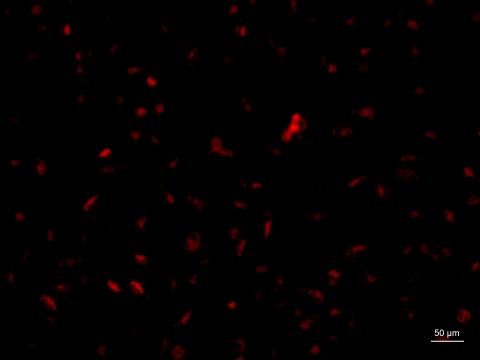

Supplement: S1 File — (ZIP) [file pone.0350815.s001.zip › Figure 3A/AR/TMRE-1.tiff]

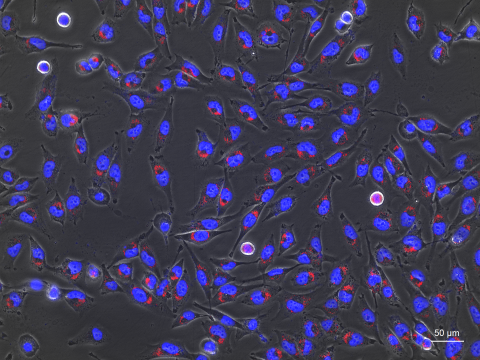

Supplement: S1 File — (ZIP) [file pone.0350815.s001.zip › Figure 3A/AR/MERGE-3.tiff]

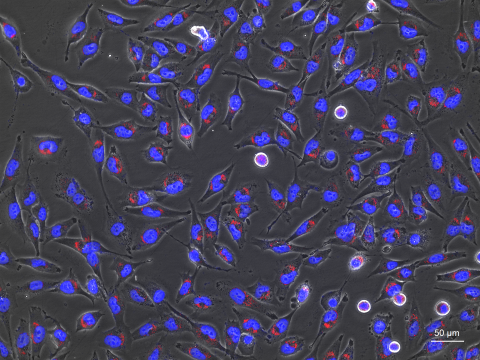

Supplement: S1 File — (ZIP) [file pone.0350815.s001.zip › Figure 3A/AR/MERGE-2.tiff]

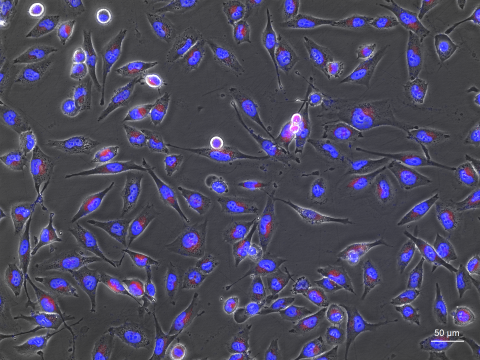

Supplement: S1 File — (ZIP) [file pone.0350815.s001.zip › Figure 3A/AR/MERGE-1.tiff]

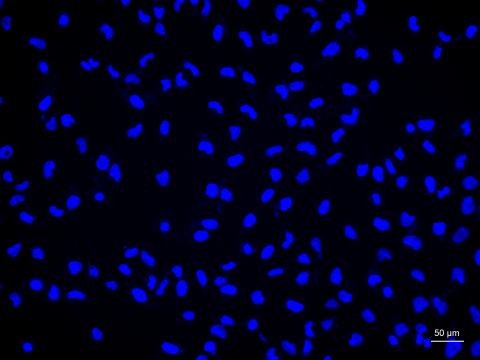

Supplement: S1 File — (ZIP) [file pone.0350815.s001.zip › Figure 3A/AR/DAPI-3.tiff]

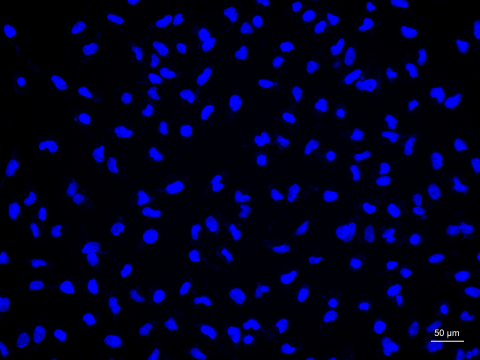

Supplement: S1 File — (ZIP) [file pone.0350815.s001.zip › Figure 3A/AR/DAPI-2.tiff]

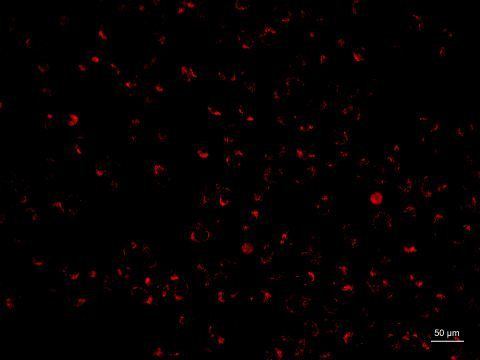

Supplement: S1 File — (ZIP) [file pone.0350815.s001.zip › Figure 3A/AR/TMRE-3.tiff]

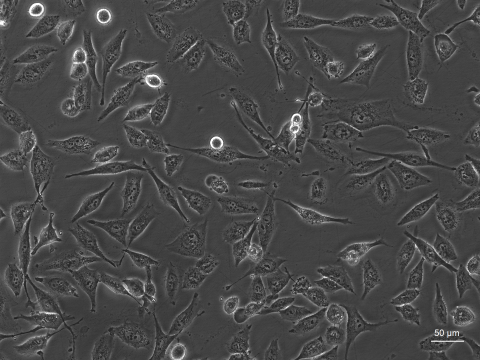

Supplement: S1 File — (ZIP) [file pone.0350815.s001.zip › Figure 3A/AR/Brightfield-1.tiff]

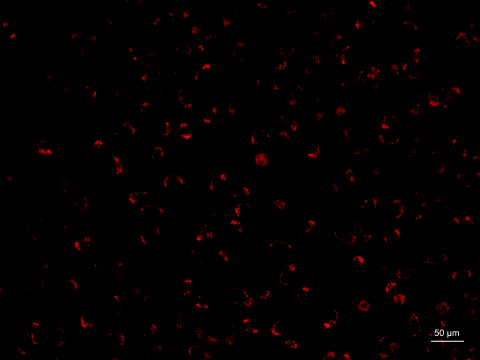

Supplement: S1 File — (ZIP) [file pone.0350815.s001.zip › Figure 3A/AR/TMRE-2.tiff]

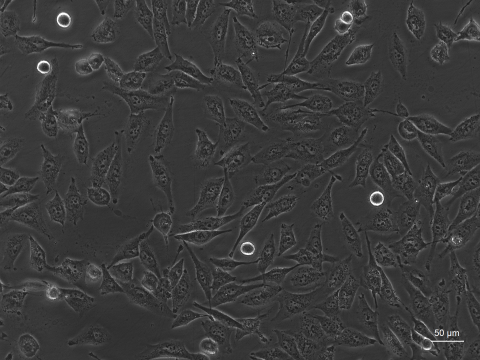

Supplement: S1 File — (ZIP) [file pone.0350815.s001.zip › Figure 3A/AR/Brightfield-3.tiff]

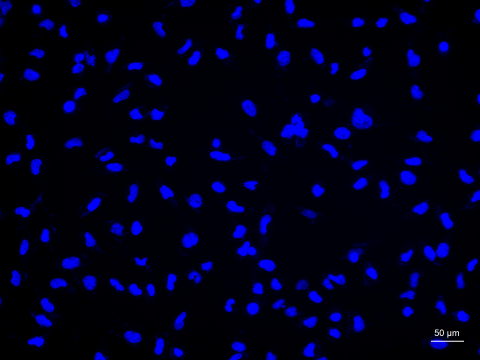

Supplement: S1 File — (ZIP) [file pone.0350815.s001.zip › Figure 3A/AR/DAPI-1.tiff]

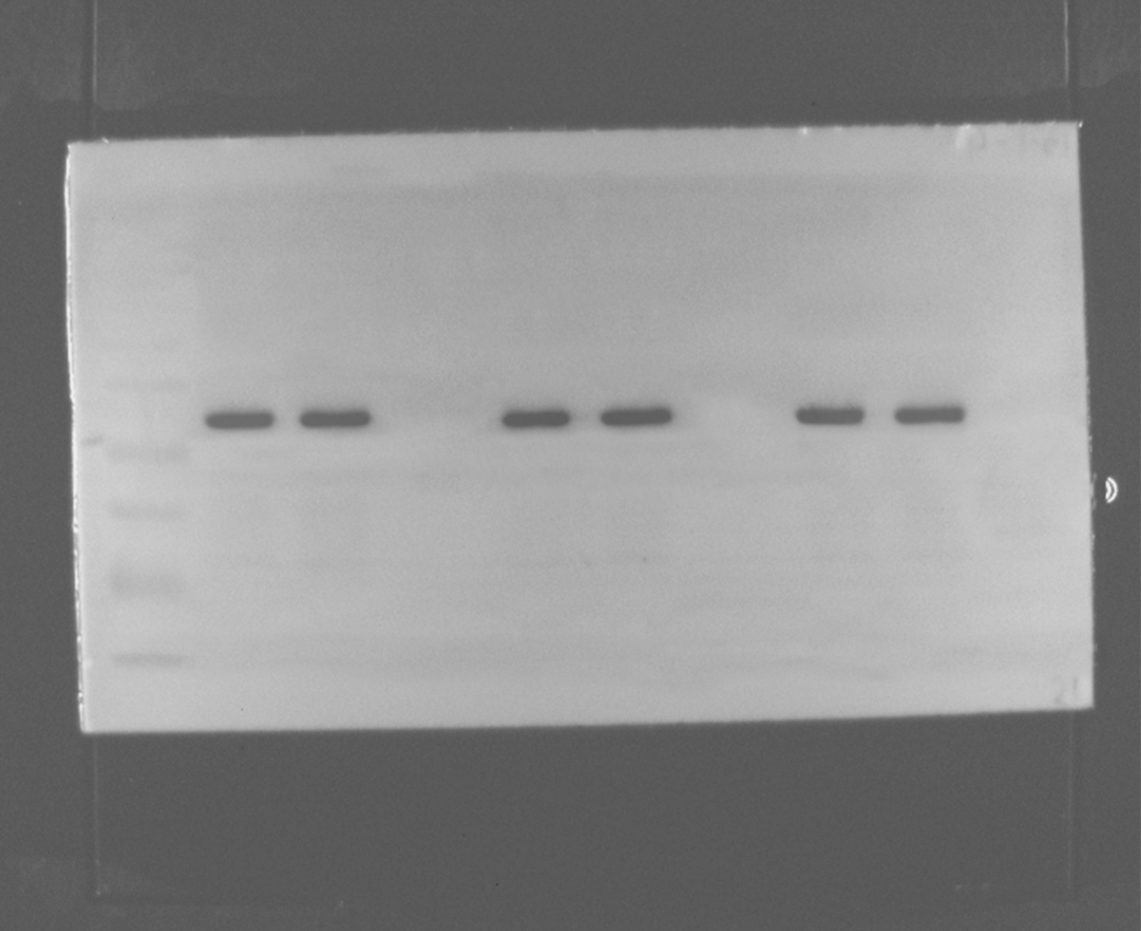

Supplement: S1 File — (ZIP) [file pone.0350815.s001.zip › Figure 2E/GAPDH 37KDa 01-03.tiff]

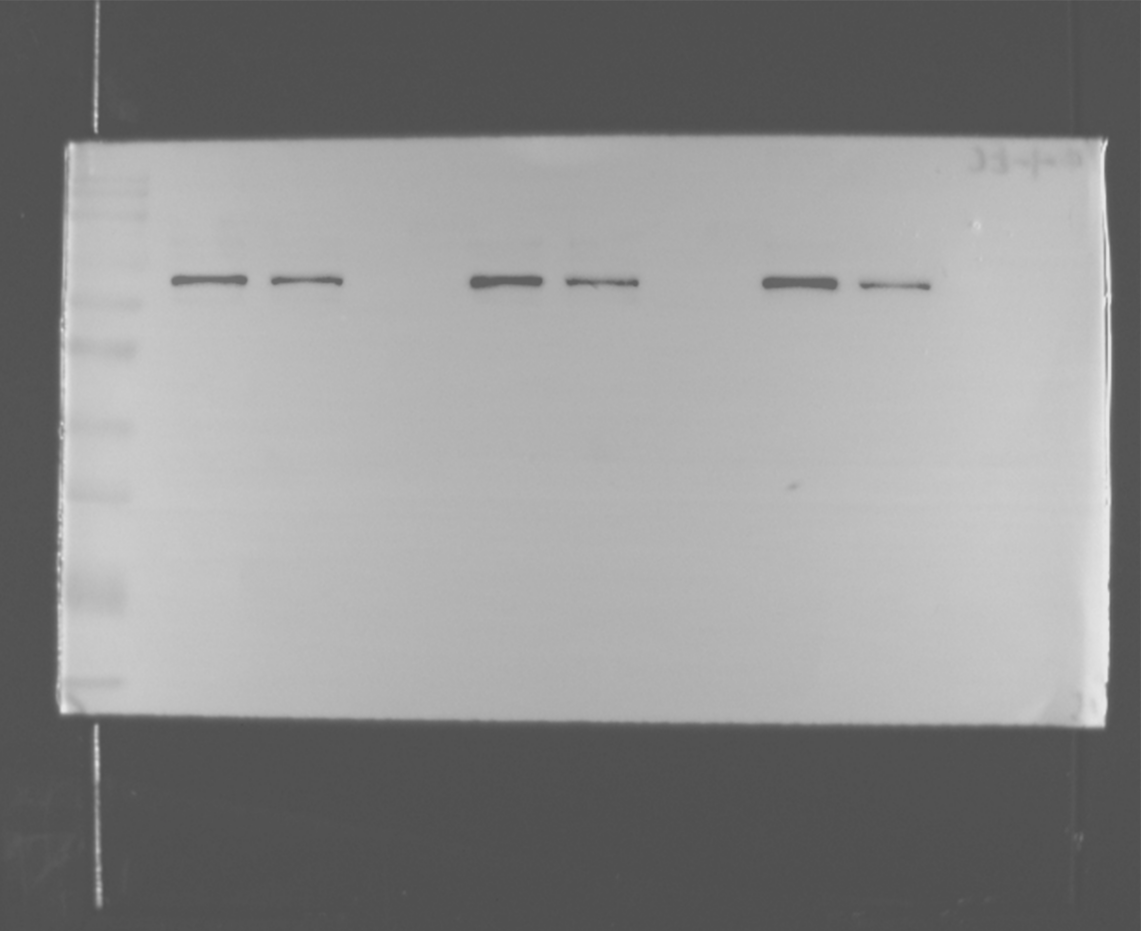

Supplement: S1 File — (ZIP) [file pone.0350815.s001.zip › Figure 2E/PINK 63KDa 01-03.tiff]

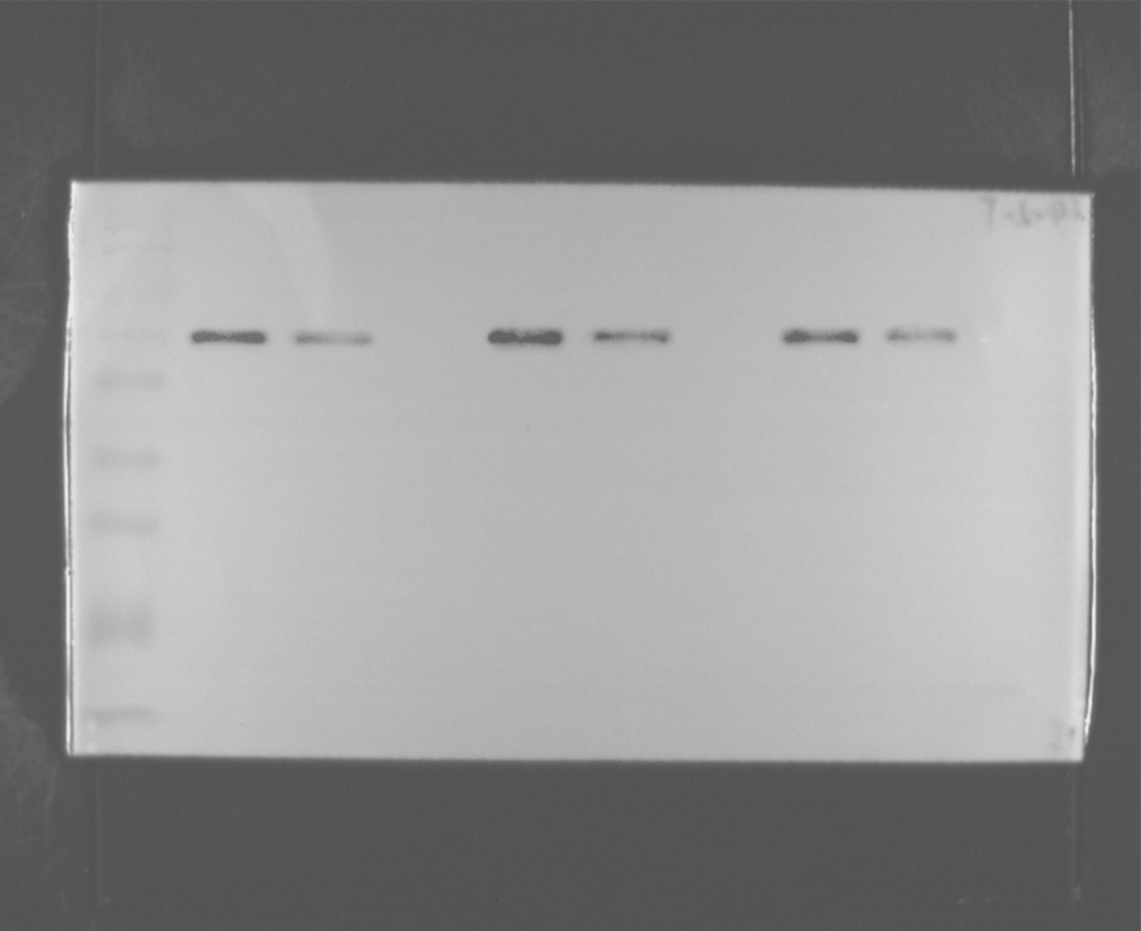

Supplement: S1 File — (ZIP) [file pone.0350815.s001.zip › Figure 2E/Parkin 55KDa 01-03.tiff]

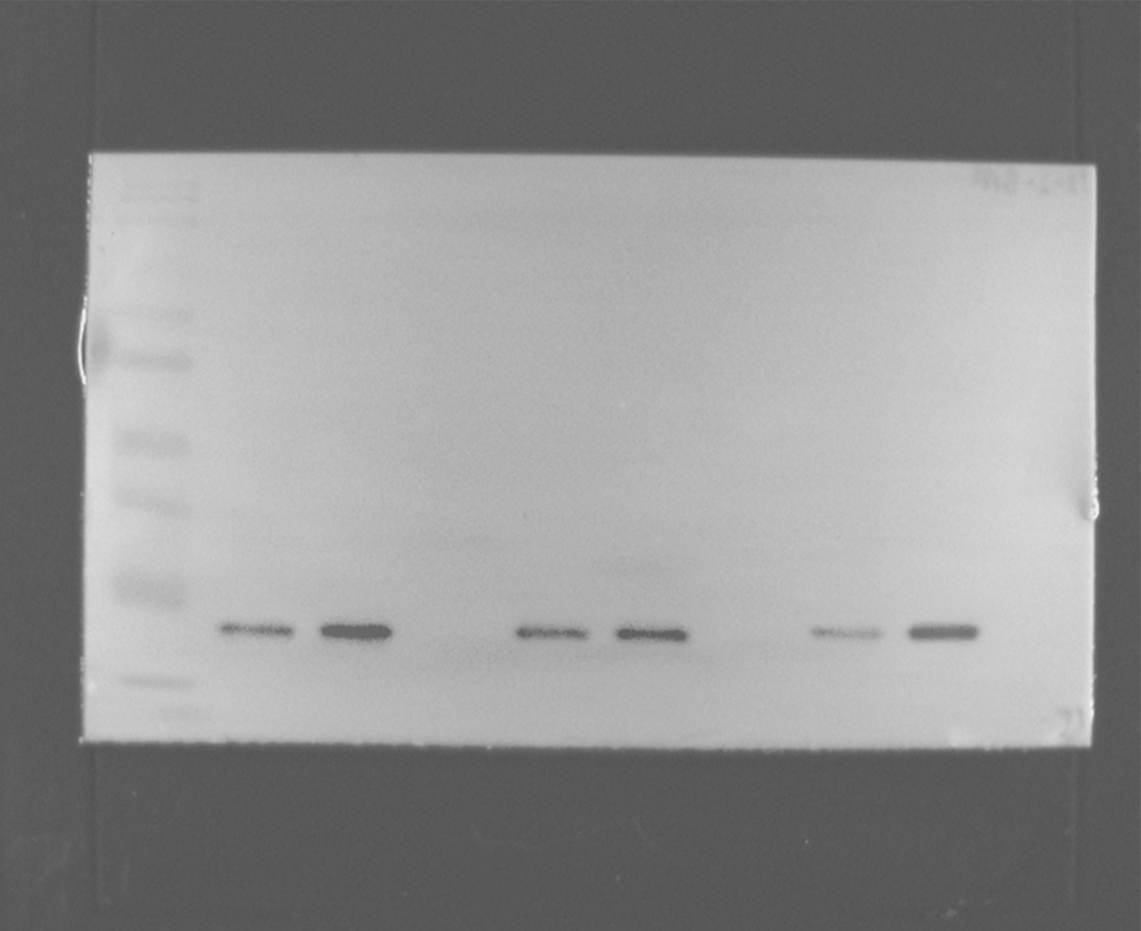

Supplement: S1 File — (ZIP) [file pone.0350815.s001.zip › Figure 2E/UBA52 15KDa 01-03.tiff]

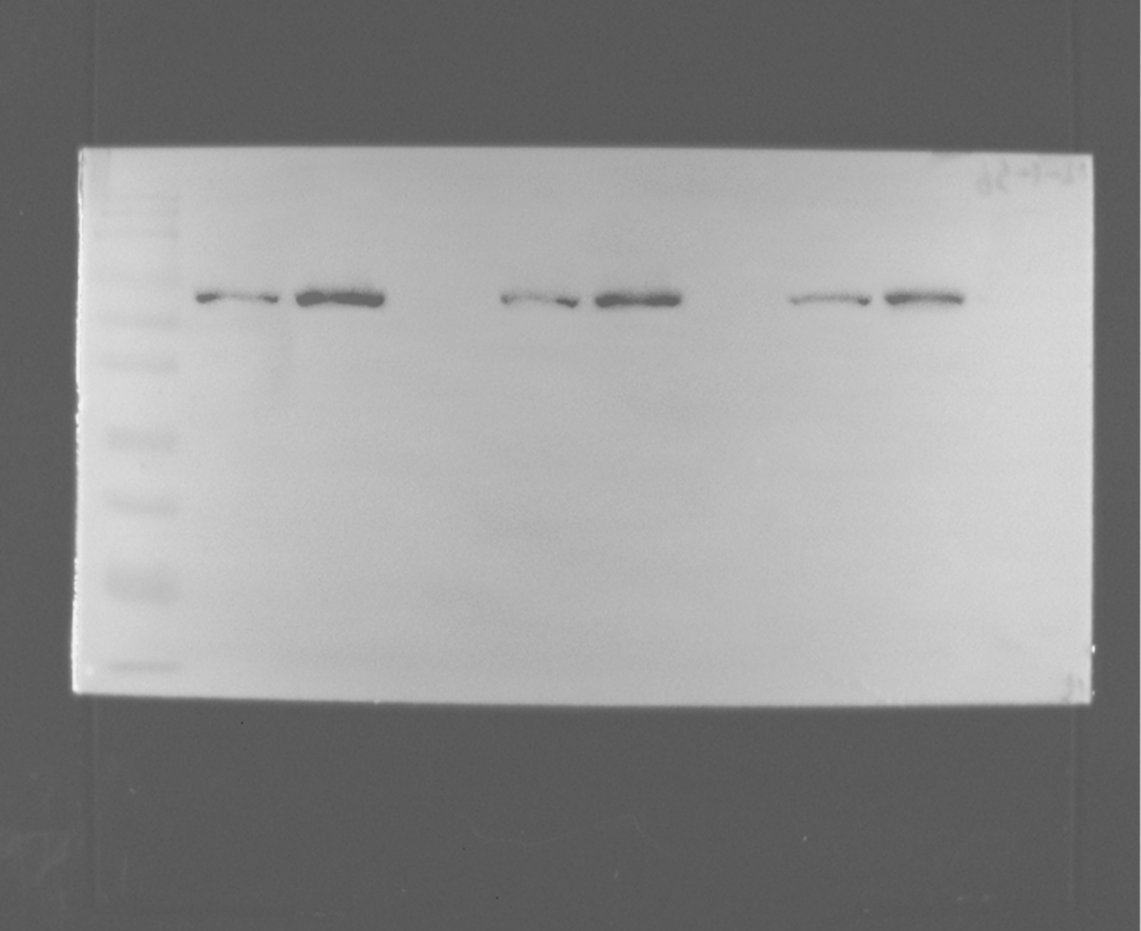

Supplement: S1 File — (ZIP) [file pone.0350815.s001.zip › Figure 2E/UBC 60KDa 01-03.tiff]

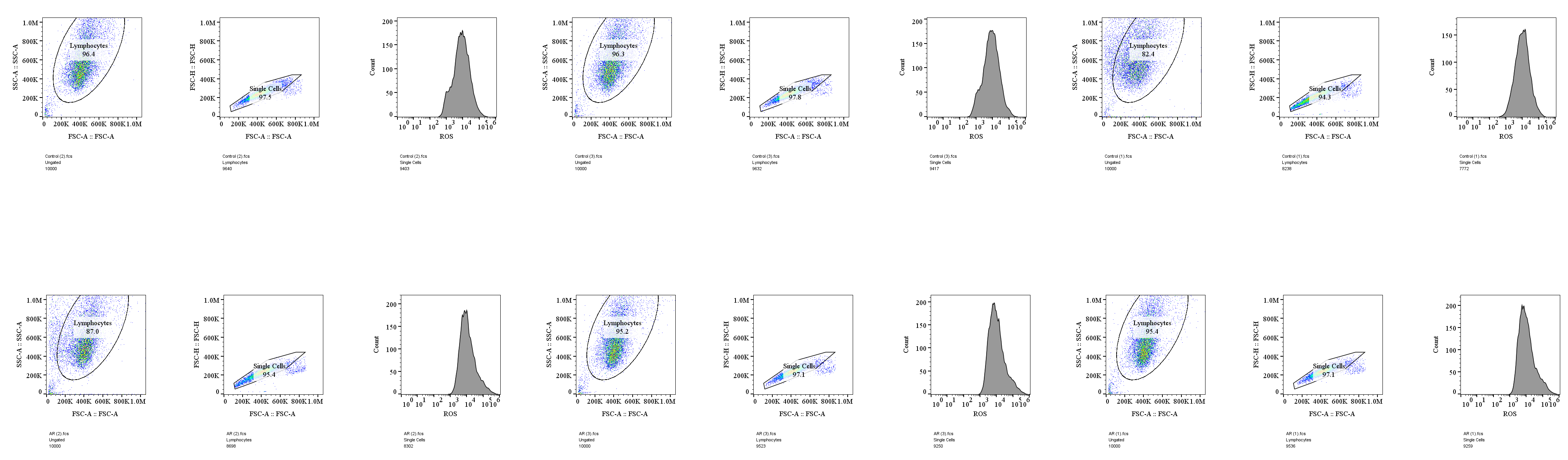

Supplement: S1 File — (ZIP) [file pone.0350815.s001.zip › Figure 3A/Data.tiff]

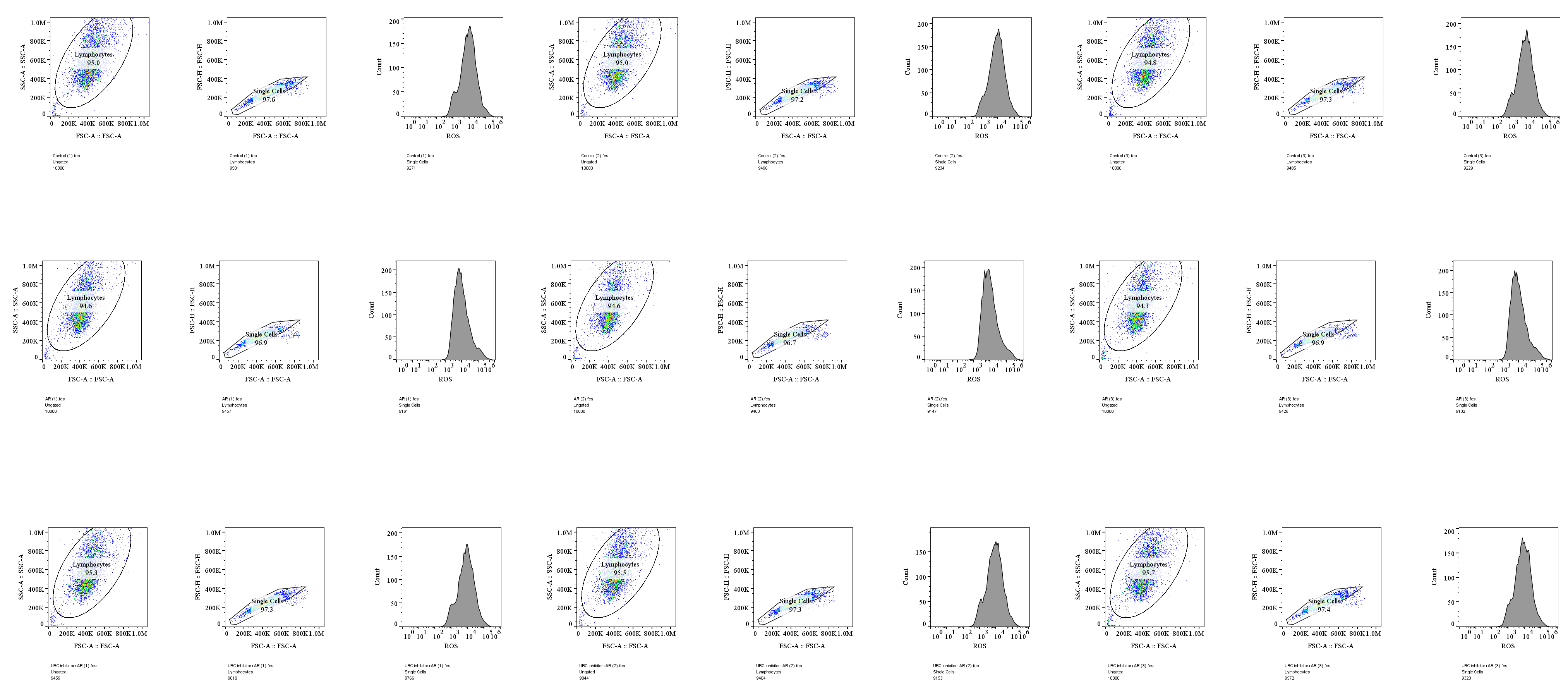

Supplement: S1 File — (ZIP) [file pone.0350815.s001.zip › Figure 5A/Data.tiff]
