# Supplementary material for: UBC/UBA52 silencing restores PINK1-Parkin-mediated mitochondrial autophagy in allergic rhinitis
Source: PLoS One. 2026 Jun 10;21(6):e0350815. doi: 10.1371/journal.pone.0350815 (PMC13252774; doi:10.1371/journal.pone.0350815)

Marker band

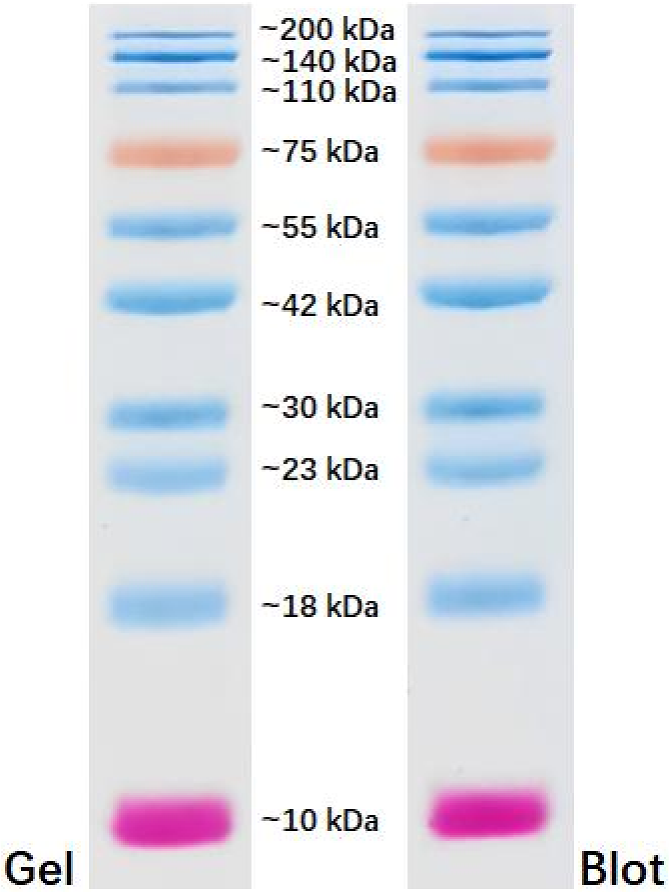

12% Tris-Glycine SDS-PAGE

Figure 2E

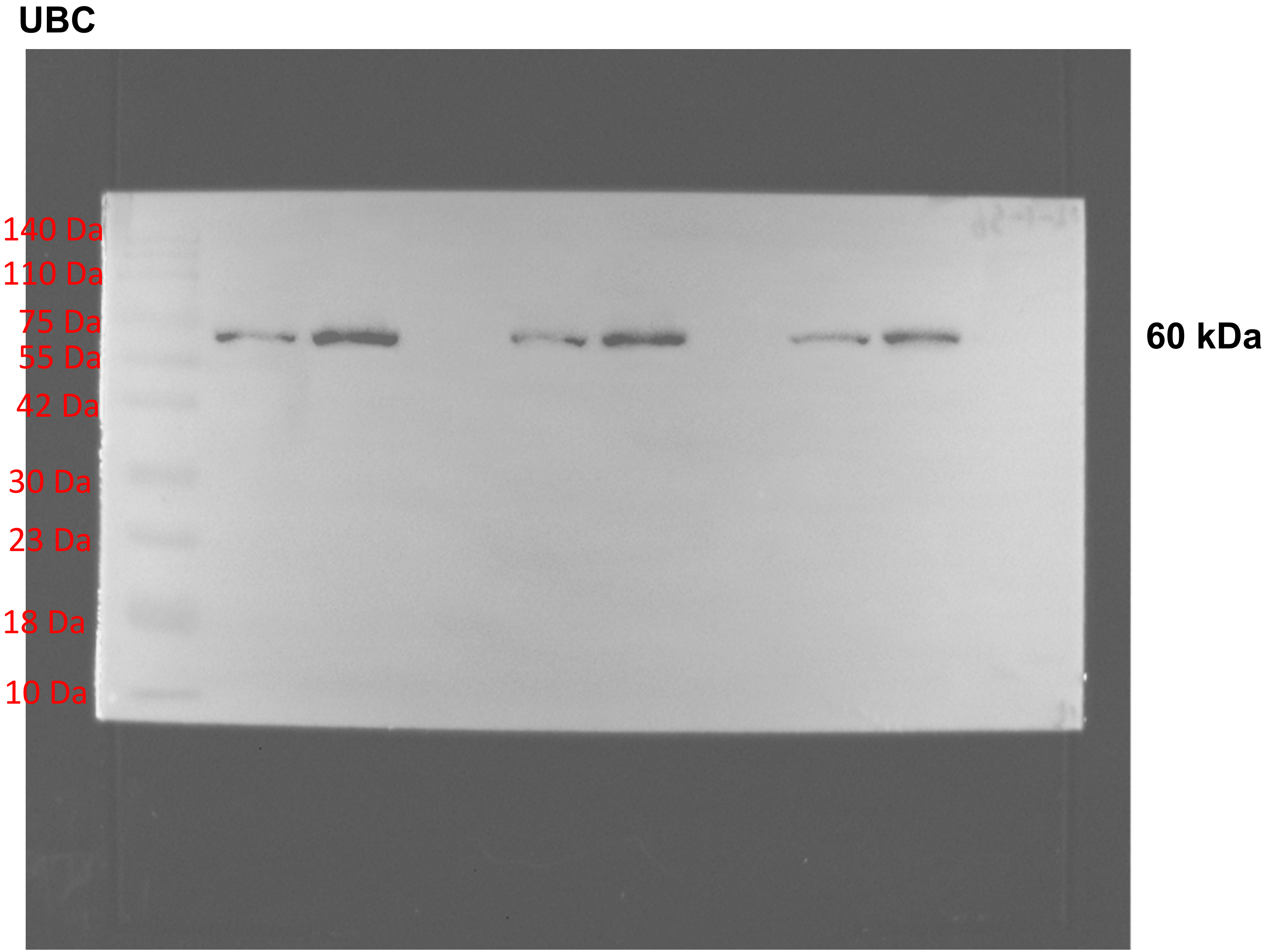

UBA52

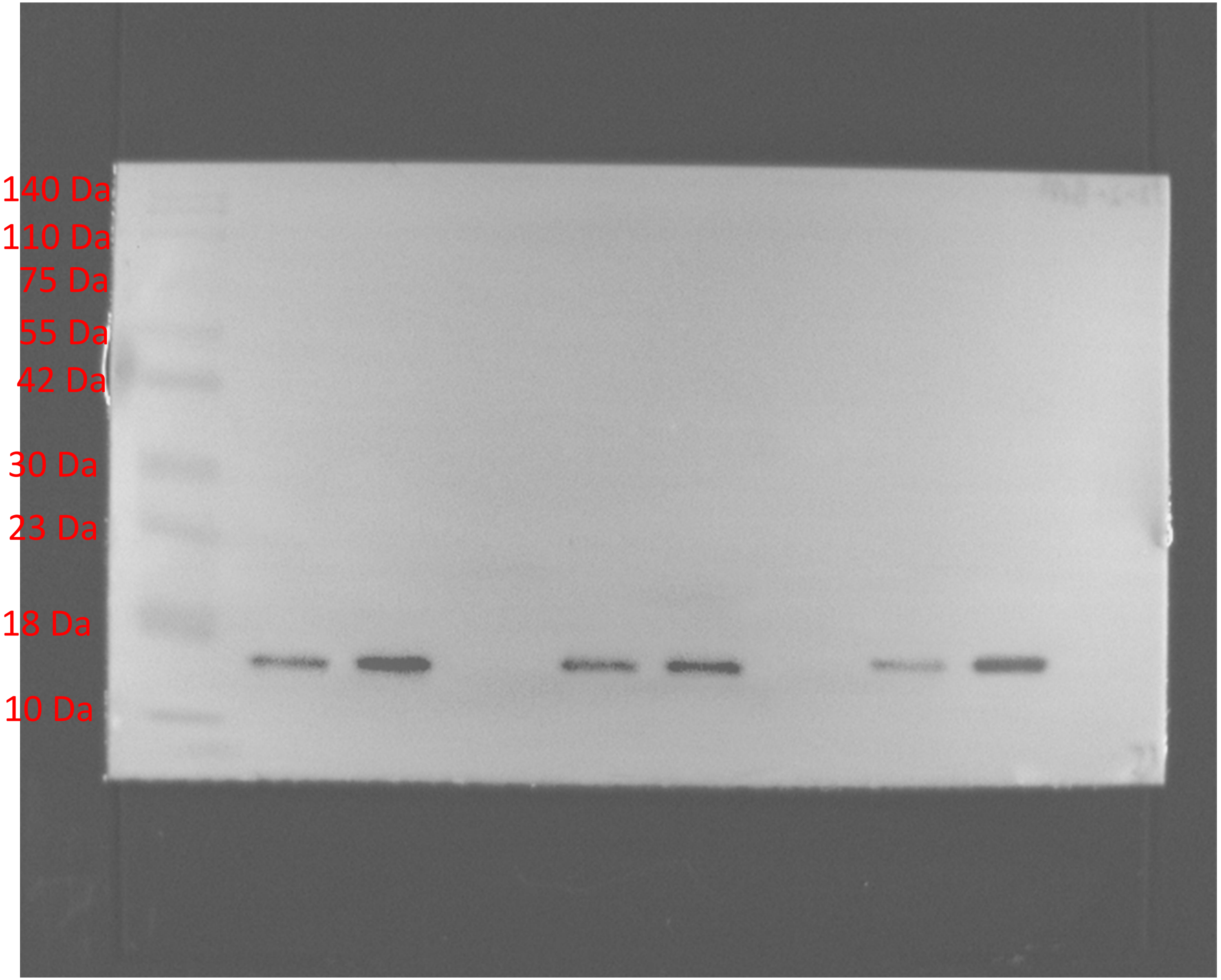

PINK

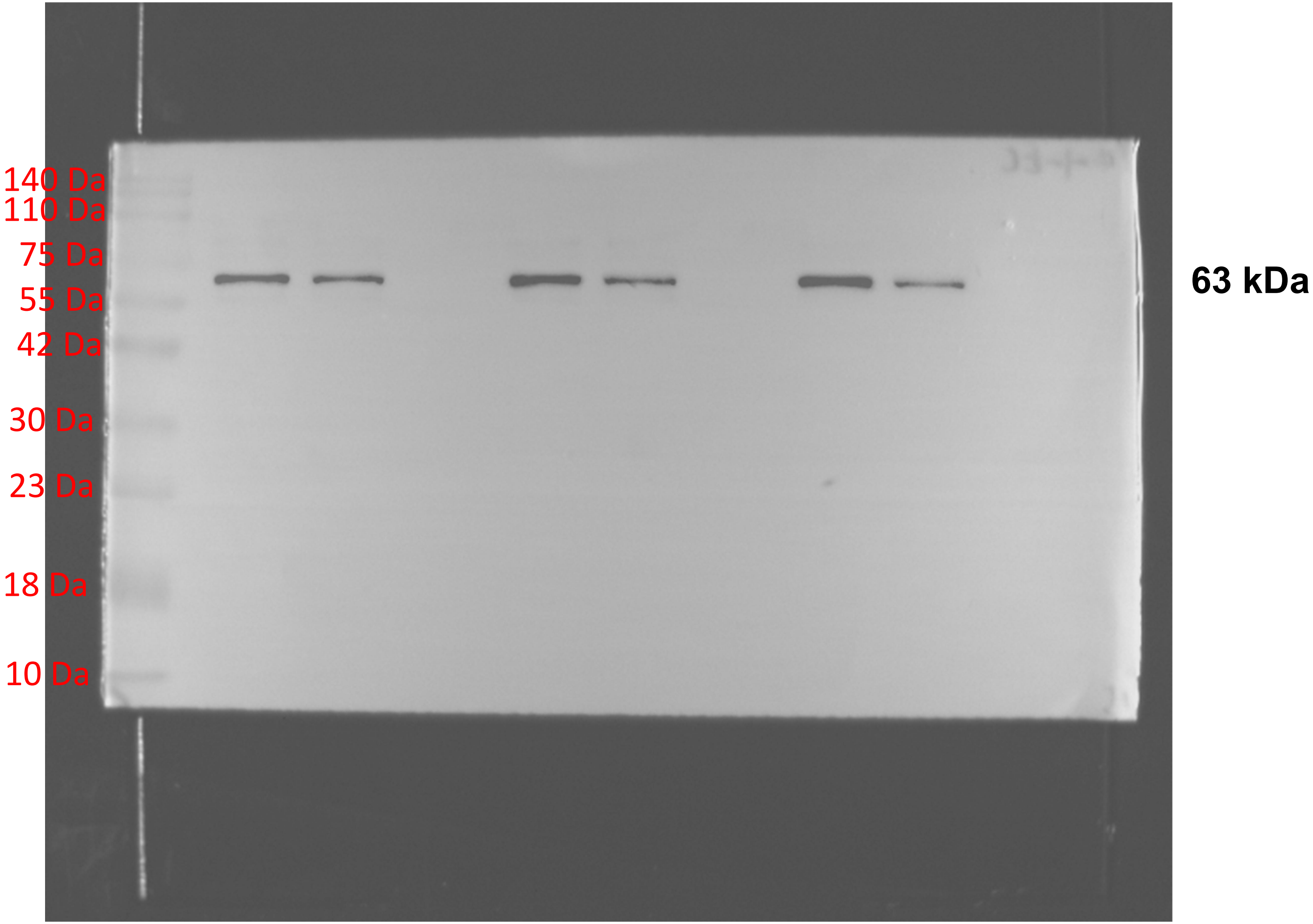

Parkin

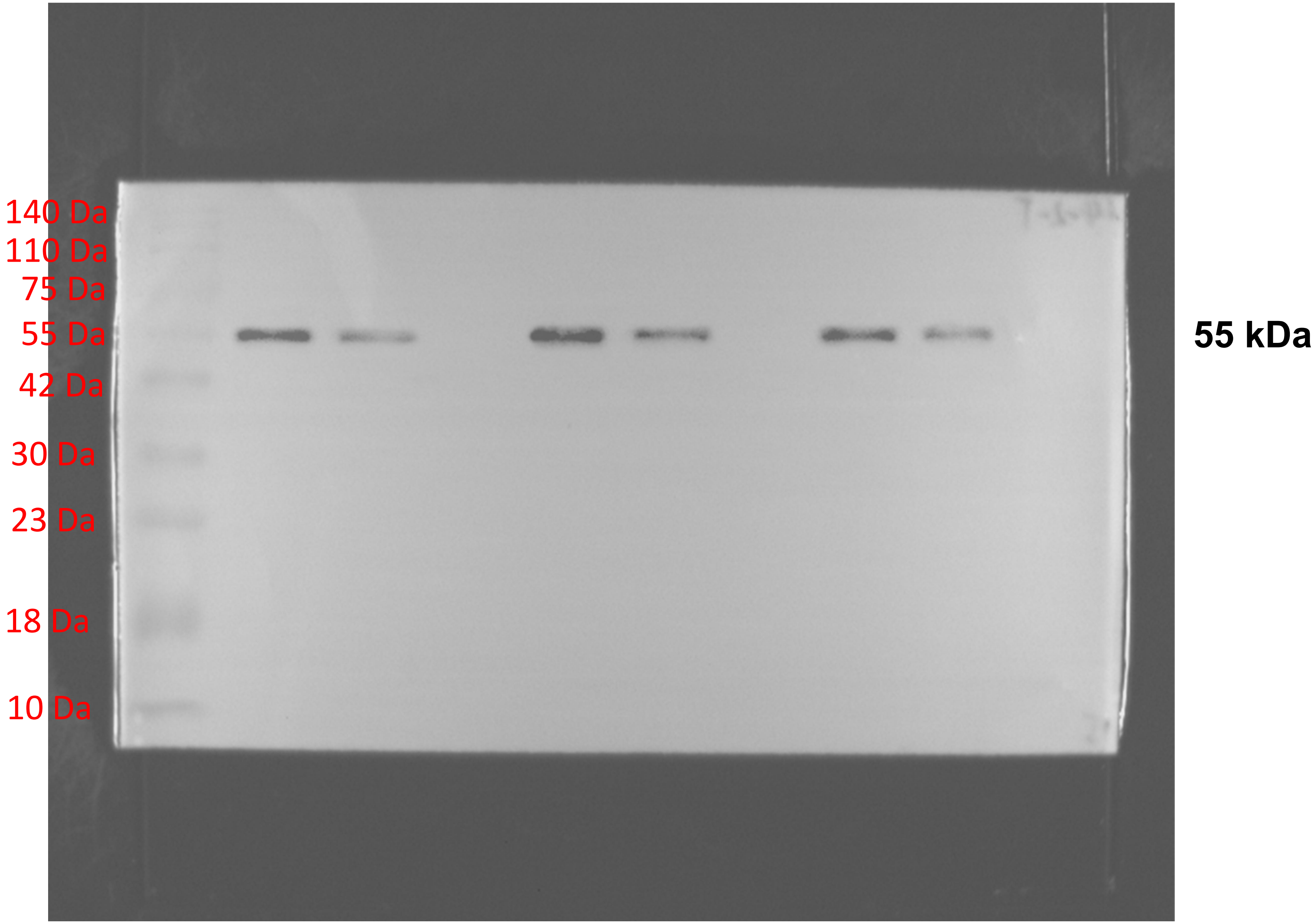

GAPDH

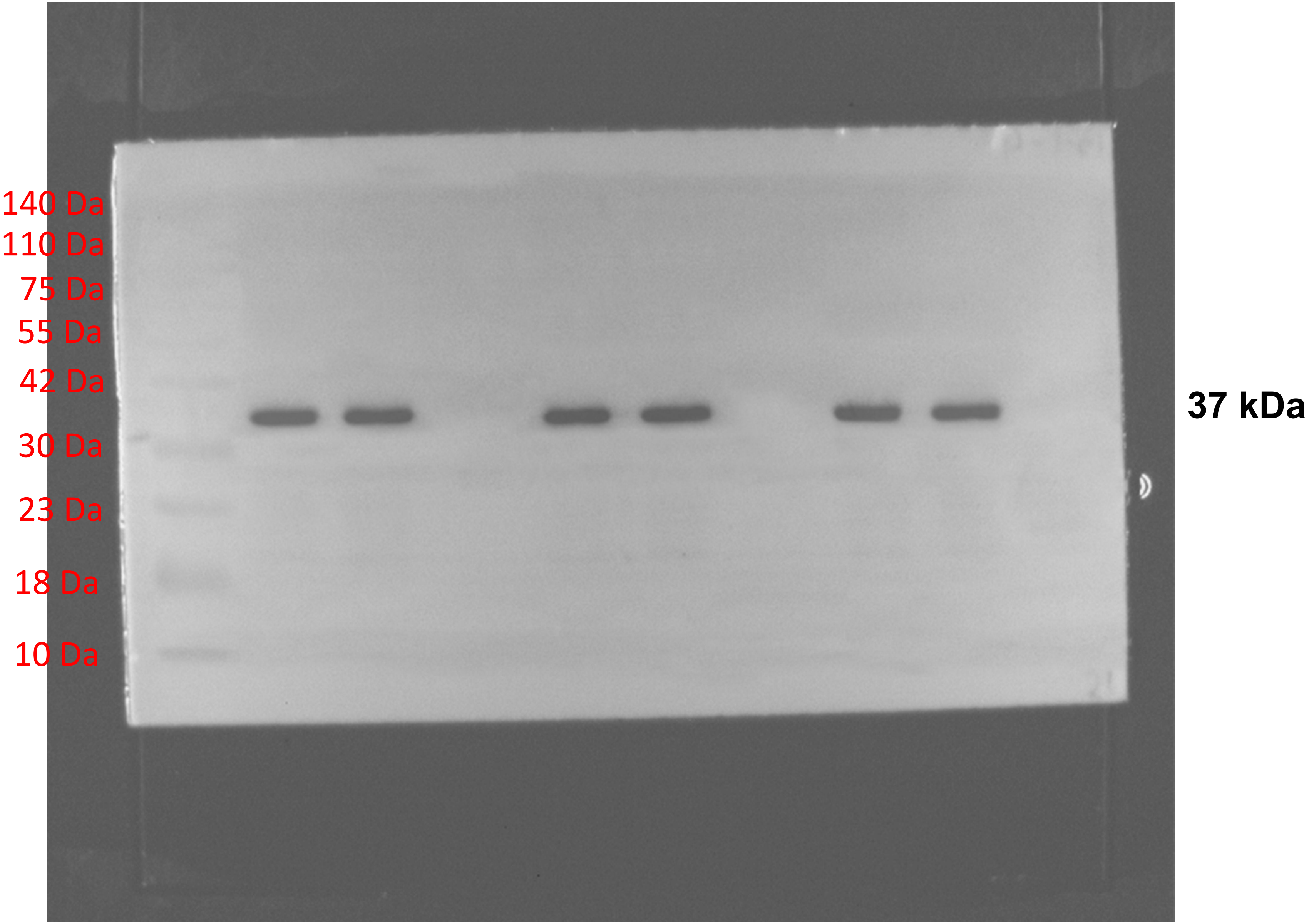

Figure 4F

UBC

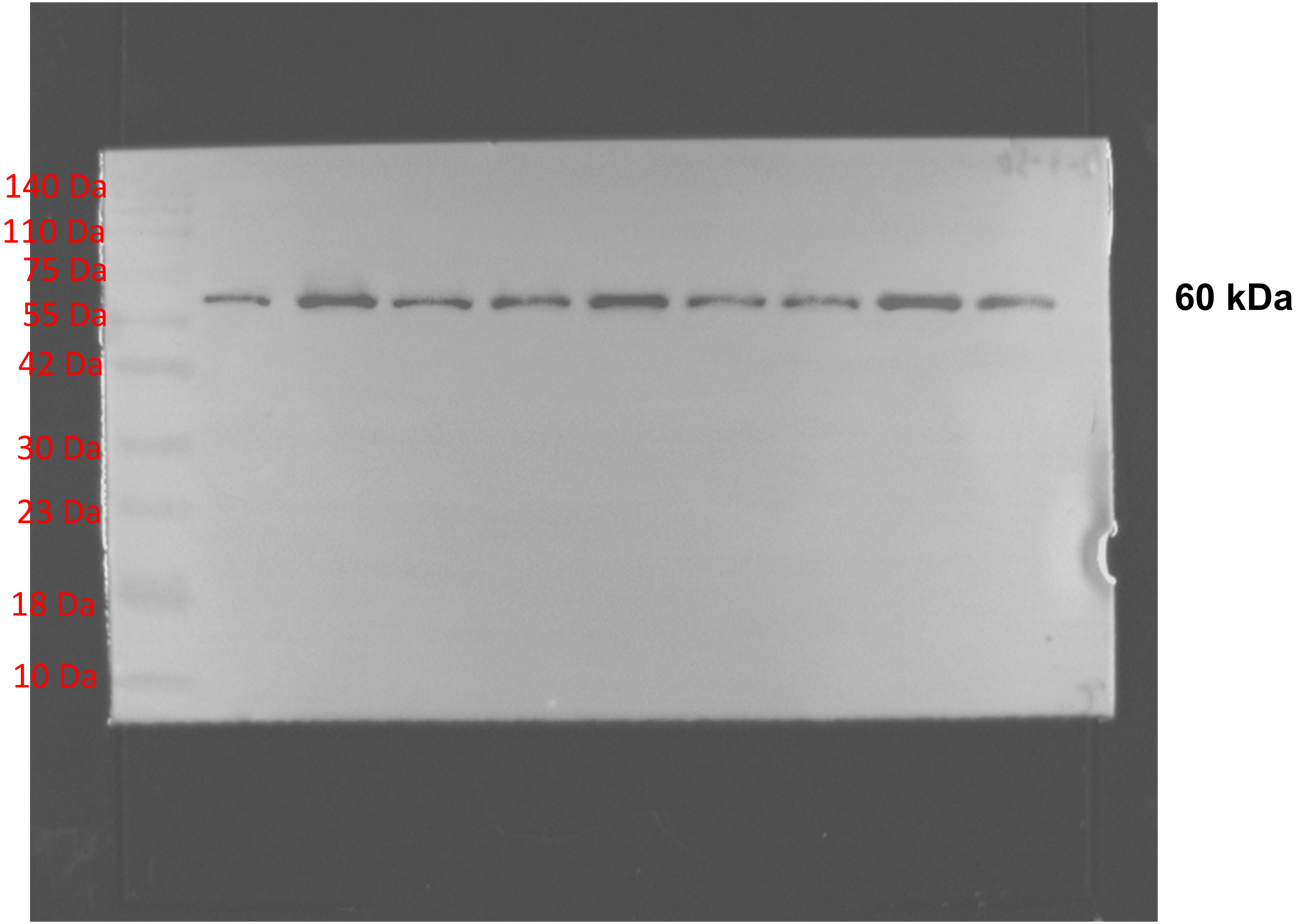

UBA52

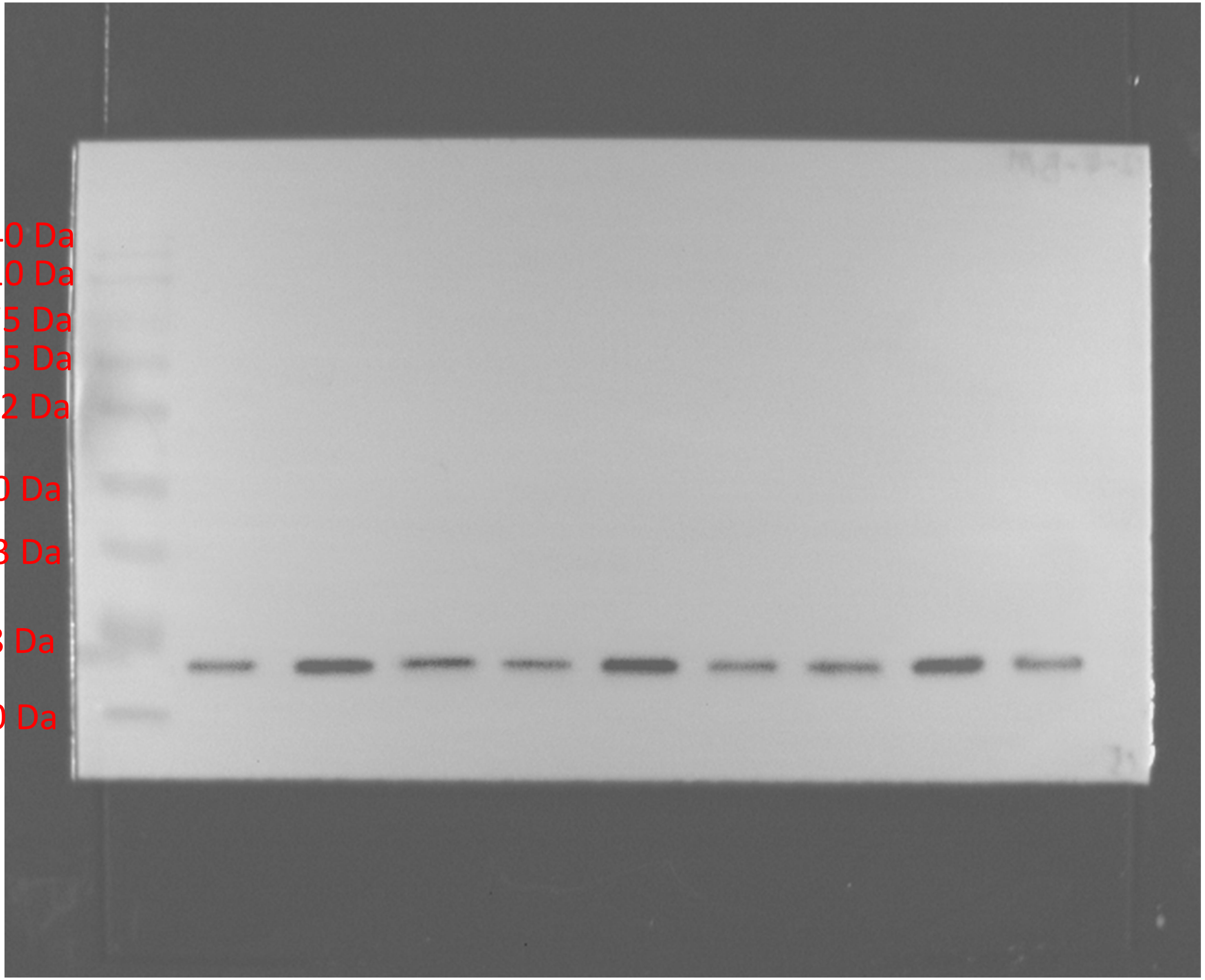

PINK

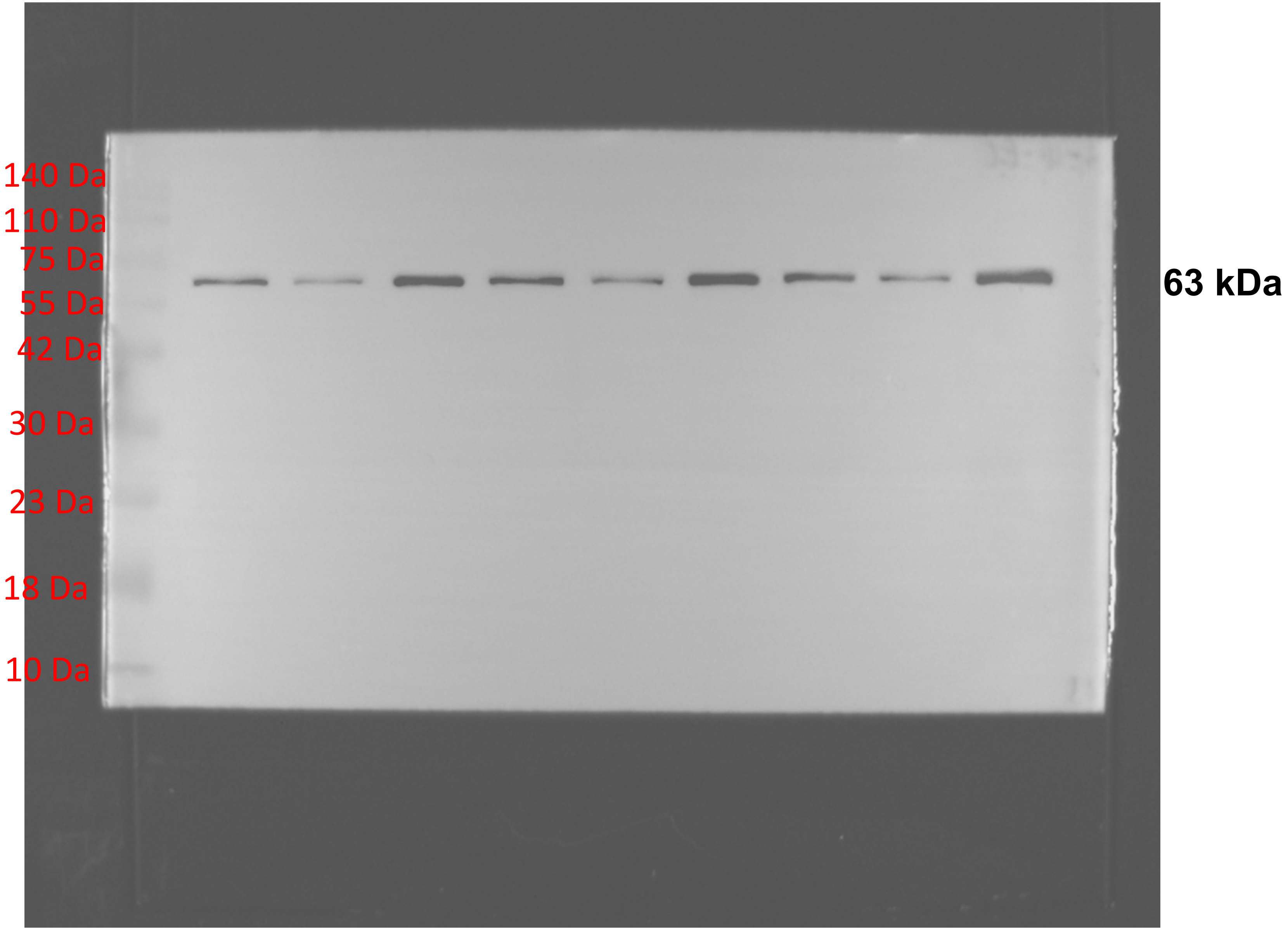

Parkin

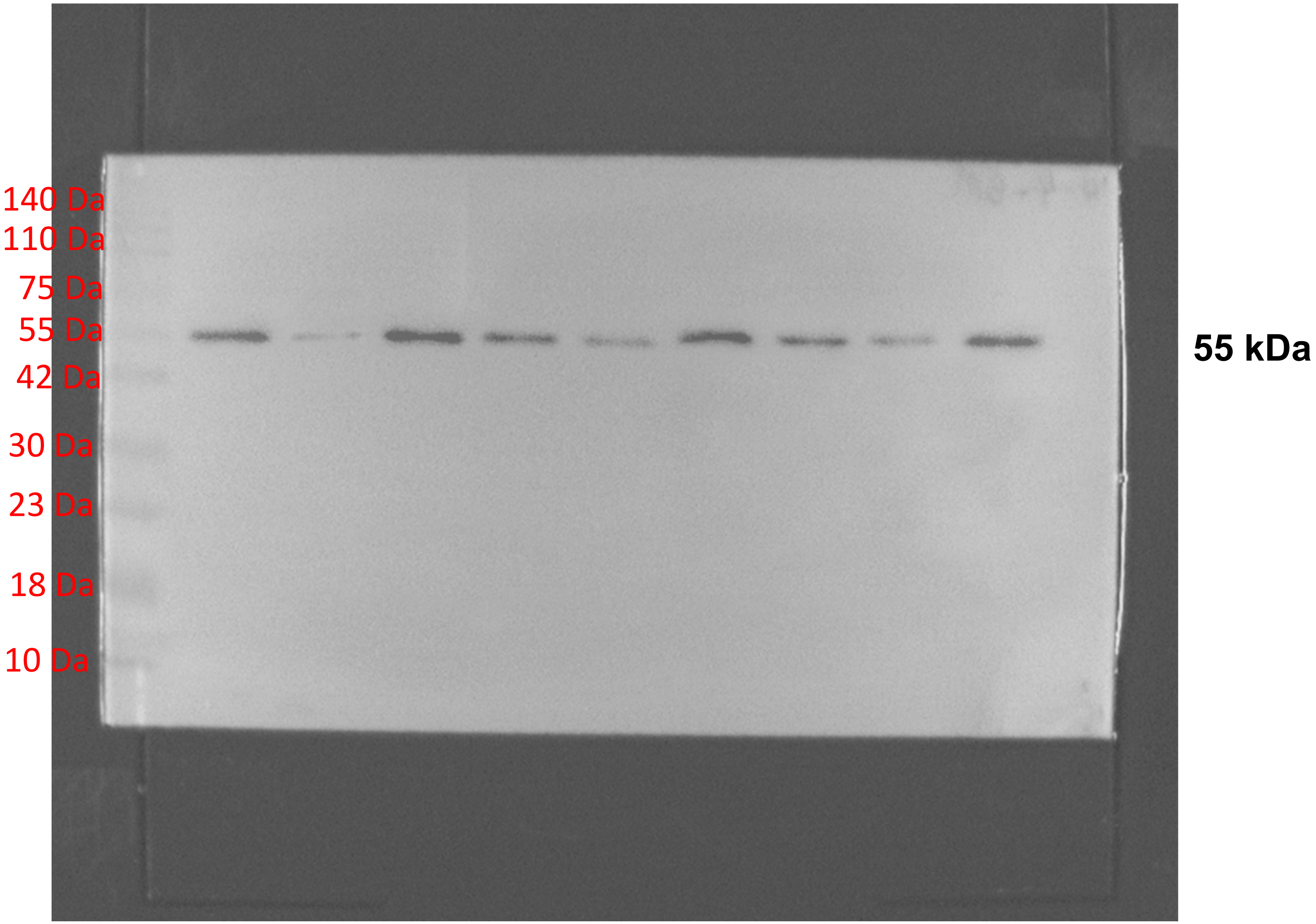

GAPDH

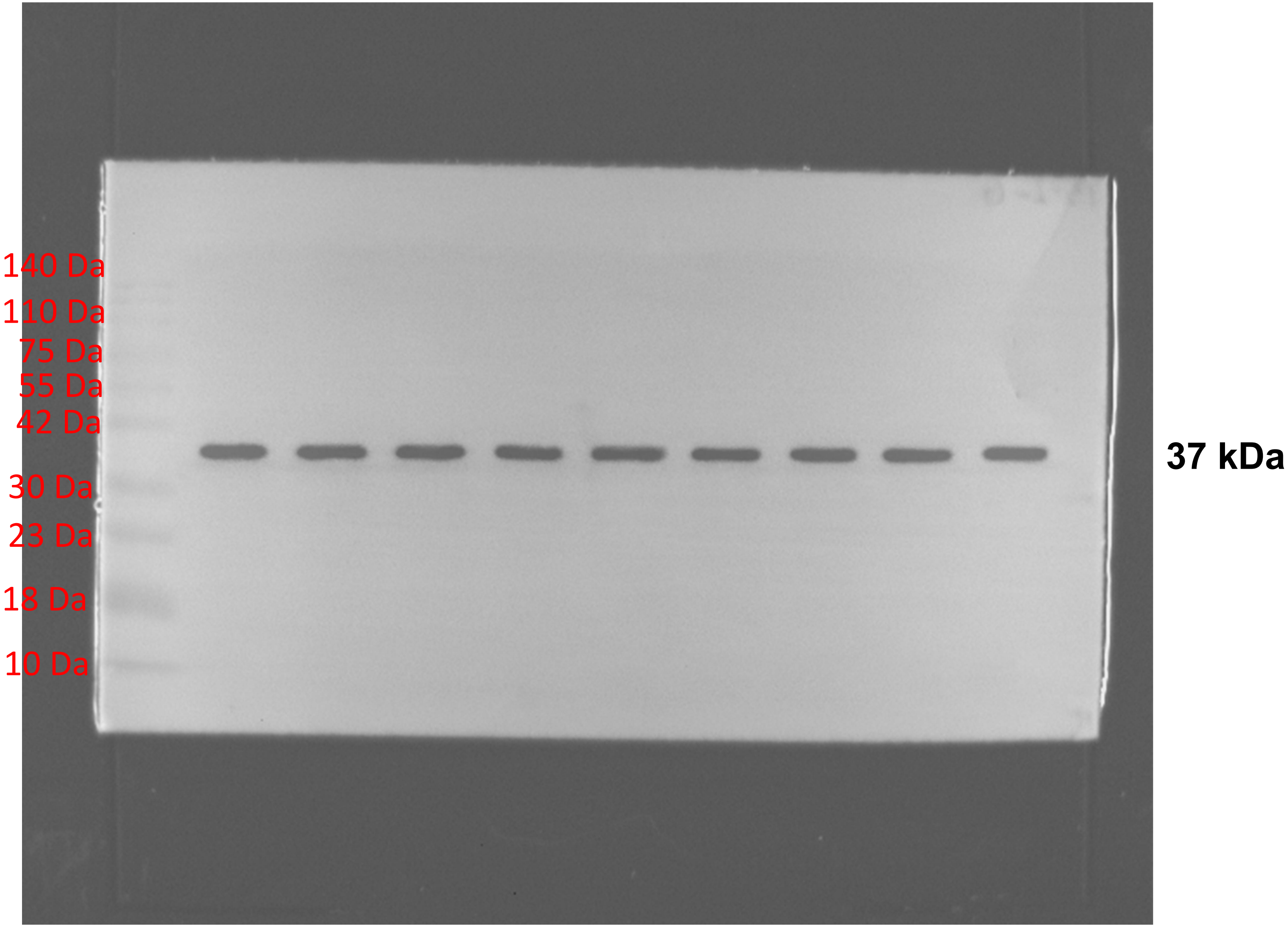

Supplement: S2 File — (PDF) [file pone.0350815.s002.pdf]
